# Machine learning-based prediction of anxiety disorders using blood metabolite and social trait data from the UK Biobank

**DOI:** 10.1016/j.bbih.2025.101010

**Published:** 2025-05-08

**Authors:** Annabel Smith, Jack J. Miller, Daniel C. Anthony, Daniel E. Radford-Smith

**Affiliations:** a Department of Pharmacology, University of Oxford, Mansfield Road, Oxford, OX1 3QT, UK; b Oxford Centre for Magnetic Resonance Research, University of Oxford, Oxford, UK; c Department of Clinical Medicine, Aarhus University, Aarhus, Denmark

**Keywords:** Anxiety, Neuroticism, Metabolomics, Random forest

## Abstract

Anxiety disorders are the most prevalent type of mental health disorders and are characterised by excessive fear and worry. Despite affecting one in four individuals within their lifetime, there remains a gap in our understanding regarding the underlying pathophysiology of anxiety disorders, which limits the development of novel treatment options. Exploring blood-based biomarkers of anxiety disorder offers the potential to predict the risk of clinically significant anxiety in the general population, increase our understanding of anxiety pathophysiology, and to reveal options for preventative treatment. Here, using psychosocial variables in combination with blood and urine biomarkers, reported in the UK Biobank, we sought to predict future anxiety onset. Machine learning accurately predicted (ROC AUC: ∼0.83) ICD-10-coded anxiety diagnoses up to 5 years (mean 3.5 years) after blood sampling, against lifetime anxiety-free controls. Analysis of the blood biochemistry measures indicated that anxious individuals were more anaemic and exhibited higher levels of markers of systemic inflammation than controls. However, blood biomarkers alone were not predictive of resilience or susceptibility to anxiety disorders in a subset of individuals rigorously matched for a wide range of psychosocial covariates (ROC AUC: ∼0.50). Overall, we demonstrate that the integration of biological and psychosocial risk factors is an effective tool to screen for and predict anxiety disorder onset in the general population.

## 1 Introduction

Anxiety disorder is a common psychiatric condition and leading cause of disability, characterised by an unwarranted, excessive, and prolonged fear response. The disorder is divided into sub-classifications, including generalised anxiety disorder; social anxiety; phobias; and panic disorders, which range in terms of severity and symptoms. According to the World Health Organization's (WHO) global mental health survey, one in four individuals will be affected by an anxiety disorder during their lifetime (Kessler et al., 2007; World Health Organisation, 2017). These disorders have a major impact on quality of life, translating to a considerable burden on society; in 2010, anxiety disorders cost over €74 billion in Europe due to time lost at work (Wittchen et al., 2011). The aetiology of anxiety disorder is multifactorial, with a range of genetic and environmental influences conferring risk. The chance of developing anxiety disorder is higher in females, and in those with a family history of anxiety disorder, childhood maltreatment, and emotional trauma as an adult, as well as with sociodemographic factors such as unemployment (Blanco et al., 2014). Whilst the frontline treatment, SSRIs, are generally efficacious, approximately 50 % of individuals with generalised-anxiety disorder are treatment resistant (Ansara, 2020). This is partially due to a lack of understanding on the pathophysiology of the disorder, the high level of heterogeneity between cases, and the lack of targeted therapeutics.

A biomarker profile for screening and monitoring anxiety disorder would facilitate diagnosis, which remains a clinical challenge as anxiety is often associated with concurrent physical symptoms. Thus far, the majority of anxiety disorder research focuses on neural circuitry; for example, pathways connecting the amygdala, hippocampus, and prefrontal cortex are known to play an important role in the acquisition, extinction, and renewal of fear responses relevant to anxiety (Xia and Kheirbek, 2020). Whilst central nervous system (CNS) studies provide a direct insight into the cognitive state of individuals, brain tissue samples are typically only accessible during autopsies, MRI scans can be claustrophobic, and cerebrospinal fluid (CSF) sampling often requires a lumbar puncture which many are averse to. However, sampling of peripheral blood enables monitoring of the pathogenesis of disorders with minimal invasiveness. Finding adequate biomarkers of anxiety disorders is regarded as one of the most important unmet needs in the field of psychiatry, with diagnosis of anxiety disorder currently relying solely on subjective symptom reporting (Katahira and Yamashita, 2017). Most searches for blood-borne biomarkers have yielded disappointing results and have added little to our understanding of pathophysiology because they have been largely driven by the ‘streetlight effect’ and have concentrated on the neurotransmitter systems implicated by the current treatment options (Vismara et al., 2020). Therefore, to aid diagnostic and prognostic insight, it is crucial to expand the currently limited understanding of blood-based biomarkers of anxiety disorder.

Although anxiety and depression often co-occur and share risk factors, our primary aim was to identify biomarkers specific to anxiety. Including depression-related variables could confound anxiety-specific signals. However, the psychosocial variables used here—such as trauma, neuroticism, and life stressors—also predict depression, as shown in our prior work (Radford-Smith et al., 2023), supporting the generalisability of our model across internalising disorders.

In addition, there is currently a lack of physiological understanding of anxiety ‘resilience’, corresponding to individuals with identical psychosocial, socioeconomic, and demographic risk factors to the anxious population at baseline who are not prospectively anxious. By determining markers of resilience, we expected to reveal novel pathways for intervention in anxious individuals and prevention in the general population. Here, we aimed to identify biological, demographic, and social factors that confer susceptibility or resilience to the development of anxiety disorder. Firstly, by controlling for a wide range of psychosocial features (demographic and life stressors), we aimed to identify whether machine learning could distinguish between anxious cases and lifetime anxiety-free resilient and general population controls. Lastly, as traumatic history is known to be a risk factor for anxiety disorder and as neuroticism was found to be important in predicting depression disorder, we sought to determine how neuroticism score and trauma history interact with the blood biomarker data to influence anxiety disorder development (Blanco et al., 2014; Radford-Smith et al., 2023). Through identifying the most important risk factors, we demonstrate novel associations between anxiety risk, psychosocial factors, and blood biomarkers, which may serve to inspire future treatment interventions and preventative care.

## 2 Methods

### 2.1 Study population

The UK Biobank encodes the prospective health and lifestyle of approximately 500,000 individuals (Sudlow et al., 2015). The UK Biobank study was approved by the Northwest Multi-Centre Research Ethics Committee, with all participants providing written and informed consent. Participants were recruited between 2006 and 2010 (aged between 37 and 73 years at baseline).

**Fig. 1:**
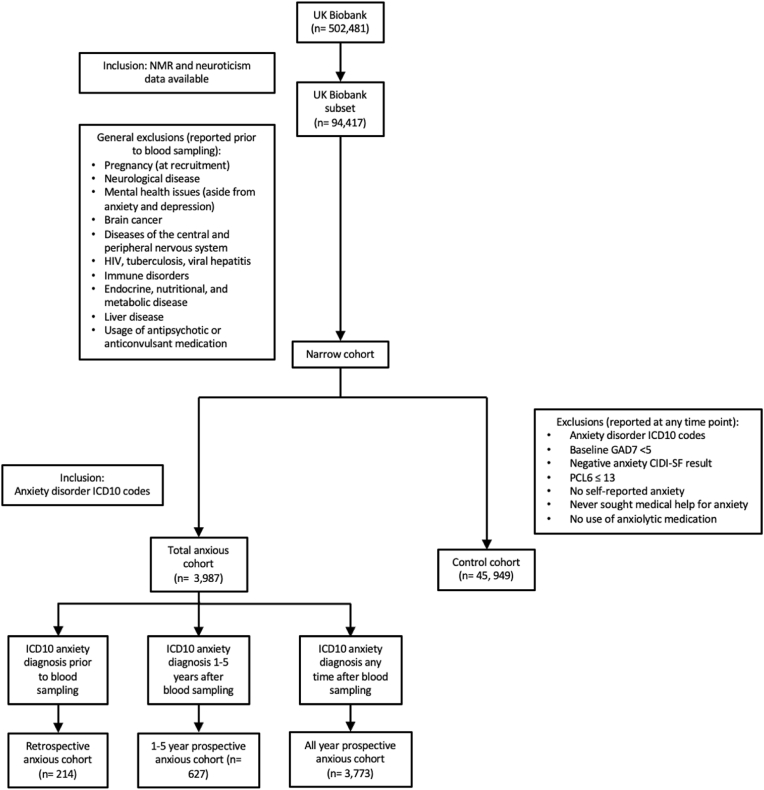
Flowchart of inclusion and exclusion criteria for study participants.

This study includes participants for which a neuroticism score and blood metabolite data were available at baseline (n ∼ 100,000; Fig. 1). Quality control of technical variation within the NMR data was performed using the R package, “ukbnmr” (Ritchie et al., 2023). The 12-item neuroticism assessment is based on the Eysenck Personality Questionnaire-Revised (EPQ-R) Short Form (Supplementary Table S1) (Eysenck et al., 1985).

To mitigate the influence of confounding factors on blood metabolite composition, a rigorous general exclusion criterion was applied for participants included in this study (Fig. 1, see Supplementary Table S2 for further details). These exclusions encompassed conditions, including pregnancy, neurological diseases, viral diseases, endocrine, nutritional, and metabolic diseases that were diagnosed prior to blood sampling. Additionally, individuals with mental health disorders other than anxiety or depression were excluded (Supplementary Table S2), generating the “narrow cohort” (Fig. 1). Comorbid depression was not used as an exclusion criterion, reflecting its high co-occurrence with anxiety in the general population.

To classify the anxious population, hospital ICD-10 linkage data were utilised, providing a comprehensive record of various disorders and their respective dates of diagnosis in individuals. Participants with at least one anxiety disorder diagnosis (F40-43) were incorporated into the anxious cohort (Fig. 1) (Kennedy and Niedzwiedz, 2022). The anxious group was further divided into a ‘retrospective anxious’ cohort (n = 214), comprising individuals first diagnosed with an anxiety disorder prior to blood sampling, a prospective anxious cohort (n = 3773), and those first diagnosed only up to 5 years after sampling also included within the ‘1-5-year prospective anxious’ cohort (n = 627). The control cohort (n = 45,949) was created by processing individuals through a stringent list of exclusion criteria, ensuring no evidence of anxiety through a formal diagnosis, medication usage, or self-reported answers to anxiety related questions (Fig. 1, Supplementary Table S2). Comprehensive demographic information were compared between the three anxious sub-cohorts and the lifetime anxiety free control cohort (Supplementary Table S3–5). Group differences were assessed using the chi-square test and the unpaired *t*-test for categorical and numerical data respectively.

Each anxious cohort was further assessed for the relative composition of each anxiety disorder sub-classification (such as generalised anxiety disorder and social anxiety disorder) within the population (Supplementary Table S6).

### 2.2 Identification of resilient control groups

To control for confounders, demographic factors that significantly differed between the anxious sub-cohorts and the control group were used to match the populations (variables listed in Supplementary Table S3–5). The matching process ensured that differences in covariates are equal between groups, as would be the case in a randomised controlled trial.

Matching was performed in R using the “MatchIt” package v.4.4.0, whereby each anxious sub-cohort was independently matched to a control cohort (Ho et al., 2011). Missing numerical values were imputed in R using the function “na.roughfix” (under the “randomForest” package v.4.7.1.1) (Breiman, 2001). Missing categorical data were imputed ‘Unknown’. Matching was performed sequentially to ensure controls matched to an anxious sub-cohort were not used for matching in the following anxious sub-cohort, resulting in no duplications across resilient control groups. Matching was performed in a 1:1 ratio between control and anxious individuals, with propensity scores set to a caliper of 0.2.

Absolute standardised mean differences across the demographic factors between each anxious subpopulation and their control groups were assessed before and after matching to reveal matching quality (Supplementary Fig. S1–3). New demographic tables were generated across each cohort post-matching, showing no group differences (Supplementary Table S7–9).

### 2.3 Random forest methods

#### 2.3.1 Machine learning classification of anxiety disorder

Supervised random forest methods were used to classify anxious and resilient/control populations, using 382 blood and 4 urine biomarker variables (NMR metabolomics [Category ID 220], urine assays [Category ID 100083], blood count [Category ID 100081], and blood biochemistry [Category ID 17518]) and the “randomForest” v.4.7.1.1 package. See supplementary methods.

The number of trees for each random forest model was set to 500 to obtain an optimal balance between accuracy and computational speed. The ‘mtry’ parameter within the random forest function, defining the number of predictors randomly sampled at each split of a node, was set to the square root of the number of predictor variables included in the model.

**Fig. 2:**
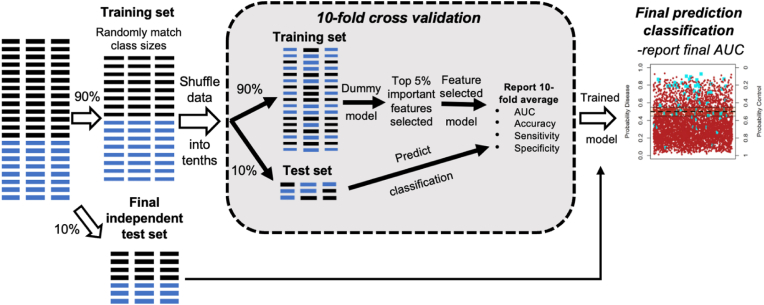
Random forest modelling experimental workflow.

Each model comprised an anxious sub-cohort with their respective control, was randomly split with 90 % allocated to a training set and the remaining 10 % as an independent final test set (Fig. 2). The training data was subject to a 10-fold cross validation procedure with 10 repeats, resulting in an ensemble of 100 random forest models. Each model was comprised of an equal number of randomly selected anxious and control individuals (to ensure balance as the sample sizes were not equal). At each fold of cross validation, a feature selection stage was applied. Initially, a random forest model was generated on the training data using all predictor variables. Subsequently, the top 5 % most important predictors, as ranked by their mean decrease in Gini coefficient scores, were fed into a new “pruned” random forest model that was then used to predict the test set. Across the 100 models, the average accuracy, sensitivity, and specificity, based on the predicted class compared to the actual class, were recorded. The area under the curve (AUC) values, a measure of the ability of binary classifiers to accurately distinguish between positive and negative classes, were recorded. These predictive models were evaluated against models representing the null distribution, whereby the class of each individual (anxious or control) was randomly permuted (ROC AUC ∼ 0.50). Finally, the trained random forest model was applied to predict the full independent test set (10 % of the data), for which class sizes were not matched.

For further details regarding random forest data processing and feature selection, neuroticism stratification, and incorporating history of trauma into predictive anxiety modelling, see supplementary methods.

## 3 Results

### 3.1 Machine learning was unable to distinguish resilient and anxious individuals based on blood biomarkers alone

Upon controlling for potential confounders and using 381 blood biomarkers and 4 urine biomarkers as predictor variables to the random forest algorithm, the machine learning models were unable to accurately classify anxious sub-cohorts and their matched ‘resilient’ control groups (Table 1).

**Table 1:** Mean 10-fold cross-validated ROC AUC, accuracy, sensitivity, and specificity values of random forest models trained with anxious sub-cohorts are equivalent to models trained with randomly assigned classes.

	Retrospective anxious vs matched control		1–5-year prospective anxious vs matched control		All year prospective anxious vs matched control	
	RF output	Null distribution RF output	RF output	Null distribution RF output	RF output	Null distribution RF output
Mean ROC AUC	0.47	0.49	0.52	0.50	0.58	0.50
Mean accuracy	0.47	0.49	0.51	0.51	0.55	0.50
Mean sensitivity	0.46	0.51	0.50	0.48	0.54	0.51
Mean specificity	0.48	0.48	0.52	0.53	0.57	0.49

Abbreviations: Random forest (RF).

**Fig. 3:**
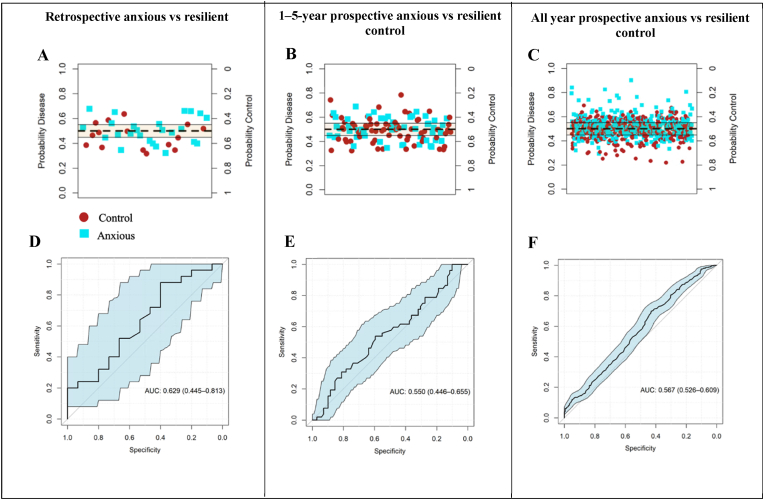
Trained random forest models were unable to accurately classify anxious cases and resilient controls*.* A,B,C) The models, across all cohorts, were unable to accurately distinguish between true anxious cases (labelled blue) and true controls (labelled red), with most probabilities falling within the 0.3–0.6 range, indicating unclear classification probability determination. D,E,F) ROC curves across the three anxious cohorts averaged approximately 0.6, indicating unclear prediction classification.

Mean 10-fold cross-validated ROC AUC, accuracy, sensitivity, and specificity of the cross-validated models was approximately 0.5 for the ‘retrospective anxious’ matched cohort and the ‘1-5-year prospective anxious’ matched cohort, similarly to the null distribution models (Table 1). There was a slight improvement in the classification of the ‘all year prospective anxious’ matched cohort; with the mean ROC AUC determined at 0.58, and a mean accuracy, sensitivity, and specificity at 55 %, 54 %, and 57 % respectively. The average out-of-bag (OOB) error was approximately 0.44 throughout the cross validations. The ROC AUC values slightly improved to approximately 0.60 across the matched anxious cohorts when applying the random forest algorithms, trained at the cross-validation stage, to an independent test set (comprising the remaining 10 % of the data) (Fig. 3). Predicting anxiety in the all years prospective cohort was not improved by stratifying by sex (Fig. S9 and S10).

### 3.2 Machine learning predicts prospective anxiety disorder with a ROC AUC of 0.83 when integrating both psychosocial and biological data

**Fig. 4:**
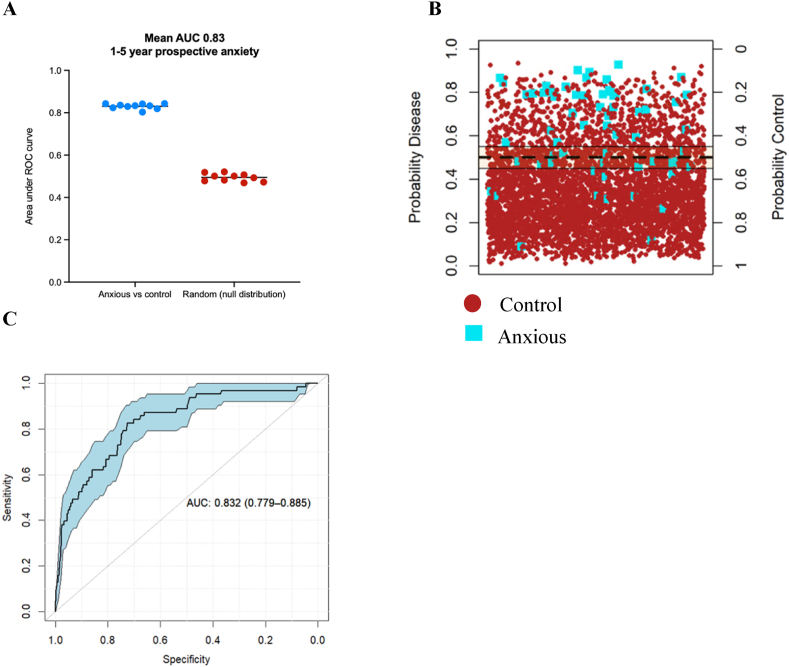
1–5-year prospective anxiety was accurately discriminated against control cases by random forest modelling when demographic, social, and biological factors are included as predictor variables. A) The random forest 10-fold cross validation accurately distinguished the ‘1-5-year prospective anxious’ cohort against the unmatched control group (mean ROC AUC: 0.83), compared to the null distribution random forest model (mean ROC AUC: 0.5). B) Application of the trained random forest algorithm to the independent final test data (comprising 10 % of the dataset) resulted in accurate prediction discrimination between prospectively diagnosed anxiety disorder up to 5 years after blood sampling (true anxious cases labelled blue) and control cases (true control cases labelled red). C) The ROC curve, summarising prediction quality at the independent testing stage, demonstrates the random forest model discriminated between ‘1-5-year prospective anxiety disorder’ against controls with a ROC AUC of 0.83 (95 % CI 0.78–0.89).

The cross-validated random forest models trained on 90 % of the data discriminated between anxiety disorder diagnosed up to 5 years (mean 3.5 years) after blood sampling and the unmatched lifetime anxiety free controls with a mean cross validated ROC AUC of 0.83, compared to the ROC AUC of 0.49 generated from the null distribution model (Fig. 4a). The mean out-of-bag (OOB) error during the cross-validation of the training set was 0.25. The average accuracy, sensitivity, and specificity during the cross-validation were 75 %, 71 %, and 79 % respectively. Further, application of the trained model to an independent test set (comprising 10 % of the data) resulted in the same ROC AUC of 0.83 (95 % CI 0.78–0.89) (Fig. 4b). Ranked features contributing the most towards the accuracy of the model are provided in Supplementary Fig. S4, with the top 7 % most important predictors, based on their frequency of appearance in the cross-validated feature selected models and their mean decrease in Gini coefficient score, provided in Table 2. The Gini index (or mean decrease in Gini) quantifies the contribution of each variable to reducing node impurity across all trees in the random forest; higher values indicate greater importance in classification, and a RF model with a higher average Gini index likely had classes that were more separable. Differences between the anxious cohort relative to the control cohort driving model accuracy, included significantly higher neuroticism in the anxious cohort, reduced self-reported health quality, reduced red blood cell related markers, and higher inflammation (Table 2, see Supplementary Table S12 for further detail). Specifically, haemoglobin concentration above 14.0 g/dL, haematocrit above 41.2 %, and red blood cell count above 4.47x10^12^/L conferred an odds ratio of 0.51 (95 % CI 0.43 to 0.59), 0.54 (95 % CI 0.46 to 0.64), and 0.52 (95 % CI 0.44 to 0.61), with red blood cell distribution width above 13.4 % conferring an odds ratio of 1.35 (95 % CI 1.15 to 1.58). C-reactive protein at 1.47 mg/L and GlycA at 0.80 mmol/L conferred an odds ratio of 2.13 (95 % CI 1.81 to 2.51) and 1.84 (95 % CI 1.57 to 2.15) respectively (see Supplementary Table S13).

**Table 2:** Summary of the key predictor variables in the classification accuracy of future anxiety disorder by the random forest algorithm.

Variable	UK Biobank ID	Direction of movement (in anxiety compared to control)	Representative mean decrease Gini coefficient (relative importance)	
			1–5 years prospective anxiety	All years prospective anxiety
Neuroticism score	20127	↑	93.7	465.4
Number of self-reported non-cancer illnesses	135	↑	31.4	122.2
Urea	30670	↓	26.3	98.5
Total bilirubin	30840	↓	24.7	112.8
GlycA	23480	↑	24.7	96.6
Townsend deprivation index	22189	↑	24.5	106.0
Alkaline phosphatase	30610	↑	23.7	101.7
Histidine	23463	↓	23.7	99.3
IGF-1	30770	↓	23.6	96.7
Monocyte (%)	30190	↓	23.2	96.5
C-reactive protein	30710	↑	23.2	94.1
Red blood cell (erythrocyte) distribution width	30070	↑	22.9	92.5
Urate	30880	↓	22.6	100.3
Platelet count	30080	↑	22.6	93.3
Creatinine (measured by enzymatic analysis)	30700	↓	22.5	108.5
Body mass index (BMI)	21001	↑	22.5	97.7
Platelet crit	30090	↑	21.9	90.9
Haematocrit (%)	30030	↓	21.8	95.1
Haemoglobin concentration	30020	↓	21.7	99.4
Number of treatments/medications taken	137	↑	21.5	111.4
Red blood cell (erythrocyte) count	30010	↓	21.4	96.7
Creatinine	23478	↓	21.3	104.4
Leucine	23466	↓	21.2	90.7
Total concentration of branched-chain amino acids	23464	↓	21.2	88.9
Overall health rating (%)	2178	↓	10.7	46.4
Pain	6159	↑	10.4	39.5
Long-standing illness, disability or infirmity	2188	↑	9.9	45.5
Too sick for employment	6142	↑	6.5	NA

Note: ↑ refers to an increase in anxious individuals compared to controls, ↓ refers to a decrease in anxious individuals compared to controls.

**Fig. 5:**
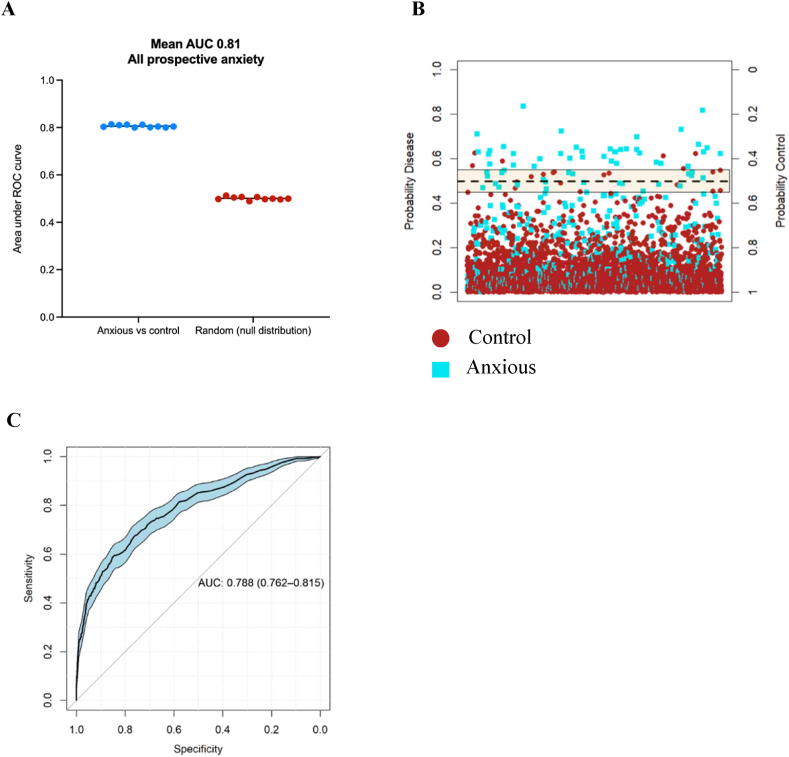
Anxiety disorder diagnosed any time after blood sampling was accurately predicted against unmatched control cases by random forest modelling when incorporating demographic, social, and biological factors. A) The random forest 10-fold cross validation accurately distinguished the prospectively diagnosed anxiety cohort against the unmatched control group (mean ROC AUC: 0.81), compared to the null distribution random forest model (mean ROC AUC: 0.5). B) Application of the trained random forest model to the independent final test data resulted in accurate prediction discrimination between prospectively diagnosed anxiety disorder (true anxious cases labelled blue) against control cases (true control cases labelled red). C) The ROC curve, summarising prediction quality at the independent testing stage, demonstrates the random forest model discriminated between all prospective anxiety disorder cases against controls with a ROC AUC of 0.79 (95 % CI 0.76 to 0.82).

The random forest algorithm classified any anxiety diagnosis after blood sampling (mean 9.5 years) with a mean cross-validated ROC AUC of 0.81, compared to the ROC AUC prediction of 0.50 from the null distribution model (Fig. 5A). The mean OOB error during cross-validation of the training set was approximately 0.28. The average accuracy, sensitivity, and specificity were 73 %, 68 %, and 79 % respectively. Applying the trained random forest model to the final independent test set (comprising 10 % of the data), resulted in a ROC AUC of 0.79 (95 % CI 0.76–0.82) (Fig. 5B and C). Relative importance of variables to the accuracy of the random forest algorithm is provided in Table 2 (see Supplementary Fig. S5 for ranked variable importance, and Supplementary Table S14 for further detail). Of note, neuroticism was again the most important driver of classification accuracy in the model, appearing as the most important predictor across the total 100 cross-validation models. Elevated inflammation and reduced red blood cell related counts were also significantly associated with prospective anxiety disorder (see Supplementary Table S14 for full variable comparisons, and Supplementary Table S13 for odds ratios).

Anxiety disorders diagnosed up to 5 years after and any time after blood sampling were predicted against lifetime anxiety free controls using solely blood biomarkers as predictors with a 10-fold cross validated ROC AUC of 0.68 and 0.65 respectively (see Supplementary Fig. S6 for independent final test results, see Supplementary Fig. S7–8 for ranked variable importance). Although these results do not indicate an accurate discrimination ability of the random forest, similar biomarkers driving the accuracy of the previous unmatched random forest analyses using psychosocial and biological features as predictors, also drove the accuracy of the unmatched random forest analysis, such as red blood cell related markers and inflammatory markers.

### 3.3 Neuroticism in anxiety and associated biomarkers

**Fig. 6:**
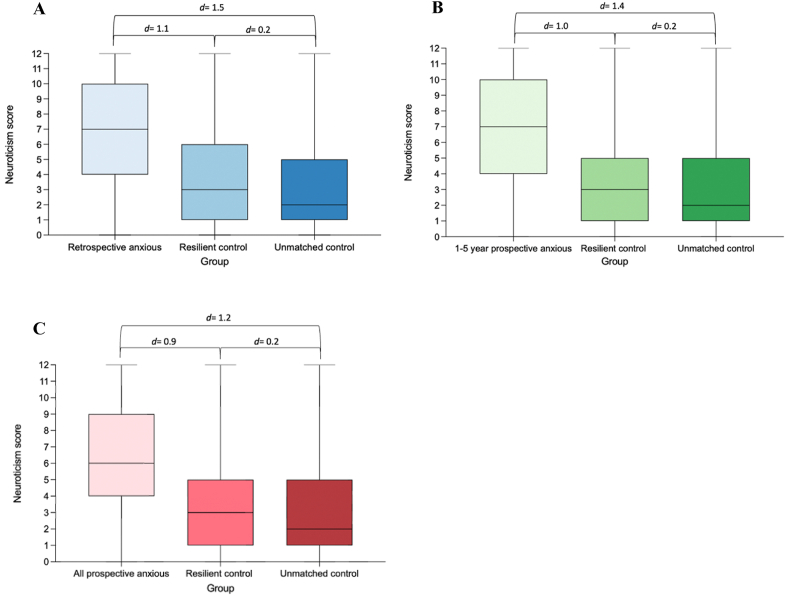
Neuroticism score is significantly higher in anxious individuals compared to control groups. Retrospective anxiety comparisons (shades of blue), 1–5-year prospective anxiety comparisons (shades of green), and all prospective anxiety comparisons (shades of red). A) Average neuroticism score across ‘retrospective anxious’ (n = 214), resilient control (n = 205), and unmatched control groups (n = 45,949). B) Average neuroticism score across ‘1-5-year prospective anxious’ (n = 627), resilient control (n = 617), and unmatched control groups (n = 45,949). C) Average neuroticism score across ‘all prospective anxious’ (n = 3773), resilient control (n = 3701), and unmatched control groups (n = 45,949). One-way ANOVA was performed to identify overall significant differences for the three main comparisons. Pairwise t-tests with Benjamini-Hochberg correction for multiple comparisons was used to compare average neuroticism score between groups, p < 0.05 across all comparisons. Cohen's d analysis performed to identify effect size.

Neuroticism was further explored due to its substantial relative importance in the predictive accuracy of future anxiety disorder. Average neuroticism scores were significantly higher (p = <0.01) in the anxious cohorts relative to their resilient matched control and the unmatched control group, determined using a one-way ANOVA and pairwise t-tests with an adjusted p-value using the Benjamini-Hochberg correction for multiple comparisons (Fig. 6). Cohen's d analysis also revealed a large effect size in the neuroticism score between anxious cohorts and their respective control groups.

**Fig. 7:**
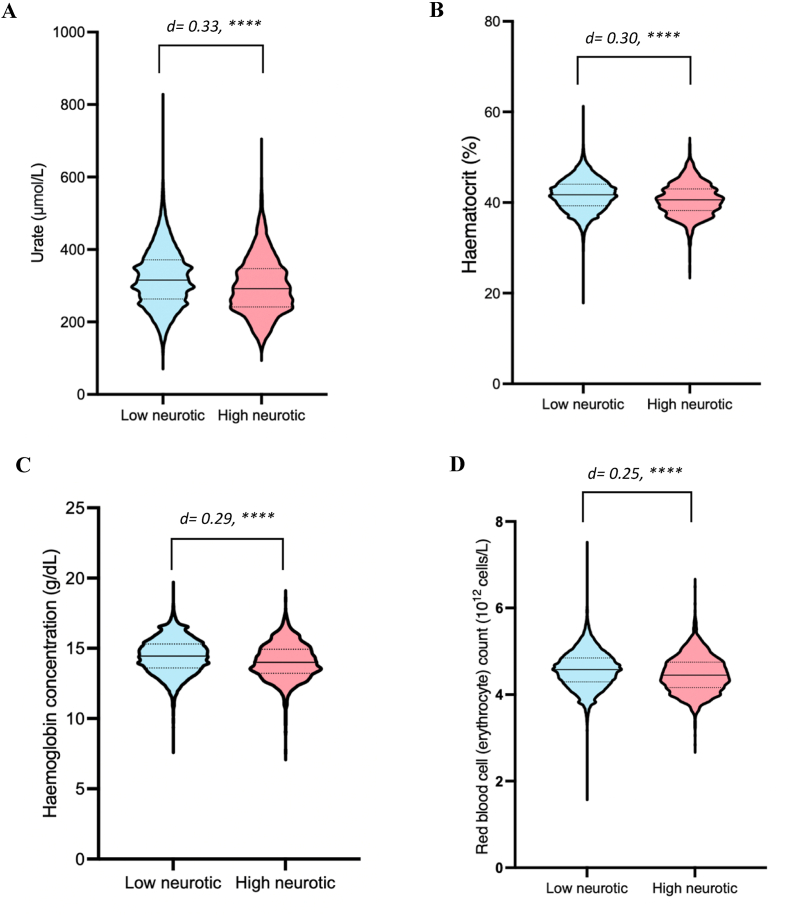
Urate and red blood cell related biomarkers are significantly lower in individuals with extremely high neuroticism. Low neurotic group (light blue), high neurotic group (red). A) Violin plots of blood urate concentration (μmol/L). B) Violin plots of blood haematocrit (%). C) Violin plots of haemoglobin concentration (g/dL). D) Violin plots of red blood cell (erythrocyte) count (10^12^ cells/L). Solid line through violin plots represents the median value, dashed lines represent the interquartile range. Unpaired two-tailed t-tests with Benjamini-Hochberg correction for multiple comparison to compare between groups, ∗∗∗∗ = p < 0.0001. Cohen's d effect size analysis performed.

A range of biomarker levels were significantly different between highly neurotic individuals (scoring 10–12, n = 1813) to low neurotic individuals (scoring 0–2, n = 23,898). Supplementary Table S15 provides details of the eight biomarkers that differed with a Cohen's d effect size greater than 0.2 or its inverse of −0.2, indicative of a small effect size. Urate differed the most between groups, with a Cohen's d value of 0.33 (95 % CI 0.28–0.39), and an approximately 6.7 % reduced concentration found in the high extreme (p < 0.0001) (Fig. 7A, Supplementary Table S15). Urate was also found to be an important driver of accuracy in the random forest prediction of future anxiety disorder, as seen in Results 4.2 (see Supplementary Table S13 for urate odds ratios in prospective anxious cases). Red blood cell related biomarkers including haematocrit (%), haemoglobin concentration, and red blood cell count, were also 2.4 %, 2.6 %, and 2.2 % lower in the high extreme compared to the low extreme (p < 0.0001 for all) (Fig. 7B–D, Supplementary Table S15).

**Fig. 8:**
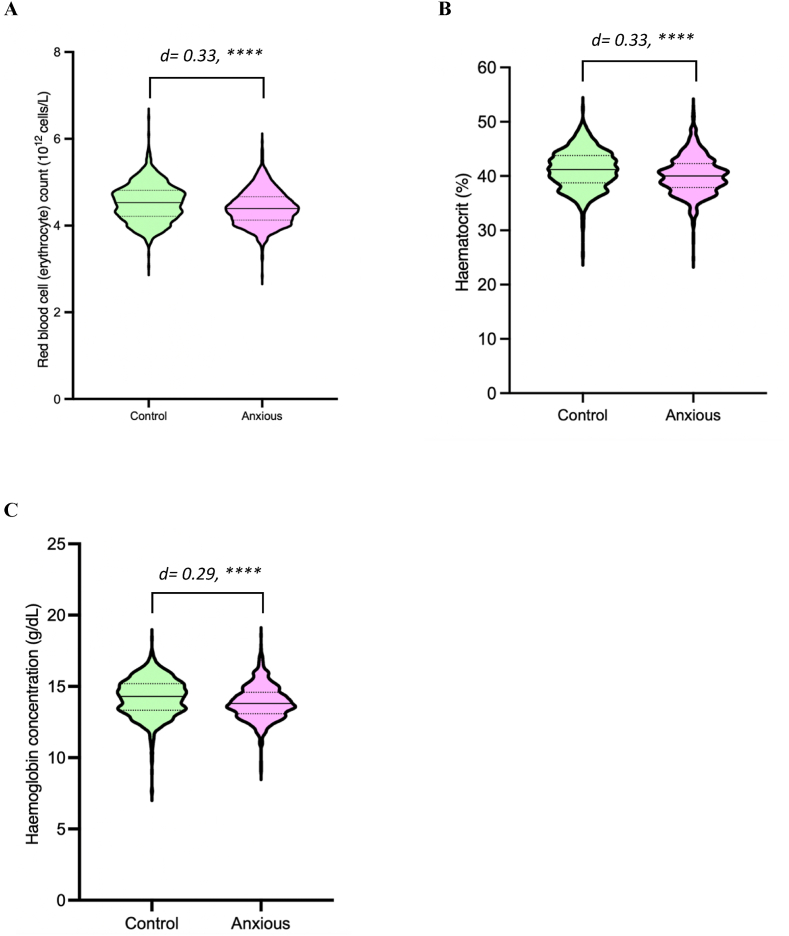
Red blood cell related biomarkers are significantly reduced in highly neurotic anxious individuals compared to highly neurotic lifetime anxiety free (control) individuals. Highly neurotic control (green), highly neurotic anxious (magenta). A) Violin plot of red blood cell (erythrocyte) count (10^12^ cells/L). B) Violin plot of haematocrit (%). C) Violin plot of haemoglobin concentration (g/dL). Solid line within violin plot represents the median, dashed lines represent the interquartile range. Unpaired two-tailed *t*-test comparison between groups, with Benjamini-Hochberg correction of p-values for multiple comparisons, ∗∗∗∗ = p < 0.0001. Cohen's d analysis performed for effect size determination.

Subsequently, blood-biomarker differences were assessed between highly neurotic individuals with anxiety disorder (n = 946) compared to highly neurotic individuals who are lifetime anxiety free (n = 867). Supplementary Table S16 provides further details regarding the average for the thirty-three biomarkers, across the highly neurotic and low neurotic groups, that had an effect size greater than 0.2 or the inverse −0.2, along with their reference ranges. Similar to previous analyses, red blood cell related biomarkers were significantly lower in the highly neurotic anxious individuals compared to the highly neurotic control group (Fig. 8, Supplementary Table S16). Comparing the anxious cohort to the control, red blood cell count was 2.7 % lower, haematocrit (%) was 2.6 % lower, and haemoglobin concentration was 2.4 % lower (p < 0.0001 across all comparisons). Additionally, 28 triglyceride and lipoprotein related biomarkers significantly differed between groups with an effect size above 0.2 (p= <0.01 across all comparisons). Further, GlycA, a marker of inflammation, was 2.5 % higher in the highly neurotic anxious group compared to the highly neurotic control (p < 0.0001).

### 3.4 Incorporation of trauma history improves the machine learning prediction of anxiety disorder to a ROC AUC of 0.85

**Fig. 9:**
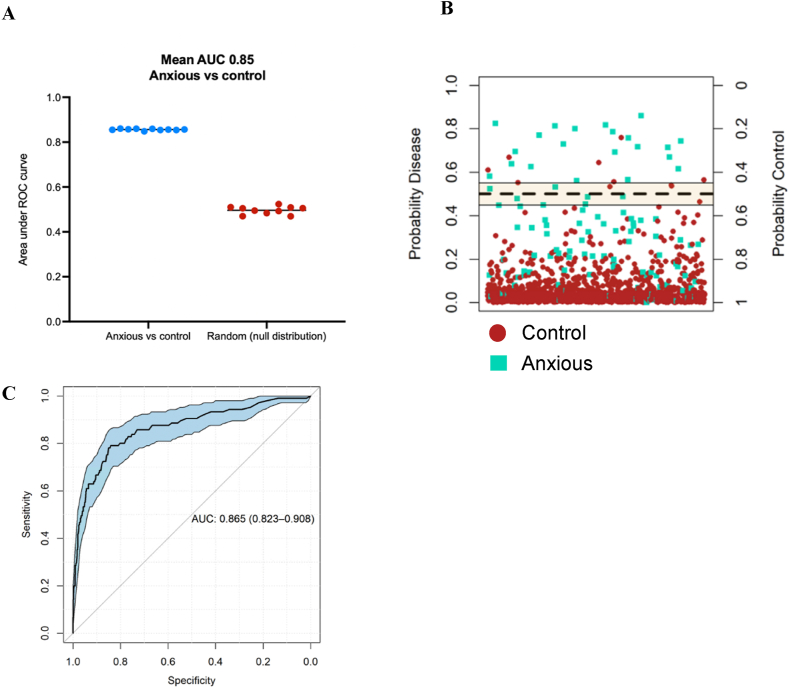
Anxiety disorder was accurately predicted against unmatched control cases when incorporating, demographic, social, biological factors, and trauma history. A) The random forest 10-fold cross validation accurately distinguished the prospectively diagnosed anxiety cohort against the unmatched control group (mean ROC AUC: 0.85), compared to the null distribution model (ROC AUC: 0.5). B) Application of the trained random forest model to the independent final test data resulted accurate prediction discrimination between individuals with a diagnosed anxiety disorder (true anxious cases labelled turquoise) against lifetime anxiety free control cases (true control cases labelled red). C) The ROC curve, summarising prediction quality at the independent testing stage, demonstrates that the model discriminated between anxious cases and controls with a ROC AUC of 0.87 (95 % CI 0.82–0.91).

**Fig. 10:**
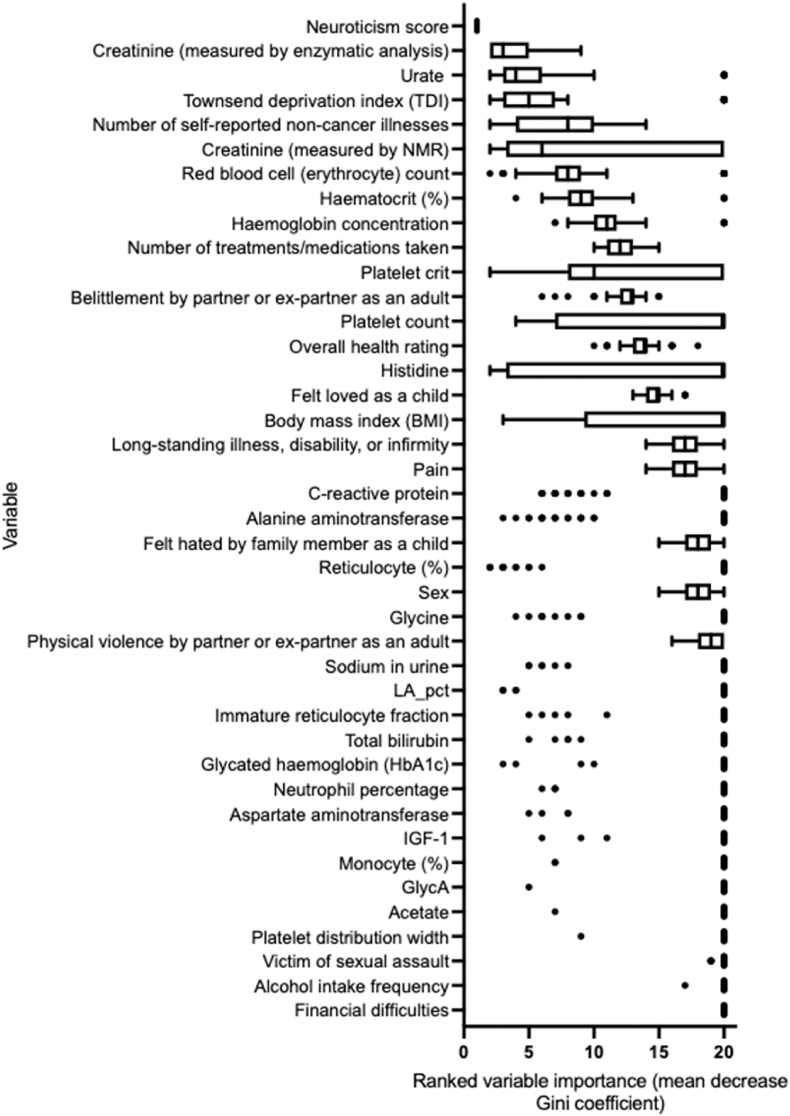
Features of trauma history rank amongst the top 10 % most important features driving the accuracy of random forest models distinguishing anxious cases against controls, when incorporating demographic, psychosocial, and blood biomarkers as predictors. Ranking of 1 reflects the most important predictor across all feature selected models during cross validation, ranking of 20 reflects the lowest ranked predictors in the feature selected models.

To assess whether incorporation of trauma history improves the machine learning prediction of anxiety disorder, a cohort of individuals that provided answers to 11 trauma history questions were analysed (questions listed in Supplementary Table S11). Trauma history answers were used as predictor variables, along with all demographic and biomarker factors that had been used in the random forest model discussed in Results 4.2. The random forest model classified individuals with anxiety disorder at a mean cross-validated ROC AUC value of 0.85, compared to the null distribution ROC AUC of 0.50; a 2.4 % improvement from the model only considering demographic and biomarker variables (Fig. 9A). Mean OOB error across the 10-fold cross validation was 0.23. The mean cross-validated accuracy, sensitivity, and specificity were 78 %, 74 %, and 82 % respectively. Application of the trained random forest model to the final independent test set (comprising 10 % of the data) resulted in a similarly high ROC AUC value of 0.87 (95 % CI 0.82–0.91) (Fig. 9B and C). The ranked top 10 % most important features contributing towards the accuracy of the model (Fig. 10), along with a comparison of the difference in traumatic history between anxious and control cohorts (Supplementary Table S10), demonstrate the importance of trauma history in predicting anxiety disorder. Whilst sex was included in a proportion of the models as a key feature for anxiety prediction, stratifying the model by males and females did not improve predictive capacity of either model, and the top predictive features were similar between males and females (Fig. S11 and S12).

### 3.5 Biomarkers associated with traumatic history

Comparing biomarkers between individuals that had experienced a low amount of trauma (scoring ≤11 across 11 trauma history questions, n = 3689) and those who have experienced a high amount of trauma (scoring ≥23, n = 204) revealed 71 biomarkers with an effect size greater than 0.2, or −0.2 in the inverse direction (see Supplementary Table S17 for full details). Reticulocyte (%), high light scatter reticulocyte (%), and high light scatter reticulocyte count, were all found to be significantly elevated in individuals with a high history of trauma compared to those with a low history of trauma (p < 0.05 for all, Cohen's d > 0.3 for all) (Table 3).

**Table 3:** Top 3 biomarkers with the greatest effect size difference between individuals with a low amount of trauma compared to those with a higher amount of trauma.

	Average low trauma (n = 3689)	Average high trauma (n = 204)	Cohen's d effect size [95 % CI]	Adjusted p-value
Reticulocyte (%)	1.29	1.55	0.33 [0.19 to 0.47]	0.002
High light scatter reticulocyte (%)	0.37	0.43	0.32 [0.18 to 0.46]	0.002
High light scatter reticulocyte count	0.017	0.020	0.31 [0.17 to 0.45]	0.002

Note: Unpaired two-tailed *t*-test comparisons with an adjusted p-value using the Benjamini-Hochberg correction for multiple comparisons. Effect size calculated using Cohen's d analysis.

Finally, blood biomarkers between individuals with an anxiety disorder diagnosis that have undergone a high amount of trauma (n = 105) were compared to control cases (n = 99) who have also experienced high trauma. Whilst twenty-two biomarkers had an effect size greater than 0.2 or it's inverse, as shown in Supplementary Table S18, only two biomarkers were significantly different when adjusting the p-value using the Benjamini-Hochberg correction (Table 4). Haemoglobin concentration was found to be 6 % lower in the anxious cohort compared to control, with haematocrit (%) 5 % lower (p < 0.05 for both).

**Table 4:** Biomarkers that significantly differed between individuals with anxiety disorder against lifetime anxiety free controls, who have all experienced a highly traumatic history.

	Average control (n = 99)	Average anxious (n = 105)	Cohen's d effect size [95 % CI]	Adjusted p-value
Haemoglobin concentration	14.4	13.6	−0.62 [-0.90 to −0.34]	0.01
Haematocrit (%)	41.6	39.6	−0.59 [-0.87 to −0.30]	0.01

Note: Unpaired two-tailed *t*-test comparisons with an adjusted p-value using the Benjamini-Hochberg correction for multiple comparisons. Effect size calculated using Cohen's d analysis.

## 4 Discussion

In this study, we employed random forest methods to identify psychosocial and biological risk factors conferring susceptibility or resilience to anxiety disorder. We first discovered that the machine learning (ML) could not accurately distinguish anxiety disorders against ‘resilient’ controls, when a wide range of covariates (demographics and life stressors) were controlled for, using only blood biomarkers used as predictors. However, random forest models did accurately predict future anxiety disorder onset using a combination of biomarker, demographic, and social factors as predictors. Neuroticism was identified as a key driver of model accuracy in predicting future disorder onset, along with biomarkers related to anaemia, inflammation, and uric acid. Importantly, ML models assessing the unmatched prospective anxious cohort against lifetime anxiety free controls, using only biomarkers as predictors, also revealed the same biomarkers to be within the top 10 % most important predictors. While we did not include depression-specific variables, key psychosocial predictors such as neuroticism and trauma also capture shared risk with depression, enabling broader relevance while maintaining a focus on anxiety-specific outcomes.

### 4.1 Important predictors of future anxiety disorder diagnosis

Although there are known sociodemographic risk factors for anxiety disorder, most individuals who experience these known risk factors will not develop an anxiety disorder. By understanding the pathways in place enabling the ability to remain ‘resilient’ in the face of stress may reveal intervention points to promote a resilient phenotype in anxious individuals (Kalisch et al., 2017). The ML models were unable to accurately distinguish between anxious and resilient cases using solely blood biomarkers as predictors, demonstrating that intrinsic biomarkers governing anxiety disorder susceptibility or resilience also depend on sociodemographic factors.

Integrating sociodemographic features (demographics and life stressors) alongside blood biomarkers and neuroticism score resulted in the ML models accurately distinguishing between individuals who would develop an anxiety disorder after blood sampling against lifetime anxiety free control cases (ROC AUC 0.83). Neuroticism scores were found to be significantly higher in anxious cohorts compared to the resilient and unmatched control groups. Considering neuroticism and trauma history are well known risk factors for anxiety disorder in the field of psychology, this demonstrates the validity of our approach. Whilst there is an established link between anxiety, high neuroticism, and trauma, there is little translation of this knowledge into identifying potential pathways for intervention (Ormel et al., 2013).

A range of biomarkers appeared as important predictors in the unmatched random forest analysis classifying future anxiety disorder against controls, including markers of anaemia and inflammation.

### 4.2 Anaemia as a risk factor for future anxiety disorder

Haemoglobin concentration, red blood cell count, and haematocrit (%) were significantly lower in anxious cases compared to controls. A separate study applying a variety of machine learning approaches to assess relative blood-based biomarker importance in anxiety disorder identified similar findings retrospectively (Sharma and Verbeke, 2021). Our study takes this further by demonstrating the incorporation of these factors within random forest enables prediction accuracy in the classification of future anxiety disorder development. A study by Shafiee et al. also found reduced RBCs in severely anxious males compared to controls; the odds ratio remained significant even after adjusting for potential confounders (Shafiee et al., 2017). Further work should aim to assess the relationship between these markers with the severity and sex-specificity of future anxiety outcomes.

Reduced red blood cell related counts were also significant in individuals with anxiety disorder that have high neuroticism and high trauma history compared to controls with the same levels of trauma history and neuroticism score (Table 4). The role of stressors, such as traumatic events and high neuroticism, in red blood cell related markers would be an interesting area for further research. Causal mediation analysis would provide greater insight into these relationships, by providing further information into which biomarkers (such as haemoglobin concentration or reticulocyte counts) may be mediating the effect of exposures (such as stress history and neuroticism) on anxiety disorder outcomes.

While acute stress is generally associated with transient increases in erythropoiesis through catecholamine and cortisol release, chronic psychological stress may impair red blood cell production over time via sustained inflammation, altered iron metabolism, and dysregulation of erythropoietin signalling. Chronic low-grade inflammation that may be observed in anxious individuals (who also tend to have more comorbid illnesses and take more medications) can lead to functional iron deficiency and suppression of erythropoiesis, contributing to mild anaemia. Conversely, reduced oxygen-carrying capacity and associated fatigue or cognitive impairment may exacerbate anxious symptomatology, creating a bidirectional relationship. Our findings suggest that lower RBC indices may reflect both a consequence of chronic stress and a contributing factor to vulnerability for anxiety disorder.

### 4.3 Reduced uric acid in anxious and highly neurotic individuals

Urate (uric acid), the end-product of purine metabolism, was another important biomarker driving the accuracy of the random forest models in classifying future anxiety disorder. Uric acid was significantly lower in the prospective anxious cohort compared to control. These results align with an emerging literature identifying an association between serum uric acid (SUA) levels with neuroticism and anxiety. Qian et al., were the first to identify, using Mendelian randomisation approaches, that single nucleotide polymorphisms (SNPs) related to genetic predictors of uric acid, specifically the SCL2A9 gene, have significant associations with neuroticism; their results suggesting that increased SUA levels associate with reduced neuroticism levels (Qian et al., 2021). In addition, Black et al., also identified a dose-dependent negative relationship between plasma uric acid levels and anxiety symptom severity (p for linear trend 0.032) (Black et al., 2018). Our results align with the general literature that lower serum uric acid levels appear to associate with an anxious and highly neurotic state. Further work should assess whether increasing uric acid levels in anxious individuals improves emotional resilience to stress related tasks and whether this may be an effective treatment avenue; specifically, identifying whether uric acid level plays a causal role in emotional regulation or is a by-product.

### 4.4 Elevated inflammation in anxious individuals

Inflammatory biomarkers, including increased blood C-reactive protein (CRP) and GlycA, contributed significantly to the prediction of anxiety compared to controls. Our results of an association between CRP and anxiety disorder aligns with a relatively large body of literature connecting systemic inflammation to psychiatric disorders. Most research however has a focus on depression, schizophrenia, and bipolar disorder, with comparatively far fewer work focusing on anxiety disorder (Costello et al., 2019). We are the first to report a relationship between GlycA and anxiety disorder, while positive associations between GlycA levels and depression severity have been previously demonstrated (Brunoni et al., 2020; Huckvale et al., 2020).

It has also been identified that individuals who experienced childhood trauma exhibit elevated inflammation, with some proposing that stress becomes biologically embedded as a result of inflammatory processes in childhood (Danese et al., 2011). Rodent studies have demonstrated administration of proinflammatory stress at the peripheral level within 5 days of post-natal life leads to significant increases in anxiety by adolescence (Broshevitskaya et al., 2021). A meta-analysis assessing the relationship between childhood trauma and inflammation found adults exposed to childhood trauma had significantly higher CRP, interleukin-6 (IL-6) and tumour necrosis factor alpha (TNF-α) levels (Baumeister et al., 2016). Although in our work, we group a range of childhood and adult traumatic experiences, we do not find a significant difference in CRP levels when comparing individuals that had experienced high trauma compared to low trauma history, however significantly elevated GlycA levels were identified in those with high trauma (d = 0.27 [95 % CI 0.13 to 0.41]).

There are a variety of mechanisms through which peripheral inflammation, as evidenced by elevated CRP and GlycA levels, may alter brain activity and have implications for the expression of anxious behaviour. Peripheral inflammation can trigger de novo synthesis of proinflammatory within the CNS via peripheral vagal nerve afferents or transduction at the level of the blood-brain barrier (Sun et al., 2022). For example, the peripheral administration of inflammatory cytokines has been shown to induce symptoms of depression and anxiety, which consistently coincides with the activation of anxiety relevant brain regions, such as the amygdala, insula, and anterior cingulate cortex, as revealed by neuroimaging studies (Felger and Treadway, 2017; Goldsmith et al., 2023). In systemic inflammatory disease it has been demonstrated that microglial cells, activated by cytokine signalling from the periphery, release the monocyte chemoattractant protein-1 (MCP-1), a chemokine which activates monocyte/macrophage trafficking to the perivascular and meningeal spaces in the brain (D'Mello et al., 2009). These processes have been associated with anxiety-like behaviour in rodents, with post-mortem studies finding evidence of activated peripheral macrophages within perivascular spaces at the dorsal anterior cingulate cortex (ACC) in suicide victims (Torres-Platas et al., 2014; Wohleb et al., 2014). Disordered activity of the ACC can result in hyperactivity of the amygdala, an important fear processing region, which can have implications for anxiety (McClure et al., 2007). The elevated CRP and GlycA in anxious individuals within this work may reflect a role of inflammatory processes in mediating anxiety symptoms.

### 4.5 Limitations and future directions

The UK Biobank is largely cross-sectional in nature, and future work in this area would benefit from longitudinal blood sampling, to assess changes in blood biomarker concentrations over time and map fluctuations with treatment response and changes in symptom severity. In this study, it was not possible to stratify based on the specific sub-type of anxiety disorder diagnosis, as approximately 64 % of anxiety cases were unspecified anxiety disorders; however, stratification may enable ML models to better account for the heterogeneity of anxiety disorders. It was also not possible to identify whether individuals diagnosed with an anxiety disorder after blood sampling had self-reported anxiety symptoms prior to diagnosis. This may have impacted the predictive capacity of the biomarkers identified in this work. However, it is also important to recognise the transition from self-reporting anxiety to obtaining a clinical diagnosis is often a reflection of a change in severity or quality of life due to the disorder. Finally, there is selection bias within the UK Biobank, as the cohort in general is older, healthier, of European ancestry, with more women, and of a higher socioeconomic status than the general UK population (Fry et al., 2017).

Nearly half (48.7 %) of individuals in the anxious cohort had a history of comorbid depression. While excluding these individuals might have provided a more narrowly defined anxiety phenotype, doing so would have substantially reduced the sample size and limited the clinical relevance of our findings. In contrast, the control cohort—rigorously defined to exclude lifetime anxiety—contained very few individuals with depression (∼1 %), reducing the risk of confounding. Furthermore, in our prior work focused on depression (Radford-Smith et al., 2023), we identified distinct metabolic signatures associated with depressive disorders, including different top-ranking biomarkers such as lactate and pyruvate. The divergence in predictive variables between the current and previous models supports the specificity of the anxiety-related findings despite shared psychosocial features such as neuroticism. While future analyses comparing ‘pure’ anxiety and ‘pure’ depression groups may be informative, our current approach reflects the high rate of comorbidity seen in clinical practice and ensures greater translational applicability of the results.

To facilitate clinical implementation, it is encouraging that the most predictive features in our models—such as neuroticism score, number of illnesses and medications, and social deprivation—can be obtained through brief self-report questionnaires or routine medical records. The key biochemical predictors, including serum creatinine, red blood cell count, and urate, are derived from standard haematology and biochemistry panels already processed in most hospital laboratories. This supports the feasibility of embedding a simplified risk algorithm into primary care or mental health triage settings. Future work should focus on validating a reduced composite panel in an independent cohort, assessing both predictive performance and ease of integration into clinical workflows.

As we have identified elevated CRP and GlycA to be predictive of future anxiety disorder diagnosis, further research is warranted to assess whether the prophylactic use of anti-inflammatory drugs is associated with a reduced risk of anxiety disorder onset. It was shown that the usage of non-steroidal anti-inflammatory drugs prior to a cancer diagnosis was associated with a reduced risk of developing depression, anxiety, and other stress related disorders in the year after diagnosis (HR 0.88, 95 % CI 0.81–0.97) (Hu et al., 2020). However, by grouping depression and anxiety disorders as one disease entity, it is not possible to see the direct relationship between reducing inflammation and anxiety symptoms. Further research assessing the preventative potential of anti-inflammatory drugs against anxiety disorder, in individuals with evidence of low-grade inflammation, is warranted.

## 5 Conclusion

We have taken an untargeted approach using machine learning on UK Biobank participants to identify psychosocial (demographic, life stressors, neuroticism, and trauma history), blood biomarkers and urinary biomarkers that confer susceptibility or resilience to anxiety disorder. We found higher neuroticism scores to be the most important driver in accurately predicting those that would develop anxiety disorder after a blood sample. Trauma history was also identified to be an important psychosocial predictor, in alignment with current literature. Surprisingly, we found that biomarkers indicative of elevated risk of anaemia, alongside elevated inflammation and reduced uric acid levels to be some of the most important biomarkers predictive of future anxiety disorder. Whilst there is research identifying a link between anxiety disorder and elevated inflammation, this study is one of the first to demonstrate the potential of CRP and GlycA as predictive biomarkers. Additional research assessing the relationship between biomarkers indicative of low iron, vitamin B12, or folic acid, as an early sign of anxiety disorder are also justified based on our findings. Finally, further investigation into the potential of elevating uric acid to reduce the risk of anxiety disorder onset is also recommended based on our results. These findings expand the current literature on predictive biomarkers for anxiety disorder, acting as possible new intervention points for prophylactic treatment.

## CRediT authorship contribution statement

**Annabel Smith:** Writing – original draft, Investigation, Formal analysis. **Jack J. Miller:** Methodology, Formal analysis. **Daniel C. Anthony:** Writing – review & editing, Conceptualization. **Daniel E. Radford-Smith:** Writing – review & editing, Supervision, Methodology, Formal analysis, Conceptualization.

## Declaration of competing interest

The authors declare that they have no known competing financial interests or personal relationships that could have appeared to influence the work reported in this paper.

## Data Availability

The authors do not have permission to share data.
