# Supplementary material for: Machine learning-based prediction of anxiety disorders using blood metabolite and social trait data from the UK Biobank
Source: Brain Behav Immun Health. 2025 May 8;46:101010. doi: 10.1016/j.bbih.2025.101010 (PMC12140939; doi:10.1016/j.bbih.2025.101010)
Supplement: Multimedia component 1 [file mmc1.docx]

**Supplementary methods**

**Biomarker Panel**

| WBC count | GlycA | M_HDL_L | M_HDL_FC_pct |
| --- | --- | --- | --- |
| RBC count | XXL_VLDL_P | S_HDL_L | M_HDL_C_pct |
| Haemoglobin concentration | XXL_VLDL_PL | VLDL_CE | M_HDL_PL_pct |
| Haematocrit % | XXL_VLDL_CE | VLDL_FC | M_HDL_TG_pct |
| MCV | XXL_VLDL_FC | VLDL_C | S_HDL_CE_pct |
| MCH | XXL_VLDL_TG | VLDL_PL | S_HDL_FC_pct |
| MCH concentration | XL_VLDL_P | VLDL_TG | S_HDL_C_pct |
| RBC distribution width | XL_VLDL_PL | VLDL_L | S_HDL_PL_pct |
| Platelet count | XL_VLDL_CE | VLDL_P | S_HDL_TG_pct |
| Platelet crit | XL_VLDL_FC | LDL_CE | Omega_3_pct |
| Mean platelet volume | XL_VLDL_TG | LDL_FC | Omega_6_pct |
| Platelet distrbution width | L_VLDL_P | LDL_C | LA_pct |
| Lymphocyte count | L_VLDL_PL | LDL_PL | MUFA_pct |
| Monocyte count | L_VLDL_CE | LDL_TG | PUFA_pct |
| Neutrophil count | L_VLDL_FC | LDL_L | SFA_pct |
| Eosinophil count | L_VLDL_TG | LDL_P | DHA_pct |
| Basophil count | M_VLDL_P | HDL_CE | Omega_6_by_Omega_3 |
| Lymphocyte % | M_VLDL_PL | HDL_FC | ApoB_by_ApoA1 |
| Monocyte % | M_VLDL_CE | HDL_C | PUFA_by_MUFA |
| Neutrophil % | M_VLDL_FC | HDL_PL | TG_by_PG |
| Eosinophil % | M_VLDL_TG | HDL_TG | XXL_VLDL_FC_pct_C |
| Basophil % | S_VLDL_P | HDL_L | XL_VLDL_FC_pct_C |
| Reticulocyte % | S_VLDL_PL | HDL_P | L_VLDL_FC_pct_C |
| Reticulocyte count | S_VLDL_CE | Total_CE | M_VLDL_FC_pct_C |
| Mean reticulocyte volume | S_VLDL_FC | Total_FC | S_VLDL_FC_pct_C |
| Mean sphered cell volume | S_VLDL_TG | Total_C | XS_VLDL_FC_pct_C |
| Immature reticulocyte fraction | XS_VLDL_P | Total_PL | L_LDL_FC_pct_C |
| High light scatter reticulocyte % | XS_VLDL_PL | Total_TG | M_LDL_FC_pct_C |
| High light scatter reticulocyte count | XS_VLDL_CE | Total_L | S_LDL_FC_pct_C |
| Microalbumin in urine | XS_VLDL_FC | Total_P | IDL_FC_pct_C |
| Urine creatinine | XS_VLDL_TG | PUFA | XL_HDL_FC_pct_C |
| Urine potassium | IDL_P | Total_FA | L_HDL_FC_pct_C |
| Urine sodium | IDL_PL | Total_BCAA | M_HDL_FC_pct_C |
| Albumin | IDL_CE | non_HDL_C | S_HDL_FC_pct_C |
| Alkaline phosphatase | IDL_FC | Remnant_C | XXL_VLDL_CE_pct_C |
| Alanine aminotransferase | IDL_TG | XXL_VLDL_CE_pct | XL_VLDL_CE_pct_C |
| ApoA | L_LDL_P | XXL_VLDL_FC_pct | L_VLDL_CE_pct_C |
| ApoB | L_LDL_PL | XXL_VLDL_C_pct | M_VLDL_CE_pct_C |
| Aspartate aminotransferase | L_LDL_CE | XXL_VLDL_PL_pct | S_VLDL_CE_pct_C |
| Direct bilirubin | L_LDL_FC | XXL_VLDL_TG_pct | XS_VLDL_CE_pct_C |
| Urea | L_LDL_TG | XL_VLDL_CE_pct | L_LDL_CE_pct_C |
| Calcium | M_LDL_P | XL_VLDL_FC_pct | M_LDL_CE_pct_C |
| Cholesterol | M_LDL_PL | XL_VLDL_C_pct | S_LDL_CE_pct_C |
| Creatinine | M_LDL_CE | XL_VLDL_PL_pct | IDL_CE_pct_C |
| CRP | M_LDL_FC | XL_VLDL_TG_pct | XL_HDL_CE_pct_C |
| Cystatin C | M_LDL_TG | L_VLDL_CE_pct | L_HDL_CE_pct_C |
| Gamma glutamyltransferase | S_LDL_P | L_VLDL_FC_pct | M_HDL_CE_pct_C |
| Glucose | S_LDL_PL | L_VLDL_C_pct | S_HDL_CE_pct_C |
| HbA1c | S_LDL_CE | L_VLDL_PL_pct | XXL_VLDL_FC_by_CE |
| HDL cholesterol | S_LDL_FC | L_VLDL_TG_pct | XL_VLDL_FC_by_CE |
| IGF-1 | S_LDL_TG | M_VLDL_CE_pct | L_VLDL_FC_by_CE |
| LDL direct | XL_HDL_P | M_VLDL_FC_pct | M_VLDL_FC_by_CE |
| Lipoprotein A | XL_HDL_PL | M_VLDL_C_pct | S_VLDL_FC_by_CE |
| Phosphate | XL_HDL_CE | M_VLDL_PL_pct | XS_VLDL_FC_by_CE |
| SHBG | XL_HDL_FC | M_VLDL_TG_pct | L_LDL_FC_by_CE |
| Total bilirubin | XL_HDL_TG | S_VLDL_CE_pct | M_LDL_FC_by_CE |
| Testosterone | L_HDL_P | S_VLDL_FC_pct | S_LDL_FC_by_CE |
| Total protein | L_HDL_PL | S_VLDL_C_pct | IDL_FC_by_CE |
| Triglycerides | L_HDL_CE | S_VLDL_PL_pct | XL_HDL_FC_by_CE |
| Urate | L_HDL_FC | S_VLDL_TG_pct | L_HDL_FC_by_CE |
| Vitamin D | L_HDL_TG | XS_VLDL_CE_pct | M_HDL_FC_by_CE |
| Clinical_LDL_C | M_HDL_P | XS_VLDL_FC_pct | S_HDL_FC_by_CE |
| VLDL_size | M_HDL_PL | XS_VLDL_C_pct | VLDL_CE_pct |
| LDL_size | M_HDL_CE | XS_VLDL_PL_pct | VLDL_FC_pct |
| HDL_size | M_HDL_FC | XS_VLDL_TG_pct | VLDL_C_pct |
| Phosphoglyc | M_HDL_TG | L_LDL_CE_pct | VLDL_PL_pct |
| Cholines | S_HDL_P | L_LDL_FC_pct | VLDL_TG_pct |
| Phosphatidylc | S_HDL_PL | L_LDL_C_pct | LDL_CE_pct |
| Sphingomyelins | S_HDL_CE | L_LDL_PL_pct | LDL_FC_pct |
| ApoB | S_HDL_FC | L_LDL_TG_pct | LDL_C_pct |
| ApoA1 | S_HDL_TG | M_LDL_CE_pct | LDL_PL_pct |
| Unsaturation | XXL_VLDL_C | M_LDL_FC_pct | LDL_TG_pct |
| Omega_3 | XL_VLDL_C | M_LDL_C_pct | HDL_CE_pct |
| Omega_6 | L_VLDL_C | M_LDL_PL_pct | HDL_FC_pct |
| MUFA | M_VLDL_C | M_LDL_TG_pct | HDL_C_pct |
| SFA | S_VLDL_C | S_LDL_CE_pct | HDL_PL_pct |
| LA | XS_VLDL_C | S_LDL_FC_pct | HDL_TG_pct |
| DHA | IDL_C | S_LDL_C_pct | VLDL_FC_pct_C |
| Ala | L_LDL_C | S_LDL_PL_pct | LDL_FC_pct_C |
| Gln | M_LDL_C | S_LDL_TG_pct | HDL_FC_pct_C |
| Gly | S_LDL_C | IDL_CE_pct | VLDL_CE_pct_C |
| His | XL_HDL_C | IDL_FC_pct | LDL_CE_pct_C |
| Ile | L_HDL_C | IDL_C_pct | HDL_CE_pct_C |
| Leu | M_HDL_C | IDL_PL_pct | VLDL_FC_by_CE |
| Val | S_HDL_C | IDL_TG_pct | LDL_FC_by_CE |
| Phe | XXL_VLDL_L | XL_HDL_CE_pct | HDL_FC_by_CE |
| Tyr | XL_VLDL_L | XL_HDL_FC_pct | Total_CE_pct |
| Glucose | L_VLDL_L | XL_HDL_C_pct | Total_FC_pct |
| Lactate | M_VLDL_L | XL_HDL_PL_pct | Total_C_pct |
| Pyruvate | S_VLDL_L | XL_HDL_TG_pct | Total_PL_pct |
| Citrate | XS_VLDL_L | L_HDL_CE_pct | Total_TG_pct |
| bOHbutyrate | IDL_L | L_HDL_FC_pct | Total_FC_pct_C |
| Acetate | L_LDL_L | L_HDL_C_pct | Total_CE_pct_C |
| Acetoacetate | M_LDL_L | L_HDL_PL_pct | Total_FC_by_CE |
| Acetone | S_LDL_L | L_HDL_TG_pct | Omega_3_pct_PUFA |
| Creatinine | XL_HDL_L | M_HDL_CE_pct | Omega_6_pct_PUFA |
| Albumin | L_HDL_L |  |  |

UK biobank haematology data was collected from four Beckman Coulter LH750 instruments. Venous blood was collected in 4ml EDTA (Ethylenediaminetetraacetic acid) vacutainers for the (circa) 500,000 participants of the UK Biobank baseline cohort and run typically within 24 hours of blood being drawn. For further information, refer to the relevant UK Biobank showcase resource (<https://biobank.ndph.ox.ac.uk/showcase/refer.cgi?id=1453>).

Serum biomarker data were analysed using 10 immunoassay analysers (6x DiaSorin Liaison XL & 4x Beckman Coulter DXI 800 and 4 clinical chemistry analysers (2x Beckman Coulter AU5800 & 2x Siemens Advia 1800). For further details on quality assurance, refer to the relevant UK Biobank showcase resource (<https://biobank.ndph.ox.ac.uk/showcase/refer.cgi?id=1227>).

Metabolic biomarkers were quantified from EDTA plasma using Nightingale Health’s high-throughput nuclear magnetic resonance (NMR) platform, which has been CE-marked and accredited to ISO 13485 standards for clinical use. The platform measures 249 biomarkers, including lipids, amino acids, ketones, glycolysis-related metabolites, and detailed lipoprotein subclass profiles, using 500 MHz NMR spectroscopy. All analyses were performed in one of eight 500 MHz spectrometers. For further details on quality assurance, refer to the relevant UK Biobank showcase resource (<https://biobank.ndph.ox.ac.uk/showcase/refer.cgi?id=3000>).

**Random Forest**

Random forest data processing

Prior to random forest analysis, predictor variables where over 5% of individuals had missing data were excluded, as were individuals with over 10% of predictor variables missing. Subsequently, the “missRanger” v.2.2.1 R package was used to intelligently impute remaining missing values (Mayer, 2023)., preserving variance across predictor variables. Imputation was based on the class of individuals (anxious or control), with the ‘pmm.k’ argument set to a positive number of 3 to ensure imputations are similar in structure to the pre-existing data, and ‘num.trees’ (the number of random forest trees used) set to 10 to reduce computational time.

### Feature selection detail

Feature selection was applied during the 10-fold cross-validation of the random forest models (Fig. 2). With the matched cohorts, initial ‘dummy’ random forests were developed at each fold of cross validation, assessing all possible predictors in the dataset. Then, a feature selection stage was applied, whereby the top 5% most important features to the accuracy of each ‘dummy’ random forest model were used as the sole predictors for the feature selected models. The trained feature selected random forest model was then utilised for the final independent test. The exact same process was followed with the ‘1-5-year prospective anxious’ unmatched cohort analysis, where the top 5% most important features were selected; those features, were then used to train the random forest models in the ‘all prospective anxious’ unmatched cohort analysis, with no further feature selection applied.

**Neuroticism**

### Identifying biomarkers associated with neuroticism

The average neuroticism score across each anxious group, their resilient matched control, and the unmatched control were calculated. Overall significance was determined using the one-way ANOVA test. Inter-group differences were assessed using pairwise t-tests with an adjusted p-value, using the Benjamini-Hochberg method, to correct for multiple comparisons.

The blood biomarkers of all individuals with low neuroticism (score: 0-1, n= 23,898) were compared to all with high neuroticism (score: 10-12, n= 1,813) using t-tests, Cohen’s d effect size, and ROC AUC values. There is a lack of literature providing a boundary regarding ‘high’ and ‘low’ neuroticism relative to the EPQ-R scale used in the UK Biobank, therefore these boundaries are set based on extreme phenotypes. Subsequently, highly neurotic individuals with anxiety (n= 867) were compared to highly neurotic control individuals (n= 946), with the same statistical tests.

**History of trauma**

### Assessing the incorporation of trauma history in the predictive accuracy of the machine learning classification of anxiety disorder

A reduced cohort of UK Biobank participants (n= 158,298) provided information regarding their history of traumatic events, such as child abuse or witnessing a violent death as an adult (see Supplementary Table S10 for details, including chi-squared test results comparing trauma history experience between lifetime anxiety free controls and anxious individuals). Trauma history information was used in combination with the psychosocial (demographic, life stressors, and neuroticism score) and biological factors applied in the unmatched random forest analysis (discussed in Methods 3.2), to evaluate whether incorporation of traumatic history improves predictive accuracy. Due to the reduced number of anxious individuals who provided answers to the questionnaires (n= 996), anxious cases were not subdivided based on time of diagnosis. The full anxious cohort (n= 996) was compared to all control cases that matched the general exclusion criteria and answered the trauma questionnaire (n= 10,939). The same random forest analysis procedure as discussed in Methods 3.3.1 was applied. This included the cross-validation assessment of models on the training dataset, with class size matching, followed with an independent random forest analysis on the remaining independent test data (comprising 10% of the dataset) without class size matching.

### Identifying biomarkers associated with traumatic history

The blood biomarker composition of individuals that had experienced a high amount of trauma were compared to those who have experienced little to no trauma. The trauma history answers provided in the UK Biobank were converted to numerical form, such that higher total scores across the questions corresponded to a high amount of trauma (see Supplementary Table S11 for numerical coding). Individuals with a trauma score above 23 were classified as the ‘high’ trauma cohort (n= 204), with individuals at a trauma score of 11 or below classified as the ‘low’ trauma cohort (n= 3,689). There is no literature grouping trauma history from the UK Biobank into ‘high’ or ‘low’ boundaries, therefore similarly to the neuroticism cut offs, this study sets the boundaries at the extreme ends. The 385 blood biomarkers were compared using t-tests, effect sizes determined by Cohen’s d, and ROC AUC values across groups. Further, control cases who had experienced a high amount of trauma (n= 99) were compared to anxious cases who also experienced high trauma (n= 105), through the same analysis procedure.

**Supplementary Tables and Figures**

**Supplementary Table S1.** Neuroticism domains assessed in the UK Biobank.

| **UK Biobank ID** | **Neuroticism domain** | **Question provided** |
| --- | --- | --- |
| 1920 | Mood swings | Does your mood often go up and down? |
| 1930 | Miserableness | Do you ever feel ‘just miserable’ for no reason? |
| 1940 | Irritability | Are you an irritable person? |
| 1950 | Sensitivity/ hurt feelings | Are your feelings easily hurt? |
| 1960 | Fed-up feelings | Do you often feel ‘fed up? |
| 1970 | Nervous feelings | Would you call yourself a nervous person? |
| 1980 | Worrier/ anxious feelings | Are you a worrier? |
| 1990 | Tense/ ‘highly strung’ | Would you call yourself tense or ‘highly strung’? |
| 2000 | Worry too long after embarrassment | Do you worry too long after an embarrassing experience? |
| 2010 | Suffer from ‘nerves’ | Do you suffer from ‘nerves’? |
| 2020 | Loneliness/ isolation | Do you often feel lonely? |
| 2030 | Guilty feelings | Are you often troubled by feelings of guilt? |

| **Supplementary Table S2.** Full inclusion/exclusion criteria for anxious and control cases. | |
| --- | --- |
| **Narrow anxiety** | **Narrow control** |
| Inclusion criteria  Inclusion ICD10 anxiety codes (f.41270):  “F400”, “F401”, “F402”, “F408”, “F409”, “F410”, “F411”, “F412”, “F413”, “F418”, “F419”, “F420”, “F421”, “F422”, “F428”, “F429”, “F430”, “F431”, “F432”    Exclusion criteria  No pregnancy (f.3140)  No self-reported neurological disease (f.20002)  “1291”, “1289”, “1082”, “1083”, “1086”, “1524”, “1262”, “1397”, “1683”, “1245”, “1246”, “1491”, “1425”, “1433”, “1258”, “1263”, “1264”, “1266”, “1244”, “1583”, “1659”, “1259”, “1240”, “1434”  No self-reported mental health issues (aside from anxiety disorder and depression) (f.20002)  “1291”, “1289”, “1470”  No self-reported brain cancers (f.20001)  “1031”, “1032”  No self-reported bipolar, psychosis, or personality disorder (f.20544)  “Schizophrenia”, “Any other type of psychosis or psychotic illness”, “Mania, hypomania, bipolar or manic-depression”, “Autism, Asperger's or autistic spectrum disorder”, “A personality disorder”, “Bulimia nervosa”, “Psychological over-eating or binge-eating”, “Anorexia nervosa”, “ADHD”  Exclusion ICD10 codes (only if diagnosed prior to blood sampling) (f.41270):  *Mental health disorders:*  *-Manic episodes and bipolar disorder*  “F309”, “F308”, “F302”, “F301”, “F300”, “F319”, “F318”, “F317”, “F316”, “F315”, “F314”, “F313”, “F312”, “F311”, “F310”  *-Schizophrenia*  “F29”, “F28”, “F259”, “F258”, “F252”, “F251”, “F250”, “F24”**,** “F239”, “F238”, “F233”, “F232”, “F231”, “F230”, “F229”, “F228”, “F220”, “F21”, “F209”, “F208”, “F206”, “F205”, “F204”, “F203”, “F202”, “F201”, “F200”  *-Depression with psychotic symptoms*  “F333”, “F323”  *-Dissociative (conversion) disorders*  “F448”  *-Dementia, Alzheimer disease, cognitive impairments, and other neurological problems*  “F009”, “F002”, “F001”, “F000”, “F060”, “F051”, “F03”, “F028”, “F024”, “F023”, “F022”, “F021”, “F020”, “F019”, “F018”, “F013”, “F012”, “F199”, “F198”, “F197”, “F196”, “F195”, “F194”, “F193”, “F192”, “F191”, “F190”, “F620”, “F710”, “F711”, “F718”, “F719”, “F720”, “F721”, “F728”, “F729”, “F730”, “F731”, “F738”, “F739”, “F781”, “F788”, “F791”, “F798”  *-Autism Spectrum Disorders*  “F840”, “F841”, “F842”, “F843”, “F844”, “F845”, “F846”, “F847”, “F848”, “F849”  *-Eating Disorders*  “F500”, “F501”, “F502”, “F503”, “F504”, “F505”, “F508”, “F509”  *-ADHD*  “F900”, “F901”, “F908”, “F909”  *Diseases of the nervous system*  *-Inflammatory diseases of the central nervous system*  “G000”, “G001”, “G002”, “G003”, “G008”, “G009”, “G01”, “G020”, “G021”, “G028”, “G030”, “G031”, “G032”, “G038”, “G039”, “G040”, “G041”, “G042”, “G048”, “G049”, “G050”, “G051”, “G052”, “G058”, “G060”, “G061”, “G062”, “G07”, “G08”, “G09”  -*Systemic atrophies primarily affecting the central nervous system*  “G10”, “G110”, “G111”, “G112”, “G113”, “G114”, “G118”, “G119”, “G120”, “G121”, “G122”, “G128”, “G129”, “G130”, “G131”, “G132”, “G138”, “G14”  *-Extrapyramidal and movement disorders*  “G20”, “G21”, “G211”, “G212”, “G213”, “G214”, “G218”, “G219”, “G22”, “G230”, “G231”, “G232”, “G233”, “G238”, “G239”, “G240”, “G241”, “G242”, “G243”, “G244”, “G245”, “G248”, “G249”, “G250”, “G251”, “G252”, “G253”, “G254”, “G255”, “G256”, “G258”, “G259”, “G300”, “G301”, “G308”, “G309”, “G310”, “G311”, “G312”, “G318”, “G319”, “G320”, “G328”, “G35”, “G360”, “G361”, “G368”, “G369”, “G370”, “G371”, “G372”, “G373”, “G374”, “G375”, “G378”, “G379”, “G400”, “G401”, “G402”, “G403”, “G404”, “G405”, “G406”, “G407”, “G408”, “G409”, “G800”, “G801”, “G802”, “G803”, “G804”, “G808”, “G809”, “G810”, “G811”, “G819”, “G820”, “G821”, “G822”, “G823”, “G824”, “G825”, “G830”, “G831”, “G832”, “G833”, “G834”, “G835”, “G836”, “G838”, “G839”  *HIV*  “B200”, “B201”, “B202”, “B203”, “B204”, “B205”, “B206”, “B207”, “B208”, “B209”, “B210”, “B211”, “B212”, “B213”, “B217”, “B218”, “B219”, “B220”, “B221”, “B222”, “B227”, “B230”, “B231”, “B232”, “B238”, “B24”  *Tuberculosis*  “B900”, “B901”, “B902”, “B908”, “B909”  *Chronic viral hepatitis*  “B180”, “B181”, “B182”, “B188”, “B189”  *Immune disorders*  “D800”, “D801”, “D802”, “D803”, “D804”, “D805”, “D806”, “D807”, “D808”, “D809”, “D810”, “D811”, “D812”, “D813”, “D814”, “D815”, “D816”, “D817”, “D818”, “D819”, “D820”, “D821”, “D822”, “D823”, “D824”, “D828”, “D829”, “D830”, “D831”, “D832”, “D838”, “D839”, “D840”, “D841”, “D848”, “D849”, “D860”, “D861”, “D862”, “D863”, “D868”, “D869”, “D890”, “D891”, “D892”, “D893”, “D898”, “D899”  *Endocrine, nutritional, and metabolic disease*  *-Thyroid disorder*  “E000”, “E001”, “E002”, “E009”, “E010”, “E011”, “E012”, “E018”, “E030”, “E031”, “E032”, “E033”, “E034”, “E035”, “E038”, “E039”, “E040”, “E041”, “E042”, “E048”, “E049”, “E050”, “E051”, “E052”, “E053”, “E054”, “E055”, “E058”, “E059”, “E060”, “E061”, “E062”, “E063”, “E064”, “E065”, “E069”, “E070”, “E071”, “E078”, “E079”  - *Malnutrition*  “E40”, “E41”, “E42”, “E43”, “E44”, “E45”, “E46”  -*Metabolic disorders*  “E700”, “E701”, “E702”, “E703”, “E708”, “E709”, “E710”, “E711”,”E712”, “E713”, “E720”, “E721”, “E722”, “E723”, “E724”, “E725”, “E728”, “E729”,”E740”, “E741”, “E742”, “E743”, “E744”, “E748”, “E749”, “E750”, “E751”, “E752”, “E753”, “E754”, “E755”, “E756”, “E760”, “E761”, “E762”, “E763”,”E768”, “E769”, “E770”, “E771”, “E778”, “E779”, “E780”, “E782”, “E783”, “E784”, “E785”, “E786”, “E788”, “E789”, “E790”, “E791”, “E798”, “E799”,”E800”, “E801”, “E802”, “E803”, “E804”, “E805”, “E806”, “E807”  *Diseases of the liver*  *-Toxic liver disease*  “K710”, “K711”, “K712”, “K713”, “K714”, “K715”, “K716”, “K717”, “K718”, “K719”  *-Hepatic failure, not elsewhere classified*  “K721”  -*Chronic hepatitis, not elsewhere specified*  “K730”, “K731”, “K732”, “K738”, “K739”  -*Fibrosis and cirrhosis of liver*  “K743”, “K744”, “K745”, “K746”  -*Other inflammatory liver diseases*  “K754”  No use of antipsychotic or anticonvulsant medication (f.20003)  “1140856046”, “1140863416”, “1140867078”, “1140867092”, “1140867118”, “1140867134”, “1140867136”, “1140867150”, “1140867152”, “1140867168”, “1140867180”, “1140867184”, “1140867208”, “1140867210”, “1140867218”, “1140867244”, “1140867304”, “1140867306”, “1140867342”, “1140867398”, “1140867420”, “1140867444”, “1140867456”, “1140867546”, “1140867572”, “1140867944”, “1140867952”, “1140868120”, “1140868170”, “1140868172”, “1140872064”, “1140872072”, “1140872198”, “1140872200”, “1140872214”, “1140872216”, “1140872268”, “1140879658”, “1140879674”, “1140879746”, “1140879750”, “1140882098”, “1140882100”, “1140882320”, “1140909802”, “1140909804”, “1140910358”, “1140910976”, “1140927956”, “1140928260”, “1140928916”, “1141152848”, “1141152860”, “1141153490”, “1141167976”, “1141171566”, “1141172838”, “1141177762”, “1141195974”, “1141200458”, “1141201792”, “1141202024”, “2038459704” | Exclusion criteria  Exclusion ICD10 anxiety codes (f.41270):  “F400”, “F401”, “F402”, “F408”, “F409”, “F410”, “F411”, “F412”, “F413”, “F418”, “F419”, “F420”, “F421”, “F422”, “F428”, “F429”, “F430”, “F431”, “F432”  No pregnancy (f.3140)  No self-reported neurological disease (f.20002)  “1291”, “1289”, “1082”, “1083”, “1086”, “1524”, “1262”, “1397”, “1683”, “1245”, “1246”, “1491”, “1425”, “1433”, “1258”, “1263”, “1264”, “1266”, “1244”, “1583”, “1659”, “1259”, “1240”, “1434”  No self-reported mental health issues (aside from anxiety disorder and depression) (f.20002)  “1291”, “1289”, “1470”  No self-reported brain cancers (f.20001)  “1031”, “1032”  No self-reported bipolar, psychosis, or personality disorder (f.20544)  “Schizophrenia”, “Any other type of psychosis or psychotic illness”, “Mania, hypomania, bipolar or manic-depression”, “Autism, Asperger's or autistic spectrum disorder”, “A personality disorder”, “Bulimia nervosa”, “Psychological over-eating or binge-eating”, “Anorexia nervosa”, “ADHD”  Exclusion ICD10 codes (only if diagnosed prior to blood sampling) (f.41270):  *Mental health disorders:*  *Bipolar*  “F309”, “F308”, “F302”, “F301”, “F300”, “F319”, “F318”, “F317”, “F316”, “F315”, “F314”, “F313”, “F312”, “F311”, “F310”  *Schizophrenia*  “F29”, “F28”, “F259”, “F258”, “F252”, “F251”, “F250”, “F24”**,** “F239”, “F238”, “F233”, “F232”, “F231”, “F230”, “F229”, “F228”, “F220”, “F21”, “F209”, “F208”, “F206”, “F205”, “F204”, “F203”, “F202”, “F201”, “F200”  *Depression with psychotic symptoms*  “F333”, “F323”,”F448”,”F009”, “F002”, “F001”, “F000”, “F060”, “F051”, “F03”, “F028”, “F024”, “F023”, “F022”, “F021”, “F020”, “F019”, “F018”, “F013”, “F012”, “F199”, “F198”, “F197”, “F196”, “F195”, “F194”, “F193”, “F192”, “F191”, “F190”, “F620”, “F710”, “F711”, “F718”, “F719”, “F720”, “F721”, “F728”, “F729”, “F730”, “F731”, “F738”, “F739”, “F781”, “F788”, “F791”, “F798”  *Autism Spectrum Disorders*  “F840”, “F841”, “F842”, “F843”, “F844”, “F845”, “F846”, “F847”, “F848”, “F849”  *Eating Disorders*  “F500”, “F501”, “F502”, “F503”, “F504”, “F505”, “F508”, “F509”  *ADHD*  “F900”, “F901”, “F908”, “F909”  *Diseases of the nervous system*  *-Inflammatory diseases of the central nervous system*  “G000”, “G001”, “G002”, “G003”, “G008”, “G009”, “G01”, “G020”, “G021”, “G028”, “G030”, “G031”, “G032”, “G038”, “G039”, “G040”, “G041”, “G042”, “G048”, “G049”, “G050”, “G051”, “G052”, “G058”, “G060”, “G061”, “G062”, “G07”, “G08”, “G09”  -*Systemic atrophies primarily affecting the central nervous system*  “G10”, “G110”, “G111”, “G112”, “G113”, “G114”, “G118”, “G119”, “G120”, “G121”, “G122”, “G128”, “G129”, “G130”, “G131”, “G132”, “G138”, “G14”  -Extrapyramidal and movement disorders  “G20”, “G21”, “G211”, “G212”, “G213”, “G214”, “G218”, “G219”, “G22”, “G230”, “G231”, “G232”, “G233”, “G238”, “G239”, “G240”, “G241”, “G242”, “G243”, “G244”, “G245”, “G248”, “G249”, “G250”, “G251”, “G252”, “G253”, “G254”, “G255”, “G256”, “G258”, “G259”, “G300”, “G301”, “G308”, “G309”, “G310”, “G311”, “G312”, “G318”, “G319”, “G320”, “G328”, “G35”, “G360”, “G361”, “G368”, “G369”, “G370”, “G371”, “G372”, “G373”, “G374”, “G375”, “G378”, “G379”, “G400”, “G401”, “G402”, “G403”, “G404”, “G405”, “G406”, “G407”, “G408”, “G409”, “G800”, “G801”, “G802”, “G803”, “G804”, “G808”, “G809”, “G810”, “G811”, “G819”, “G820”, “G821”, “G822”, “G823”, “G824”, “G825”, “G830”, “G831”, “G832”, “G833”, “G834”, “G835”, “G836”, “G838”, “G839”  *HIV*  “B200”, “B201”, “B202”, “B203”, “B204”, “B205”, “B206”, “B207”, “B208”, “B209”, “B210”, “B211”, “B212”, “B213”, “B217”, “B218”, “B219”, “B220”, “B221”, “B222”, “B227”, “B230”, “B231”, “B232”, “B238”, “B24”  *Tuberculosis*  “B900”, “B901”, “B902”, “B908”, “B909”  *Chronic viral hepatitis*  “B180”, “B181”, “B182”, “B188”, “B189”  *Certain disorders involving the immune system*  “D800”, “D801”, “D802”, “D803”, “D804”, “D805”, “D806”, “D807”, “D808”, “D809”, “D810”, “D811”, “D812”, “D813”, “D814”, “D815”, “D816”, “D817”, “D818”, “D819”, “D820”, “D821”, “D822”, “D823”, “D824”, “D828”, “D829”, “D830”, “D831”, “D832”, “D838”, “D839”, “D840”, “D841”, “D848”, “D849”, “D860”, “D861”, “D862”, “D863”, “D868”, “D869”, “D890”, “D891”, “D892”, “D893”, “D898”, “D899”  *Endocrine, nutritional, and metabolic disease*  *-Thyroid disorder*  “E000”, “E001”, “E002”, “E009”, “E010”, “E011”, “E012”, “E018”, “E030”, “E031”, “E032”, “E033”, “E034”, “E035”, “E038”, “E039”, “E040”, “E041”, “E042”, “E048”, “E049”, “E050”, “E051”, “E052”, “E053”, “E054”, “E055”, “E058”, “E059”, “E060”, “E061”, “E062”, “E063”, “E064”, “E065”, “E069”, “E070”, “E071”, “E078”, “E079”  - *Malnutrition*  “E40”, “E41”, “E42”, “E43”, “E44”, “E45”, “E46”  -*Metabolic disorders*  “E700”, “E701”, “E702”, “E703”, “E708”, “E709”, “E710”, “E711”,”E712”, “E713”, “E720”, “E721”, “E722”, “E723”, “E724”, “E725”, “E728”, “E729”,”E740”, “E741”, “E742”, “E743”, “E744”, “E748”, “E749”, “E750”, “E751”, “E752”, “E753”, “E754”, “E755”, “E756”, “E760”, “E761”, “E762”, “E763”,”E768”, “E769”, “E770”, “E771”, “E778”, “E779”, “E780”, “E782”, “E783”, “E784”, “E785”, “E786”, “E788”, “E789”, “E790”, “E791”, “E798”, “E799”,”E800”, “E801”, “E802”, “E803”, “E804”, “E805”, “E806”, “E807”  *Diseases of the liver*  *-Toxic liver disease*  “K710”, “K711”, “K712”, “K713”, “K714”, “K715”, “K716”, “K717”, “K718”, “K719”  *-Hepatic failure, not elsewhere classified*  “K721”  -*Chronic hepatitis, not elsewhere specified*  “K730”, “K731”, “K732”, “K738”, “K739”  -*Fibrosis and cirrhosis of liver*  “K743”, “K744”, “K745”, “K746”  -*Other inflammatory liver diseases*  “K754”  No use of antipsychotic or anticonvulsant medication (f.20003)  “1140856046”, “1140863416”, “1140867078”, “1140867092”, “1140867118”, “1140867134”, “1140867136”, “1140867150”, “1140867152”, “1140867168”, “1140867180”, “1140867184”, “1140867208”, “1140867210”, “1140867218”, “1140867244”, “1140867304”, “1140867306”, “1140867342”, “1140867398”, “1140867420”, “1140867444”, “1140867456”, “1140867546”, “1140867572”, “1140867944”, “1140867952”, “1140868120”, “1140868170”, “1140868172”, “1140872064”, “1140872072”, “1140872198”, “1140872200”, “1140872214”, “1140872216”, “1140872268”, “1140879658”, “1140879674”, “1140879746”, “1140879750”, “1140882098”, “1140882100”, “1140882320”, “1140909802”, “1140909804”, “1140910358”, “1140910976”, “1140927956”, “1140928260”, “1140928916”, “1141152848”, “1141152860”, “1141153490”, “1141167976”, “1141171566”, “1141172838”, “1141177762”, “1141195974”, “1141200458”, “1141201792”, “1141202024”, “2038459704”  **AND**  Baseline GAD7 < 5*  **AND**  CIDI-SF negative result**  **AND**  PCL-6 ≤ 13***  **AND**  No self-reported anxiety at the time of initial assessment (f.20002)  “1287”, “1615”, “1469”, “1288”  **AND**  Self-reported anxiety, bipolar, psychosis, or personality disorder (f.20544)  “Social anxiety or social phobia”, “Anxiety, nerves or generalized anxiety disorder”, “Panic attacks”, “Obsessive compulsive disorder (OCD), “Agoraphobia”, “Any other phobia (e.g. disabling fear of heights or spiders)”  **AND**  Exclude if ever seen doctor (GP) for nerves, anxiety, tension, or depression (f.2090)  “Yes”, “Do not know”, “Prefer not to answer”  **AND**  Exclude if ever seen a psychiatrist for nerves, anxiety, tension, or depression (f.2100)  “Yes”, “Do not know”, “Prefer not to answer”  **AND**  Ever felt worried, tense, or anxious for most of a month or longer (f. 20421)  “Yes”, “Do not know”, “Prefer not to answer”  **AND**  Ever been offered/sought treatment for anxiety (f.21062)  “Yes”  **AND**  Recent feelings or nervousness or anxiety (f.20506)  “Nearly every day”, “More than half the days”  **AND**  Ever worried more than most people would in similar situation (f.20425)  “Yes”  **AND**  Frequency of tenseness/restlessness in last 2 weeks (f.2070)  “More than half the days”, “Nearly every day”  **AND**  Ever sought or received professional help for mental distress (f.20499)  “Yes”  **AND**  Ever suffered mental distress preventing usual activities (f.20500)  “Yes”  **AND**  No antidepressant or anxiolytic medication:  “1201”, “1140855824”, “1140855832”, “1140855870”, “1140855890”, “1140856040”, “1140862810”, “1140863016”, “1140863028”, “1140863036”, “1140863106”, “1140863110”, “1140863112”, “1140863120”, “1140863144”, “1140863152”, “1140863176”, “1140863182”, “1140863194”, “1140863202”, “1140863286”, “1140863292”, “1140863302”, “1140863308”, “1140863310”, “1140863328”, “1140863350”, “1140863372”, “1140863378”, “1140863410”, “1140863440”, “1140863442”, “1140863454”, “1140864916”, “1140865016”, “1140867624”, “1140867640”, “1140867668”, “1140867690”, “1140867712”, “1140867726”, “1140867756”, “1140867758”, “1140867784”, “1140867812”, “1140867818”, “1140867820”, “1140867850”, “1140867852”, “1140867856”, “1140867860”, “1140867876”, “1140867878”, “1140867884”, “1140867888”, “1140867914”, “1140867916”, “1140867920”, “1140867922”, “1140867934”, “1140867938”, “1140867942”, “1140867948”, “1140867960”, “1140875434”, “1140879540”, “1140879544”, “1140879556”, “1140879616”, “1140879620”, “1140879628”, “1140879630”, “1140879634”, “1140879730”, “1140882082”, “1140882236”, “1140882244”, “1140882312”, “1140883656”, “1140909798”, “1140909800”, “1140909806”, “1140910504”, “1140910704”, “1140910820”, “1140916282”, “1140916288”, “1140917460”, “1140921600”, “1140928004”, “1141151946”, “1141151978”, “1141151982”, “1141152732”, “1141152736”, “1141157496”, “1141174756”, “1141180212”, “1141190158”, “1141200564”, “1141200570”, “1141201834”  **AND**  No mood stabilisers:  “1140863268”, “1140867490”, “1140867494”, “1140867498”, “1140867500”, “1140867504”, “1140867518”, “1140867520”, “1140872290”, “1141200004” |

Note: UK Biobank codes beginning with ‘f.’

***Generalised Anxiety Disorder 7-item (GAD7) scoring criteria**

- Each answer is scored as 0-3 and summed up; BioBank codes the scores as 1-4, therefore subtract 7 to adjust
- Mild anxiety: GAD7 5-10; Moderate anxiety: GAD7 10-15; Severe anxiety: GAD7 >15

1. Recent feelings of nervousness or anxiety (f.20506)
2. Recent feelings of foreboding (f.20512)
3. Recent inability to stop or control worrying (f.20509)
4. Recent worrying too much about different things (f.20520)
5. Recent trouble relaxing (f.20515)
6. Recent restlessness (f.20516)
7. Recent easy annoyance or irritability (f.20505)

****Composite International Diagnostic Interview short-form (CIDI-SF) anxiety scoring criteria**

-If the following answers are provided this indicates anxiety disorder

1. Ever felt worried, tense or anxious for most of a month or longer (f.20421)

- “Yes”

1. Longest period spent worried or anxious (f.20420)

- 6 months or longer

1. Ever worried more than most people would in a similar situation (f.20425)

- “Yes”

**OR**

Stronger worrying (than other people) during period of worst anxiety (f.20542)

- “Yes”

1. Worried most days during period of worst anxiety (f.20538)

- “Yes”

1. Multiple worries during worst period of anxiety (f.20540)

- “Yes”

**OR**

Number of things worried about during worst period of anxiety (f.20543)

- “More than one thing”

1. Difficulty stopping worrying during worst period of anxiety (f.20541)

- “Yes”

**OR**

Frequency or inability to stop worrying during worst period of anxiety (f.20539)

- “Often”

**OR**

Frequency or difficulty controlling worry during worst period of anxiety (f.20537)

- “Often”

1. Impact on normal roles during worst period of anxiety (f.20418)

- “Somewhat” OR “A lot”

1. Experienced a total of three or more of the below symptoms during worst period of anxiety:

- Restlessness (f.20426)
- Keyed up or on edge (f.20423)
- Easily tired (f.20429)
- Difficulty concentrating (f.20419)
- More irritable than usual (f.20422)
- Tense, sore, or aching muscles (f.20417)
- Frequent trouble falling or staying asleep (f.20427)

*****Posttraumatic Stress Disorder Checklist 6-item (PCL-6) scoring criteria**

- Each answer is scored as 1-5; BioBank codes as 0-4, therefore subtract 5 to adjust.
- PCL-6 >=14 indicates presence of post-traumatic stress disorder

1. Repeated disturbing thoughts of stressful experience in past month (f.20497)
2. Avoided activities or situations because of previous stressful experience in past month (f.20495)
3. Felt very upset when reminded of stressful experience in past month (f.20498)
4. Felt distant from other people in past month (f.20496)
5. Felt irritable or had angry outbursts in past month (f.20494)
6. Trouble concentrating (f.20508)

**Supplementary Table S3.** Demographics of the lifetime anxiety free control cohort compared to individuals diagnosed with anxiety (denoted by an ICD-10 anxiety coding of F40-3) prior to blood sampling.

|  | **Control average (n= 45,949)** | **Retrospective anxiety average (n= 214)** | **Unadjusted p-value** |
| --- | --- | --- | --- |
| Age, years (f.21003) | 56.6 | 55.3 | 0.01 |
| Sex, % female (f.31) | 46.1 | 63.6 | 4.74x10^-7^ |
| Townsend Deprivation Index (f.22189) | -1.62 | -0.49 | 1.88x10^-6^ |
| Ethnicity, % Caucasian (f.21000) | 95.4 | 98.1 | 0.13 |
| Number of self-reported non-cancer illnesses (f.135)  % Diabetes (f.2443)  % Angina (f.6150)  % Heart attack (f.6150)  % Hypertension (f.6150) | 1.43  4.32  1.64  1.32  24.1 | 3.05  5.24  5.14  3.27  37.4 | <2.2x10^-16^  4.15x10^-13^  0.0003  0.04  3.38x10^-5^ |
| Cancer (%) (f.2453.0.0) | 6.90 | 7.48 | 0.93 |
| Number of medications taken (f.137) | 1.85 | 3.98 | <2.2x10^-16^ |
| BMI (f.21001) | 27.3 | 28.6 | 0.0008 |
| Smoking status (%) (f.20116)  Current  Previous  Never | 9.22  33.1  57.7 | 19.2  36.2  44.6 | 1.24x10^-6^ |
| Alcohol intake frequency (%) (f.1558)  Daily or almost daily  3-4 per week  1-2 per week  1-3 per month  Special occasions only  Never | 21.6  25.2  27.0  10.5  9.55  6.18 | 21.1  16.0  26.8  10.8  12.2  13.1 | 2.62x10^-6^ |
| Overall health rating (%) (f.2178)  Excellent  Good  Fair  Poor | 21.6  61.5  15.2  1.71 | 6.60  41.0  38.2  14.2 | <2.2x10^-16^ |
| Long-standing illness or disability (%) (f.2188) | 23.5 | 56.3 | <2.2x10^-16^ |
| Employment status (%) (f.6142)  Employed  Retired  Looking after home/family  Sickness/disability prevents work  Unemployed  Unpaid/voluntary work  Other | 61.0  35.4  4.26  1.20  1.52  3.06  1.09 | 45.3  30.4  7.01  17.3  3.27  5.14  1.87 | 1.26x10^-5^  0.23  0.10  <2.2x10^-16^  0.08  0.16  0.41 |
| Stress/trauma in past 2 years (%) (f.6145)  Serious illness/injury/assault  Serious illness/injury/assault close relative  Death of close relative  Death of spouse/partner  Marital separation/divorce  Financial difficulties  None of the above | 6.35  9.91  20.5  1.27  2.20  8.33  61.2 | 20.4  12.8  19.4  2.84  9.00  21.3  46.9 | <2.2x10^-16^  0.004  0.01  0.001  2.86x10^-12^  1.33x10^-12^  1.51x10^-6^ |
| Physical pain enough to interfere with daily life experienced in last month (%) (f.6159) | 53.3 | 78.0 | 3.57x10^-12^ |

Note: UK Biobank codes beginning with ‘f.’

**Supplementary Table S4.** Demographics of the lifetime anxiety free control cohort compared to individuals diagnosed with anxiety (denoted by an ICD-10 anxiety coding of F40-3) any time after blood sampling.

|  | **Control average (n= 45,949)** | **All years prospective anxiety disorder average (n= 3,773)** | **Unadjusted p-value** |
| --- | --- | --- | --- |
| Age, years (f.21003) | 56.6 | 57.0 | 0.007 |
| Sex, % female (f.31) | 46.1 | 67.3 | <2.2x10^-16^ |
| Townsend Deprivation Index (f.22189) | -1.62 | -1.02 | <2.2x10^-16^ |
| Ethnicity, % Caucasian (f.21000) | 95.4 | 96.4 | 0.01 |
| Number of self-reported non-cancer illnesses (f.135)  % Diabetes (f.2443)  % Angina (f.6150)  % Heart attack (f.6150)  % Hypertension (f.6150) | 1.43  4.32  1.64  1.32  24.1 | 2.47  5.95  2.79  1.49  31.0 | <2.2x10^-16^  3.42x10^-6^  1.31x10^-6^  0.67  <2.2x10^-16^ |
| Cancer (%) (f.2453) | 6.90 | 8.73 | 1.08x10^-6^ |
| Number of medications taken (f.137) | 1.85 | 3.21 | <2.2x10^-16^ |
| BMI (f.21001) | 27.3 | 27.9 | 8.68x10^-12^ |
| Smoking status (%) (f.20116.0.0)  Current  Previous  Never | 9.22  33.1  57.7 | 14.8  36.6  48.5 | <2.2x10^-16^ |
| Alcohol intake frequency (%) (f.1558)  Daily or almost daily  3-4 per week  1-2 per week  1-3 per month  Special occasions only  Never | 21.6  25.2  27.0  10.5  9.55  6.18 | 19.0  18.7  24.2  12.4  15.4  10.2 | <2.2x10^-16^ |
| Overall health rating (%) (f.2178)  Excellent  Good  Fair  Poor | 21.6  61.5  15.2  1.71 | 9.16  52.8  29.0  8.98 | <2.2x10^-16^ |
| Long-standing illness or disability (%) (f.2188) | 23.5 | 44.5 | <2.2x10^-16^ |
| Employment status (%) (f.6142)  Employed  Retired  Looking after home/family  Sickness/disability prevents work  Unemployed  Unpaid/voluntary work  Other | 61.0  35.4  4.26  1.20  1.52  3.06  1.09 | 49.7  39.1  5.72  8.46  1.78  4.20  1.38 | <2.2x10^-16^  1.57x10^-5^  8.02x10^-5^  <2.2x10^-16^  0.26  0.0003  0.14 |
| Stress/trauma in past 2 years (%) (f.6145)  Serious illness/injury/assault  Serious illness/injury/assault close relative  Death of close relative  Death of spouse/partner  Marital separation/divorce  Financial difficulties  None of the above | 6.35  9.91    20.5  1.27  2.20  8.33  61.2 | 11.3  12.6    24.0  2.13  4.05  19.4  48.1 | <2.2x10^-16^  2.96x10^-7^  1.41x10^-6^  2.01x10^-5^  1.97x10^-12^  <2.2x10^-16^  <2.2x10^-16^ |
| Physical pain enough to interfere with daily life experienced in last month (%) (f.6159) | 53.3 | 72.4 | <2.2x10^-16^ |

Note: UK Biobank codes beginning with ‘f.’

**Supplementary Table S5.** Demographics of the lifetime anxiety free control cohort compared to individuals diagnosed with anxiety (denoted by an ICD-10 anxiety coding of F40-3) up to five years after blood sampling.

|  | **Control average (n=45,949)** | **1-5-years prospective anxiety disorder average (n= 627)** | **Unadjusted p-value** |
| --- | --- | --- | --- |
| Age, years (f.21003) | 56.6 | 56.3 | 0.28 |
| Sex, % female (f.31) | 46.1 | 68.6 | <2.2x10^-16^ |
| Townsend Deprivation Index (f.22189) | -1.62 | -0.64 | 5.68x10^-13^ |
| Ethnicity, % Caucasian (f.21000) | 95.4 | 96.2 | 0.57 |
| Number of self-reported non-cancer illnesses (f.135)  % Diabetes (f.2443)  % Angina (f.6150)  % Heart attack (f.6150)  % Hypertension (f.6150) | 1.43  4.32  1.64  1.32  24.1 | 2.70  5.58  3.51  0.96  34.5 | <2.2x10^-16^  0.37  0.001  0.65  1.29x10^-8^ |
| Cancer (%) (f.2453) | 6.90 | 75.3 | 0.14 |
| Number of medications taken (f.137) | 1.85 | 3.54 | <2.2x10^-16^ |
| BMI (f.21001) | 27.3 | 28.6 | 2.44x10^-8^ |
| Smoking status (%) (f.20116)  Current  Previous  Never | 9.22  33.1  57.7 | 17.9  35.5  46.6 | 6.25x10^-14^ |
| Alcohol intake frequency (%) (f.1558)  Daily or almost daily  3-4 per week  1-2 per week  1-3 per month  Special occasions only  Never | 21.6  25.2  27.0  10.5  9.55  6.18 | 17.7  18.8  25.4  10.7  16.7  10.7 | 7.60x10^-13^ |
| Overall health rating (%) (f.2178)  Excellent  Good  Fair  Poor | 21.6  61.5  15.2  1.71 | 7.35  47.0  32.9  12.8 | <2.2x10^-16^ |
| Long-standing illness or disability (%) (f.2188) | 23.5 | 48.4 | <2.2x10^-16^ |
| Employment status (%) (f.6142)  Employed  Retired  Looking after home/family  Sickness/disability prevents work  Unemployed  Unpaid/voluntary work  Other | 61.0  35.4  4.26  1.20  1.52  3.06  1.09 | 49.0  36.6  3.68  12.5  1.6  4.00  2.72 | 7.51x10^-9^  0.80  0.76  <2.2x10^-16^  0.97  0.39  0.0005 |
| Stress/trauma in past 2 years (%) (f.6145)  Serious illness/injury/assault  Serious illness/injury/assault close relative  Death of close relative  Death of spouse/partner  Marital separation/divorce  Financial difficulties  None of the above | 6.35  9.91    20.5  1.27  2.20  8.33  61.2 | 10.4  13.8    26.4  2.40  3.85  21.6  45.2 | 0.0001  0.004  0.0009  0.03  0.01  <2.2x10^-16^  <2.2x10^-16^ |
| Physical pain enough to interfere with daily life experienced in last month (%) (f.6159) | 53.3 | 76.2 | <2.2x10^-16^ |

Note: UK Biobank codes beginning with ‘f.’

**Supplementary Table S6.** Relative breakdown of anxiety disorder diagnoses within anxious populations classified by ICD-10 coding.

| ICD10 Code | Retrospective (%) | | Prospective all (%) | | Prospective 1-5 years (%) | |
| --- | --- | --- | --- | --- | --- | --- |
|  | Matched (n= 205) | Unmatched (n= 214) | Matched (n= 3,701) | Unmatched (n= 3,773) | Matched (n= 617) | Unmatched (n= 627) |
| F400 | 2.0 | 1.9 | 0.3 | 0.3 | 0.0 | 0.0 |
| F401 | 0.5 | 0.5 | 0.1 | 0.1 | 0.2 | 0.2 |
| F402 | 10.2 | 9.8 | 9.1 | 9.0 | 12.0 | 11.8 |
| F408 | 0.0 | 0.0 | 0.5 | 0.5 | 0.5 | 0.5 |
| F409 | 0.0 | 0.0 | 0.2 | 0.2 | 0.5 | 0.5 |
| F410 | 14.1 | 14.5 | 6.9 | 6.8 | 10.9 | 10.7 |
| F411 | 3.9 | 3.7 | 1.7 | 1.7 | 1.9 | 1.9 |
| F412 | 22.4 | 23.3 | 16.3 | 16.0 | 23.8 | 23.4 |
| F413 | 0.0 | 0.0 | 0.1 | 0.1 | 0.0 | 0.0 |
| F418 | 1.5 | 1.4 | 0.4 | 0.4 | 0.2 | 0.2 |
| F419 | 55.6 | 56.1 | 78.7 | 77.2 | 73.1 | 71.9 |
| F420 | 1.0 | 0.9 | 0.1 | 0.1 | 0.5 | 0.5 |
| F421 | 1.0 | 0.9 | 0.0 | 0.0 | 0.0 | 0.0 |
| F422 | 1.0 | 0.9 | 0.0 | 0.0 | 0.0 | 0.0 |
| F428 | 0.0 | 0.0 | 0.0 | 0.0 | 0.0 | 0.0 |
| F429 | 1.0 | 1.4 | 0.9 | 0.9 | 1.0 | 1.0 |
| F430 | 6.3 | 6.1 | 0.4 | 0.4 | 0.6 | 0.6 |
| F431 | 0.5 | 0.5 | 1.7 | 1.7 | 1.6 | 1.6 |
| F432 | 10.7 | 10.7 | 0.6 | 0.6 | 1.3 | 1.3 |

F400, agoraphobia; F401, social phobias; F402, specific (isolated) phobias; F408, other phobic anxiety disorders; F409, phobic anxiety disorders, unspecified; F410, panic disorder, episodic paroxysmal anxiety; F411, generalized anxiety disorder; F412, mixed anxiety disorders; F413, other mixed anxiety disorders; F418, other specified anxiety disorders; F419, anxiety disorder, unspecified; F420, predominantly obsessional thoughts or ruminations; F421, predominantly compulsive acts, obsessional rituals; F422, mixed obsessional thoughts and acts; F428, other obsessive-compulsive disorders; F429, obsessive-compulsive disorder, unspecified; F430, acute stress reaction; F431, post-traumatic stress disorder; F432, adjustment disorders.


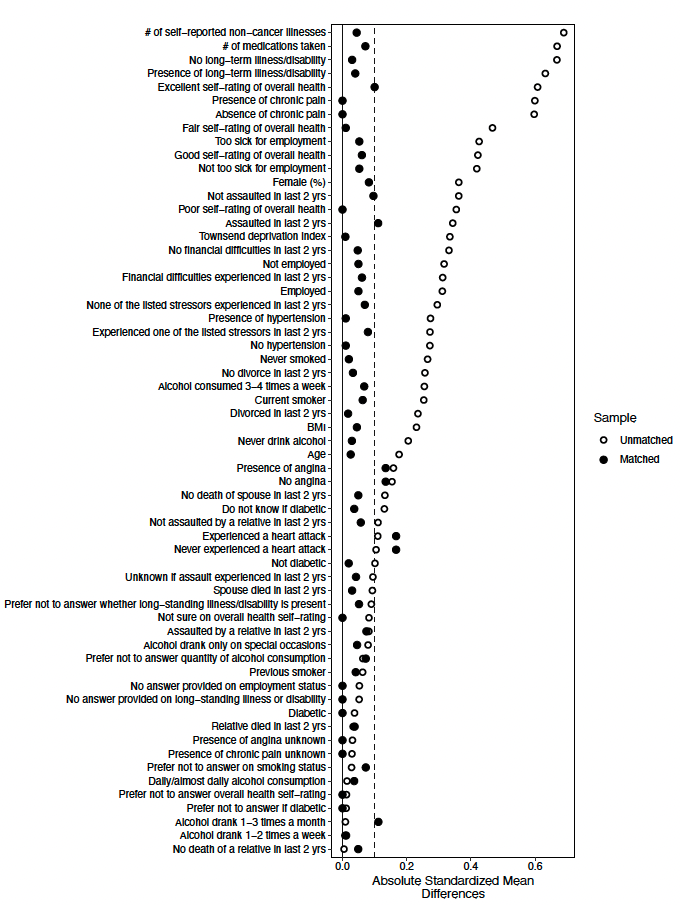


**Supplementary Figure S1.** Quality of matching in retrospective anxious cohort compared to control cohorts. Absolute standardised mean differences between the anxious and control cohort prior to matching represented (represented with white dots), and after matching (represented with black dots).


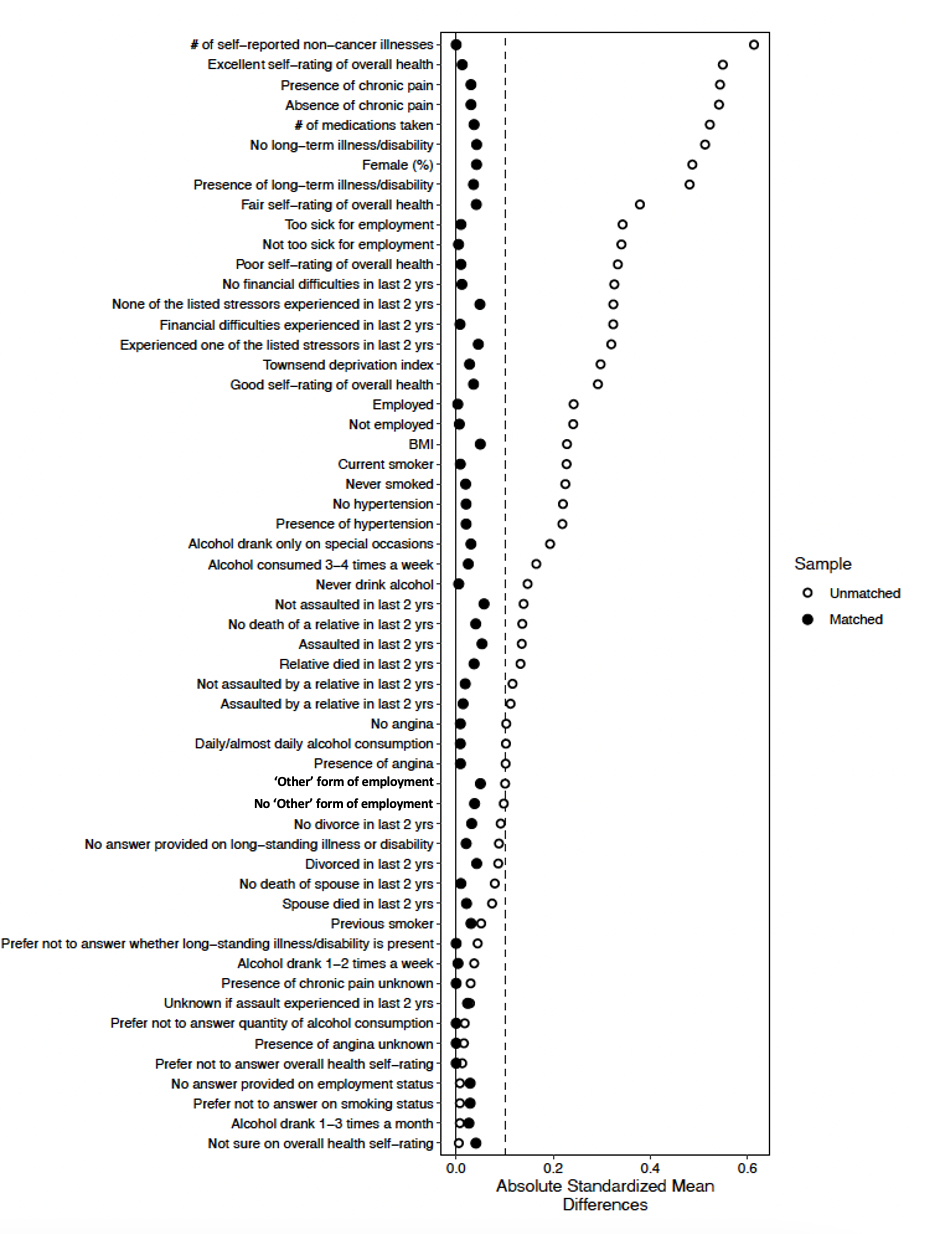
**Supplementary Figure S2.** Quality of matching in 1-5-year prospective anxious cohort compared to control cohorts*.* Absolute standardised mean differences between the anxious and control cohort prior to matching (represented with white dots), and after matching (represented with black dots).


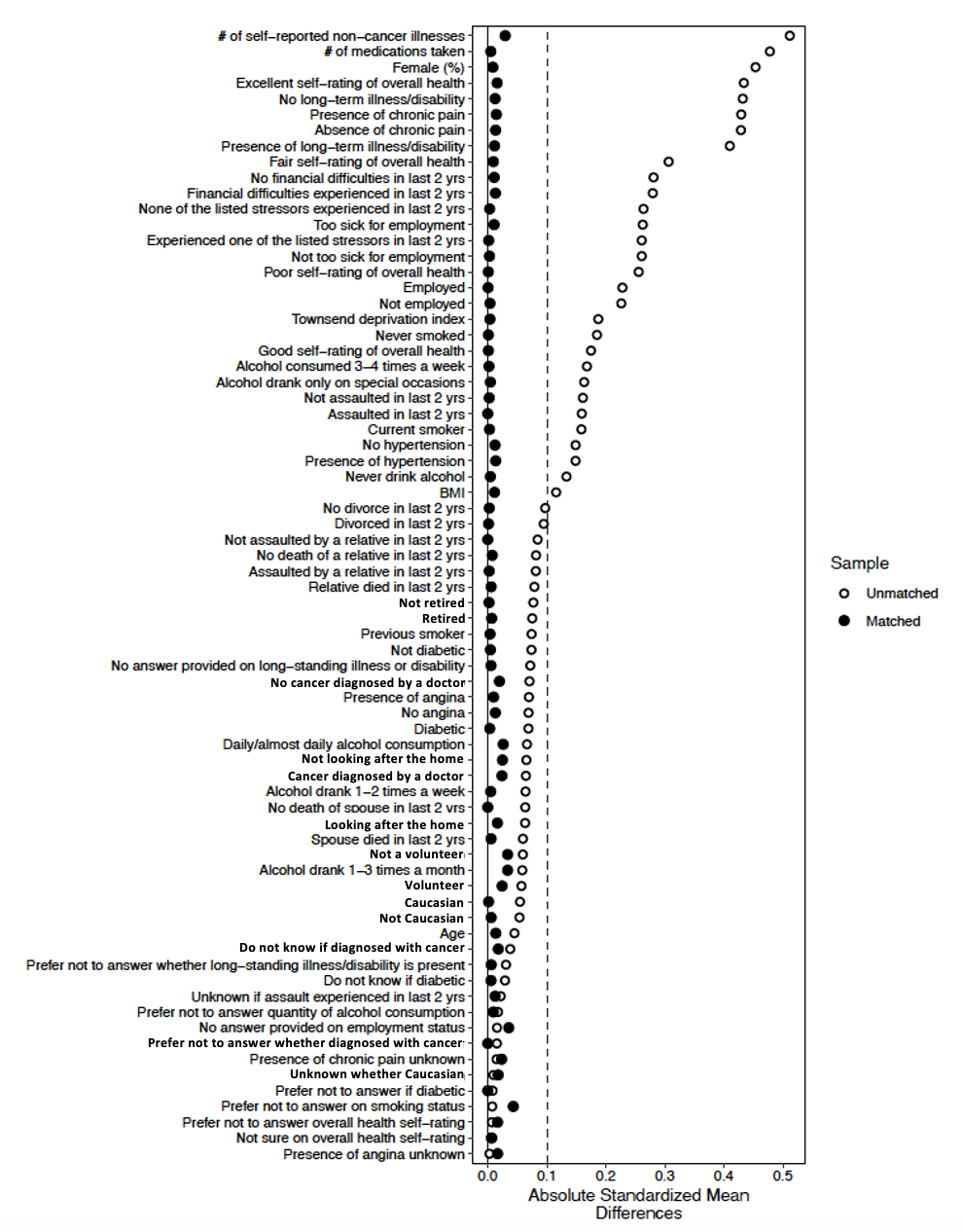


**Supplementary Figure S3.** Quality of matching in all prospective anxious cohort compared to control cohorts. Absolute standardised mean differences between the anxious and control cohort prior to matching (represented with white dots), and after matching (represented with black dots).

**Supplementary Table S7.** Demographics of retrospective anxious cohort (denoted by an ICD-10 anxiety coding of F40-3) compared to the matched control cohort.

|  | **Resilient average (n= 205)** | **Retrospective anxiety average (n= 205)** | **Unadjusted p-value** |
| --- | --- | --- | --- |
| Age, years (f.21003) | 54.7 | 55.6 | 0.27 |
| Sex, % female (f.31) | 65.4 | 63.9 | 0.84 |
| Townsend Deprivation Index (f.22189) | -0.57 | -0.59 | 0.96 |
| Ethnicity, % Caucasian (f.21000) | 97.5 | 98.0 | 0.94 |
| Number of self-reported non-cancer illnesses (f.135)  % Diabetes (f.2443)  % Angina (f.6150)  % Heart attack (f.6150)  % Hypertension (f.6150) | 2.75  5.91  3.90  3.90  35.6 | 2.91  5.42  5.37  3.41  37.1 | 0.47  0.98  0.64  1.00  0.84 |
| Cancer (%) (f.2453) | 11.2 | 6.34 | 0.12 |
| Number of medications taken (f.137) | 3.97 | 3.87 | 0.78 |
| BMI (f.21001) | 28.6 | 28.4 | 0.71 |
| Smoking status (%) (f.20116)  Current  Previous  Never | 14.7  41.7  43.6 | 17.2  36.8  46.1 | 0.77 |
| Alcohol intake frequency (%) (f.1558)  Daily or almost daily  3-4 per week  1-2 per week  1-3 per month  Special occasions only  Never | 26.0  14.2  26.0  9.80  16.7  7.35 | 21.1  16.7  26.0  10.3  12.7  13.2 | 0.43 |
| Overall health rating (%) (f.2178)  Excellent  Good  Fair  Poor | 9.31  41.7  38.2  10.8 | 6.90  42.4  37.9  12.8 | 0.84 |
| Long-standing illness or disability (%) (f.2188) | 53.7 | 54.5 | 0.63 |
| Employment status (%) (f.6142)  Employed  Retired  Looking after home/family  Sickness/disability prevents work  Unemployed  Unpaid/voluntary work  Other | 53.1  29.8  7.80  12.7  2.44  3.41  1.46 | 46.8  31.2  7.32  14.6  2.93  4.88  1.95 | 0.24  0.83  1.00  0.67  1.00  0.62  1.00 |
| Stress/trauma in past 2 years (%) (f.6145)  Serious illness/injury/assault  Serious illness/injury/assault close relative  Death of close relative  Death of spouse/partner  Marital separation/divorce  Financial difficulties  None of the above | 22.3  8.42  17.3  4.95  8.91  20.3  46.5 | 18.3  12.4  19.8  2.97  6.93  18.8  49.0 | 0.61  0.43  0.81  0.59  0.76  0.93  0.88 |
| Physical pain enough to interfere with daily life experienced in last month (%) (f.6159) | 78.5 | 77.1 | 0.81 |

Note: UK Biobank codes beginning with ‘f.’

**Supplementary Table S8**. Demographics of 1–5-year prospective anxious (denoted by an ICD-10 diagnoses of F40-3) against matched control cohorts.

|  | **Resilient average (n= 617)** | **1–5-year prospective anxiety average (n= 617)** | **Unadjusted p-value** |
| --- | --- | --- | --- |
| Age, years (f.21003) | 57.2 | 56.3 | 0.08 |
| Sex, % female (f.31) | 70.0 | 68.4 | 0.67 |
| Townsend Deprivation Index (f.22189) | -0.53 | -0.64 | 0.58 |
| Ethnicity, % Caucasian (f.21000) | 93.0 | 96.1 | 0.05 |
| Number of self-reported non-cancer illnesses (f.135)  % Diabetes (f.2443)  % Angina (f.6150)  % Heart attack (f.6150)  % Hypertension (f.6150) | 2.67  9.25  3.74  2.11  35.0 | 2.60  5.19  3.57  0.81  34.1 | 0.57  0.01  0.84  0.14  0.80 |
| Cancer (%) (f.2453) | 10.6 | 7.49 | 0.10 |
| Number of medications taken (f.137) | 3.38 | 3.41 | 0.84 |
| BMI (f.21001) | 28.7 | 28.6 | 0.83 |
| Smoking status (%) (f.20116)  Current  Previous  Never | 15.6  36.5  47.9 | 17.4  35.7  46.9 | 0.87 |
| Alcohol intake frequency (%) (f.1558)  Daily or almost daily  3-4 per week  1-2 per week  1-3 per month  Special occasions only  Never | 15.6  19.1  26.3  10.7  17.7  10.7 | 17.8  19.0  25.3  10.7  16.5  10.7 | 0.93 |
| Overall health rating (%) (f.2178)  Excellent  Good  Fair  Poor | 6.00  47.6  36.5  9.89 | 7.47  47.7  33.4  11.4 | 0.49 |
| Long-standing illness or disability (%) (f.2188) | 48.3 | 47.6 | 0.55 |
| Employment status (%) (f.6142)  Employed  Retired  Looking after home/family  Sickness/disability prevents work  Unemployed  Unpaid/voluntary work  Other | 49.4  36.1  4.54  10.4  1.78  3.24  2.59 | 49.6  36.9  3.58  11.2  1.63  4.07  2.76 | 0.37  0.35  0.25  0.33  0.36  0.27  0.36 |
| Stress/trauma in past 2 years (%) (f.6145)  Serious illness/injury/assault  Serious illness/injury/assault close relative  Death of close relative  Death of spouse/partner  Marital separation/divorce  Financial difficulties  None of the above | 9.79  13.4  23.2  3.10  3.59  20.1  46.7 | 10.1  13.7  25.9  2.44  3.91  21.3  45.8 | 0.92  0.92  0.50  0.73  0.89  0.80  0.89 |
| Physical pain enough to interfere with daily life experienced in last month (%) (f.6159) | 75.4 | 75.9 | 0.89 |

Note: UK Biobank codes beginning with ‘f.’

**Supplementary Table S9**. Demographics of all prospective anxious (denoted by an ICD-10 diagnosis F40-3) against matched control cohorts.

|  | **Resilient average (n= 3,701)** | **All prospective average (n= 3,701)** | **Unadjusted p-value** |
| --- | --- | --- | --- |
| Age, years (f.21003) | 57.3 | 57.1 | 0.17 |
| Sex, % female (f.31) | 66.6 | 66.9 | 0.82 |
| Townsend Deprivation Index (f.22189) | -1.06 | -1.07 | 0.88 |
| Ethnicity, % Caucasian (f.21000) | 96.5 | 96.4 | 0.97 |
| Number of self-reported non-cancer illnesses (f.135)  % Diabetes (f.2443)  % Angina (f.6150)  % Heart attack (f.6150)  % Hypertension (f.6150) | 2.43  5.82  2.98  2.35  31.7 | 2.40  5.90  2.76  1.49  30.8 | 0.46  0.70  0.86  0.03  0.74 |
| Cancer (%) (f.2453) | 9.05 | 8.60 | 0.72 |
| Number of medications taken (f.137) | 3.125 | 3.117 | 0.91 |
| BMI (f.21001) | 27.94 | 27.85 | 0.45 |
| Smoking status (%) (f.20116)  Current  Previous  Never | 13.5  37.2  49.3 | 14.3  36.8  48.8 | 0.65 |
| Alcohol intake frequency (%) (f.1558)  Daily or almost daily  3-4 per week  1-2 per week  1-3 per month  Special occasions only  Never | 19.4  18.8  24.6  12.3  15.3  9.63 | 19.2  19.0  24.5  12.3  15.1  9.87 | 0.99 |
| Overall health rating (%) (f.2178)  Excellent  Good  Fair  Poor | 9.25  52.5  30.8  7.49 | 9.34  53.7  29.1  7.85 | 0.45 |
| Long-standing illness or disability (%) (f.2188) | 43.4 | 43.5 | 0.99 |
| Employment status (%) (f.6142)  Employed  Retired  Looking after home/family  Sickness/disability prevents work  Unemployed  Unpaid/voluntary work  Other | 48.3  41.2  6.15  6.59  1.65  5.10  1.30 | 50.4  39.6  5.75  7.13  1.79  4.18  1.38 | 0.18  0.35  0.71  0.60  0.84  0.16  0.88 |
| Stress/trauma in past 2 years (%) (f.6145)  Serious illness/injury/assault  Serious illness/injury/assault close relative  Death of close relative  Death of spouse/partner  Marital separation/divorce  Financial difficulties  None of the above | 12.0  11.8  23.5  1.93  3.987  18.1  48.4 | 11.0  12.5  23.6  2.04  3.989  18.5  48.7 | 0.38  0.64  0.93  0.88  0.94  0.85  0.90 |
| Physical pain enough to interfere with daily life experienced in last month (%) (f.6159) | 72.0 | 71.9 | 0.52 |

Note: UK Biobank codes beginning with ‘f.’

**Supplementary Table S10.** Comparison of trauma history experiences between anxious and lifetime anxiety free control cohorts.

| Variable | **Control cohort average (n= 10,939)** | **Anxious cohort average (n= 996)** | **Unadjusted p-value** |
| --- | --- | --- | --- |
| Felt loved as a child (f.20489.0.0)  Very often true  Often  Sometimes true  Rarely true  Never true | 58.2  27.5  11.5  2.1  0.07 | 44.3  21.8  22.1  8.69  3.03 | <2.2x10^-16^ |
| Physically abused by family as a child (f.20488.0.0)  Never true  Rarely true  Sometimes true  Often  Very often true | 85.2  9.89  4.39  0.44  0.11 | 75.1  11.7  9.49  2.22  1.51 | <2.2x10^-16^ |
| Felt hated by family member as a child (f.20487.0.0)  Never true  Rarely true  Sometimes true  Often  Very often true | 91.9  4.25  3.05  0.48  0.29 | 75.8  5.96  10.6  3.54  4.14 | <2.2x10^-16^ |
| Sexually molested as a child (f.20490.0.0)  Never true  Rarely true  Sometimes true  Often  Very often true | 94.8  3.21  1.73  0.19  0.08 | 86.4  6.21  5.19  1.11  1.11 | <2.2x10^-16^ |
| Physical violence by partner or ex-partner as an adult (f.20523.0.0)  Never true  Rarely true  Sometimes true  Often  Very often true | 93.0  4.12  2.36  0.27  0.27 | 79.6  7.67  7.97  1.51  3.23 | <2.2x10^-16^ |
| Belittlement by partner or ex-partner as an adult (f.20521.0.0)  Never true  Rarely true  Sometimes true  Often  Very often true | 88.5  6.29  4.30  0.58  0.38 | 64.3  9.62  16.1  4.67  5.36 | <2.2x10^-16^ |
| Victim of sexual assault (f.20531.0.0)  Never  Yes, but not in the last 12 months  Yes, within the last 12 months | 91.8  8.12  0.05 | 78.1  21.6  0.31 | <2.2x10^-16^ |
| Victim of physically violent crime (f.20529.0.0)  Never  Yes, but not in the last 12 months  Yes, within the last 12 months | 83.8  15.9  0.27 | 78.2  21.0  0.81 | 1.53x10^-10^ |
| Been in serious accident believed to be life-threatening (f.20526.0.0)  Never  Yes, but not in the last 12 months  Yes, within the last 12 months | 92.2  7.67  0.16 | 90.0  9.77  0.20 | 3.82x10^-5^ |
| Witnessed sudden violent death (f.20530.0.0)  Never  Yes, but not in the last 12 months  Yes, within the last 12 months | 87.5  11.9  0.53 | 84.4  15.1  0.50 | 0.003 |
| Been involved in combat or exposed to war-zone (f.20527.0.0)  Never  Yes, but not in the last 12 months  Yes, within the last 12 months | 96.3  3.68  0.06 | 97.3  2.72  0.00 | 0.02 |

Note: Chi-squared tests performed to calculate p-values. UK Biobank codes beginning with ‘f.’

**Supplementary Table S11.** Applied numerical coding of answers to trauma history questions in the UK Biobank study, with higher scores corresponding to worse traumatic outcomes.

| **Trauma question** | **UK Biobank response options** | **Numerical coding** |
| --- | --- | --- |
| Felt loved as a child (f.20489.0.0) | Prefer not to answer | 0 |
|  | Very often true | 1 |
|  | Often | 2 |
|  | Sometimes true | 3 |
|  | Rarely true | 4 |
|  | Never true | 5 |
| Physically abused by family as a child (f.20488.0.0)  Felt hated by family member as a child (f.20487.0.0)  Sexually molested as a child (f.20490.0.0)  Physical violence by partner or ex-partner as an adult (f.20523.0.0)  Belittlement by partner or ex-partner as an adult (f.20521.0.0) | Prefer not to answer | 0 |
|  | Never true | 1 |
|  | Rarely true | 2 |
|  | Sometimes true | 3 |
|  | Often | 4 |
|  | Very often true | 5 |
| Victim of sexual assault (f.20531.0.0)  Victim of physically violent crime (f.20529.0.0)  Been in serious accident believed to be life-threatening (f.20526.0.0)  Witnessed sudden violent death (f.20530.0.0)  Been involved in combat or exposed to war-zone (f.20527.0.0) | Prefer not to answer | 0 |
|  | Never | 1 |
|  | Yes, but not in the last 12 months | 3 |
|  | Yes, within the last 12 months | 5 |

Note: UK Biobank codes beginning with ‘f.’


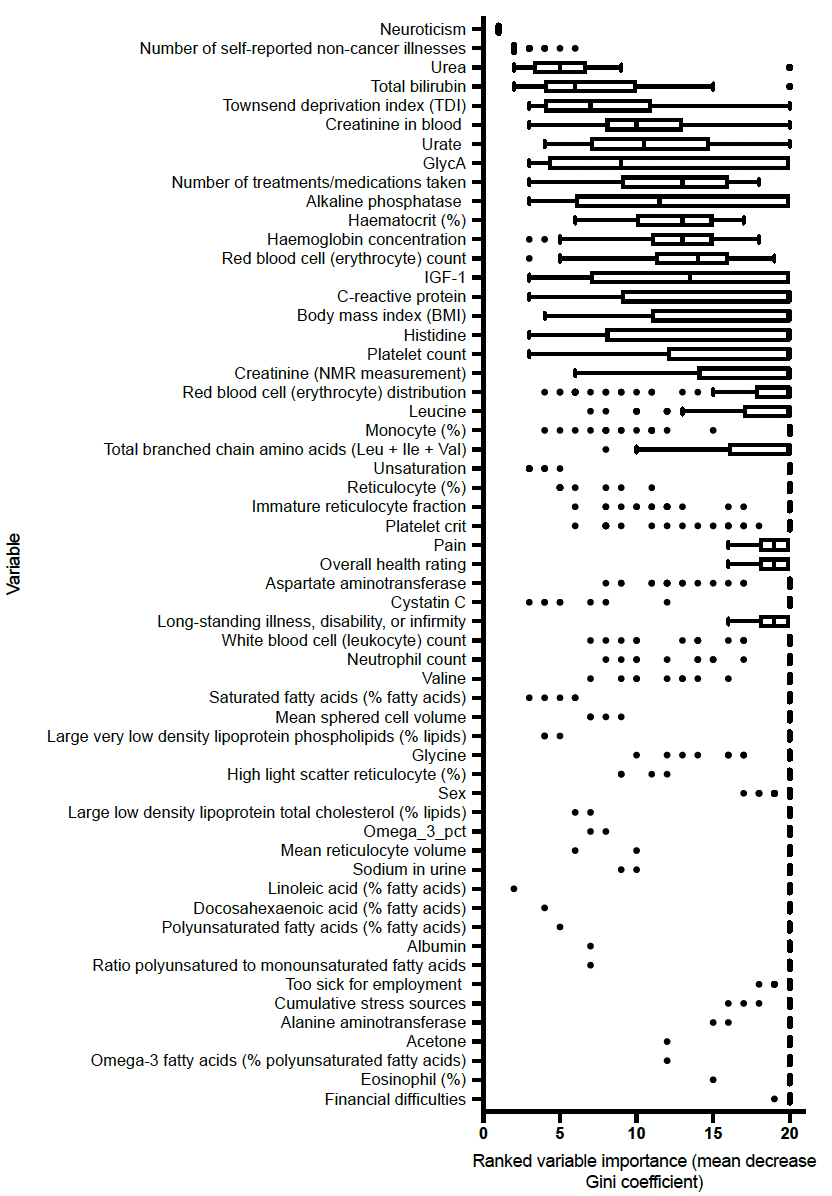


**Supplementary Figure S4.** Ranked variable importance based on random forest algorithm applied to 1–5-year prospective anxious cohort compared to the unmatched control cohort, using psychosocial and biological predictors.

**Supplementary Table S12.** Comparison of most important features driving random forest accuracy distinguishing ‘1-5-year prospective anxiety’ against unmatched controls.

| **Variable** | **Mean in control (n= 44,816)** | **Mean in 1-5 year prospective anxious (n= 612)** | **P-value** | **ROC AUC** | **Cohen’s d [95% confidence interval]** |
| --- | --- | --- | --- | --- | --- |
| Neuroticism score (f.20127) | 2.94 | 6.77 | <2.2x10^-16^ | *NA* | *NA* |
| Number of self-reported non-cancer illnesses (f.135) | 1.43 | 2.70 | <2.2x10^-16^ | *NA* | *NA* |
| Number of treatments/medications taken (f.137) | 1.85 | 3.54 | <2.2x10^-16^ | *NA* | *NA* |
| Total bilirubin (**µmol/L)**  (f.30840) | 8.345 | 9.644 | 3.27x10^-13^ | 0.61 | -0.28 [-0.36 to -0.20] |
| Townsend deprivation index (f.189) | -1.62 | -0.64 | 5.68x10^-13^ | *NA* | *NA* |
| Creatinine (**µmol/L)** measured by enzymatic analysis (f.30700) | 73.5 | 69.6 | 5.48x10^-8^ | 0.58 | -0.20 [-0.28 to -0.13] |
| Creatinine (mmol/L)  measured by NMR  (f.23478) | 0.067 | 0.064 | 4.65x10^-5^ | 0.56 | -0.15 [-0.23 to -0.07] |
| Urea (mmol/L)  (f.30670) | 5.46 | 5.22 | 4.67x10^-6^ | 0.56 | -0.18 [-0.26 to -0.10] |
| Alkaline phosphatase (U/L)  (f.30610) | 82.3 | 87.2 | 2.34x10^-5^ | 0.56 | 0.19 [0.11 to 0.27] |
| Urate (**µmol/L)** (f.30880) | 318.1 | 300.3 | 4.67x10^-6^ | 0.56 | -0.22 [-0.30 to -0.14] |
| Body mass index (BMI) (f.21001) | 27.3 | 28.6 | 2.44x10^-8^ | *NA* | *NA* |
| Monocyte (%) (f.30190) | 7.21 | 6.82 | 0.00037 | 0.55 | -0.14 [-0.22 to -0.06] |
| IGF-1 (nmol/L) (f.30770) | 21.7 | 20.4 | 5.78x10^-7^ | 0.57 | -0.23 [-0.31 to -0.15] |
| GlycA (mmol/L)  (f.23480) | 0.78 | 0.82 | 6.54x10^-13^ | 0.60 | 0.35 [0.27 to 0.43] |
| Histidine (mmol/L)  (f.23463) | 0.064 | 0.062 | 7.52x10^-7^ | 0.56 | -0.21 [-029 to -0.13] |
| C-reactive protein (mg/L)  (f.30710) | 3.27 | 3.72 | 0.14 | 0.59 | 0.05 [-0.03 to 0.13] |
| Haemoglobin concentration (g/dL)  (f.30020) | 14.3 | 13.9 | 1.64x10^-15^ | 0.60 | -0.35 [-0.43 to-0.27] |
| Red blood cell (erythrocyte) count (10^12^ cells/L) (f.30010) | 4.56 | 4.42 | 9.64x10^-16^ | 0.60 | -0.35 [-0.43 to -0.27] |
| Haematocrit (%) (f.30030) | 41.4 | 40.3 | 3.08x10^-14^ | 0.60 | -0.33 [-0.41 to -0.25] |
| Red blood cell (erythrocyte) distribution width (%)  (f.30070) | 13.4 | 13.6 | 0.00053 | 0.55 | 0.17 [0.09 to 0.25] |
| Platelet count (10^9^ cells/L)  (f.30080) | 250.2 | 264.9 | 8.25x10^-7^ | 0.57 | 0.25 [0.17 to 0.33] |
| Platelet crit (%)  (f.30090) | 0.23 | 0.24 | 8.03x10^-8^ | 0.58 | 0.28 [0.20 to 0.36] |
| Leucine (mmol/L)  (f.23466) | 0.10 | 0.097 | 0.00012 | 0.56 | -0.17 [-0.25 to -0.09] |
| Total concentration of branched-chain amino acids (mmol/L)  (f.23464) | 0.36 | 0.34 | 0.00033 | 0.55 | -0.16 [-0.24 to -0.08] |
| Sex (% female) (f.31) | 46.1 | 68.6 | 6.68x10^-13^ | *NA* | *NA* |
| Overall health rating (%) (f.2178)  Excellent  Good  Fair  Poor | 21.6  61.5  15.2  1.71 | 7.35  47.0  32.9  12.8 | <2.2x10^-16^ | *NA* | *NA* |
| Physical pain enough to interfere with daily life experienced in last month (%)  (f.6159) | 53.3 | 76.2 | <2.2x10^-16^ | *NA* | *NA* |
| Long-standing illness or disability (%) (f.2188) | 23.5 | 48.4 | <2.2x10^-16^ | *NA* | *NA* |
| Too sick for employment (%) (f.6142) | 1.20 | 12.5 | <2.2x10^-16^ | *NA* | *NA* |

Note: Sample sizes differ from original cohort creation due to the quality control procedure discussed in Methods 3.3.1. Chi-squared tests were employed to calculate p-values for categorical data. T-tests were employed to calculate p-values for numerical data, with Benjamini-Hochberg correction for multiple comparisons. Cohen’s d analysis performed to determine effect size, negative values indicative of lower levels in anxious individuals compared to control, positive values indicative of higher levels in anxious individuals compared to control.

**Supplementary Table S13.** Odds ratios of biomarkers important to the accuracy of random forest prediction of anxiety disorder.

| Variable | Threshold biomarker value for ‘1-5-year prospective anxiety’ against unmatched control | ‘1-5-year prospective anxiety’ against unmatched control, odds ratio (95% CI) | Threshold biomarker value for ‘all year prospective anxiety’ against unmatched control | ‘All year prospective anxiety’ against unmatched control, odds ratio (95% CI) |
| --- | --- | --- | --- | --- |
| Haemoglobin concentration (g/dL) | 14.0 | 0.51 (0.43 to 0.59) | 14.1 | 0.56 (0.53 to 0.60) |
| Haematocrit (%) | 41.2 | 0.54 (0.46 to 0.64) | 41.2 | 0.65 (0.61 to 0.69) |
| Red blood cell (erythrocyte) count (10^12^ cells/L) | 4.47 | 0.52 (0.44 to 0.61) | 4.47 | 0.60 (0.56 to 0.64) |
| Red blood cell (erythrocyte) distribution width (%) | 13.4 | 1.35 (1.15 to 1.58) | 13.4 | 1.12 (1.05 to 1.20) |
| Urate (**µmol/L)** | 309.0 | 0.72 (0.62 to 0.85) | 306.8 | 0.70 (0.66 to 0.75) |
| C-reactive protein (mg/L) | 1.47 | 1.47 2.13 (1.81 to 2.51) | 1.49 | 1.47 (1.37 to 1.57) |
| GlycA (mmol/L) | 0.80 | 1.84 (1.57 to 2.15) | 0.79 | 1.36 (1.27 to 1.45) |


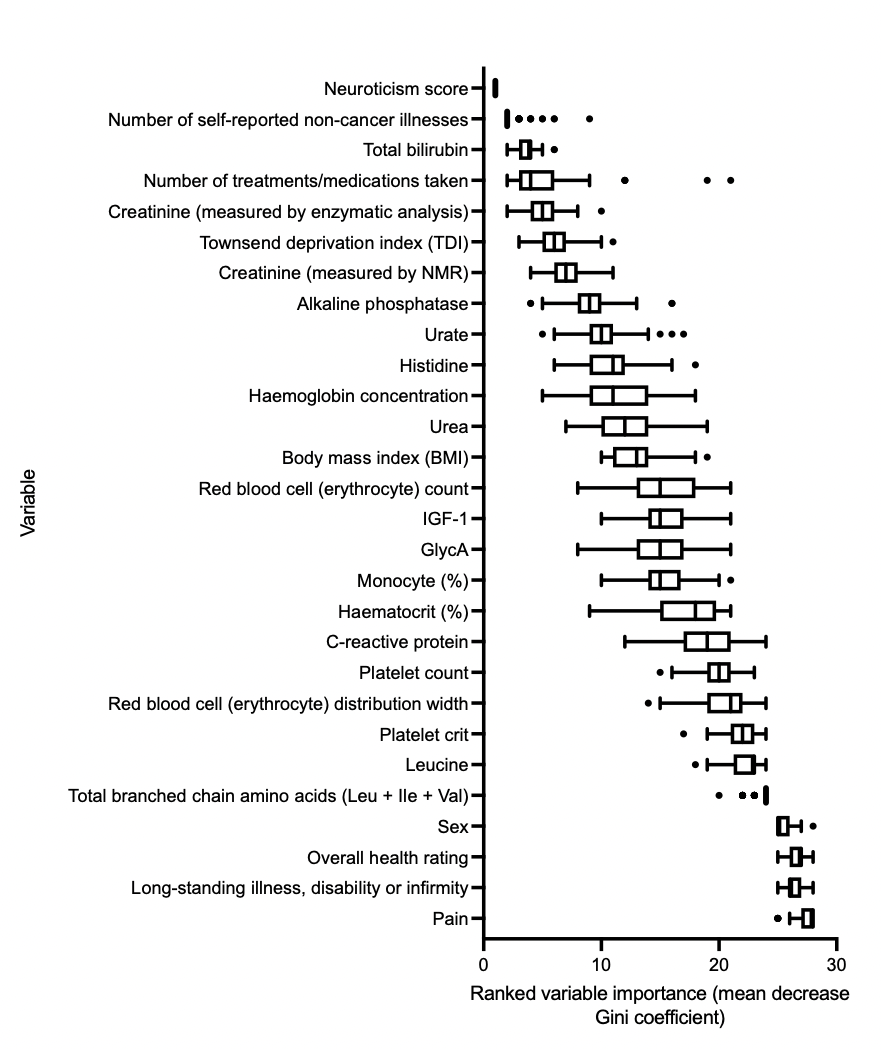


**Supplementary Figure S5.** Ranked variable importance based on random forest algorithm applied to the ‘all year prospective anxious’ cohort compared to the unmatched control cohort, using psychosocial and biological predictors.

**Supplementary Table S14.** Comparison of most important features driving random forest accuracy distinguishing ‘all year prospective anxiety’ against unmatched controls.

| **Variable** | **Mean in control (n= 44,816)** | **Mean in all year prospective anxious (n= 3,664)** | **P value** | **ROC AUC** | **Cohen’s d [95% confidence interval]** |
| --- | --- | --- | --- | --- | --- |
| Neuroticism score (f.20127) | 2.94 | 6.28 | <2.2x10^-16^ | *NA* | *NA* |
| Number of self-reported non-cancer illnesses (f.135) | 1.43 | 2.47 | <2.2x10^-16^ | *NA* | *NA* |
| Number of treatments/medications taken (f.137) | 1.85 | 3.21 | <2.2x10^-16^ | *NA* | *NA* |
| Total bilirubin (**µmol/L)**  (f.30840) | 9.84 | 8.57 | <2.2x10^-16^ | 0.58 | -0.26 [-0.33 to -0.22] |
| Townsend deprivation index (f.189) | -1.62 | -1.02 | <2.2x10^-16^ | *NA* | *NA* |
| Creatinine (**µmol/L)** measured by enzymatic analysis (f.30700) | 73.8 | 70.1 | <2.2x10^-16^ | 0.58 | -0.20 [-0.23 to -0.17] |
| Creatinine (mmol/L)  measured by NMR  (f.23478) | 0.07 | 0.06 | <2.2x10^-16^ | 0.56 | -0.16 [-0.19 to -0.13] |
| Urea (mmol/L)  (f.30670) | 5.45 | 5.33 | 2.75x10^-7^ | 0.53 | -0.09 [-0.12 to -0.05] |
| Alkaline phosphatase (U/L)  (f.30610) | 82.3 | 86.8 | <2.2x10^-16^ | 0.55 | 0.17 [0.14 to 0.20] |
| Urate (**µmol/L)** (f.30880) | 314.4 | 301.3 | <2.2x10^-16^ | 0.55 | -0.16 [-0.20 to -0.13] |
| Body mass index (BMI) (f.21001) | 27.3 | 27.9 | 8.68x10^-12^ | *NA* | *NA* |
| Monocyte (%) (f.30190) | 7.17 | 6.91 | 3.15x10^-8^ | 0.54 | -0.10 [-0.13 to -0.06] |
| IGF-1 (nmol/L) (f.30770) | 21.8 | 21.2 | 1.60x10^-11^ | 0.54 | -0.12 [-0.15 to -0.08] |
| GlycA (mmol/L)  (f.23480) | 0.78 | 0.80 | <2.2x10^-16^ | 0.55 | 0.19 [0.15 to 0.22] |
| Histidine (mmol/L)  (f.23463) | 0.064 | 0.063 | 1.35x10^-11^ | 0.54 | -0.12 [-0.15 to -0.08] |
| C-reactive protein (mg/L)  (f.30710) | 2.38 | 2.96 | 2.29x10^-15^ | 0.55 | 0.14 [0.10 to 0.17] |
| Haemoglobin concentration (g/dL)  (f.30020) | 14.3 | 14.0 | <2.2x10^-16^ | 0.59 | -0.30 [-0.34 to -0.27] |
| Red blood cell (erythrocyte) count (10^12^ cells/L) (f.30010) | 4.56 | 4.46 | <2.2x10^-16^ | 0.57 | -0.24 [-0.27 to -0.20] |
| Haematocrit (%) (f.30030) | 41.5 | 40.6 | <2.2x10^-16^ | 0.58 | -0.26 [-0.34 to 0.23] |
| Red blood cell (erythrocyte) distribution width (%)  (f.30070) | 13.45 | 13.51 | 9.61x10^-4^ | 0.52 | 0.06 [0.02 to 0.09] |
| Platelet count (10^9^ cells/L)  (f.30080) | 251.2 | 260.5 | 1.07x10^-19^ | 0.54 | 0.16 [0.12 to 0.19] |
| Platelet crit (%)  (f.30090) | 0.23 | 0.24 | <2.2x10^-16^ | 0.55 | 0.18 [0.15 to 0.21] |
| Leucine (mmol/L)  (f.23466) | 0.102 | 0.098 | 7.35x10^-10^ | 0.54 | -0.12 [-0.15 to -0.09] |
| Total concentration of branched-chain amino acids (mmol/L)  (f.23464) | 0.36 | 0.35 | 7.27x10^-10^ | 0.54 | -0.11 [-0.14 to -0.07] |
| Sex (% female) (f.31) | 46.1 | 57.0 | 0.007 | *NA* | *NA* |
| Overall health rating (%) (f.2178)  Excellent  Good  Fair  Poor | 21.6  61.5  15.2  1.71 | 9.16  52.8  29.0  8.98 | <2.2x10^-16^ | *NA* | *NA* |
| Physical pain enough to interfere with daily life experienced in last month (%)  (f.6159) | 53.3 | 72.4 | <2.2x10^-16^ | *NA* | *NA* |
| Long-standing illness or disability (%) (f.2188) | 23.5 | 44.5 | <2.2x10^-16^ | *NA* | *NA* |
| Too sick for employment (%) (f.6142) | 1.20 | 8.46 | <2.2x10^-16^ | *NA* | *NA* |

Note: Sample sizes differ from original cohort creation due to the quality control procedure discussed in Methods 3.3.1. Chi-squared tests were employed to calculate p-values for categorical data. T-tests were employed to calculate p-values for numerical data, with Benjamini-Hochberg correction for multiple comparisons. Cohen’s d analysis performed to determine effect size, negative values indicative of lower levels in anxious individuals compared to control, positive values indicative of higher levels in anxious individuals compared to control.

**B**

**A**


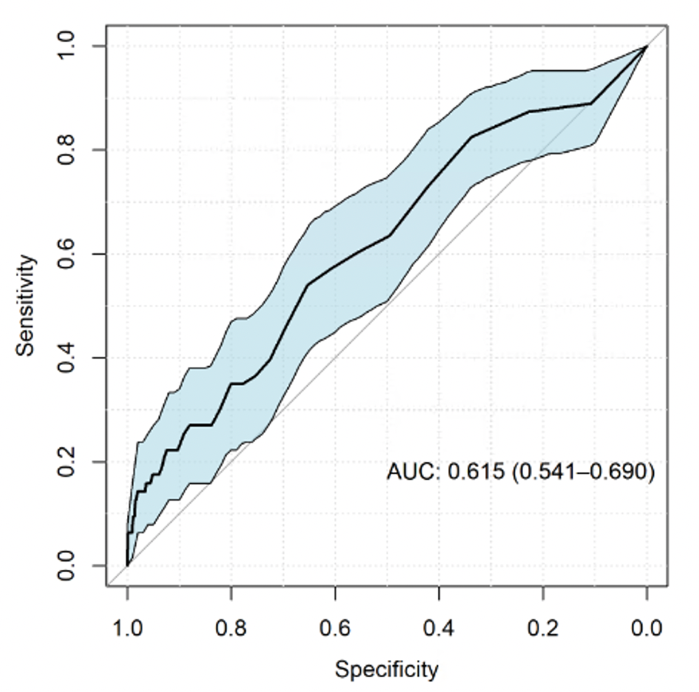


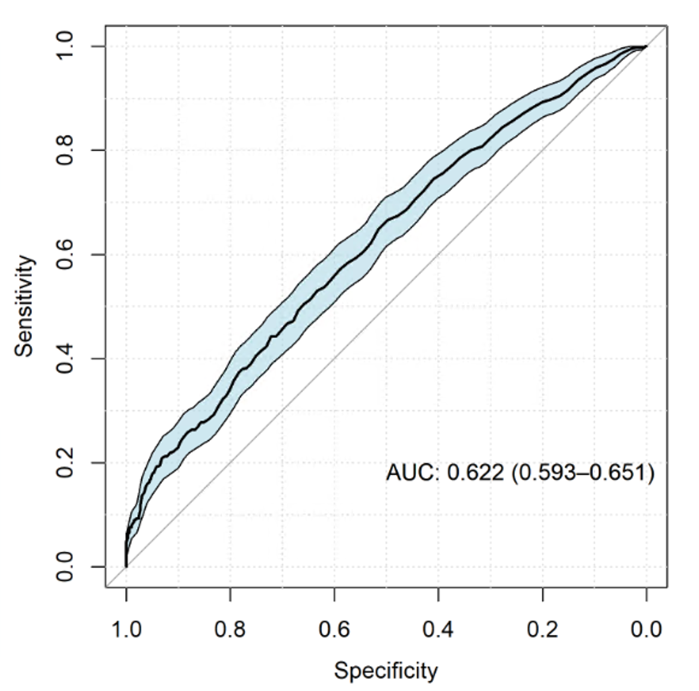


**Supplementary Figure S6.** Fair discrimination ability of random forest models distinguishing between individuals diagnosed with anxiety after sampling compared to unmatched lifetime anxiety free controls, using only blood biomarkers as predictors. A) ROC curve generated from final independent test of ‘1-5-year prospective anxious’ cohort against lifetime anxiety free controls. B) ROC curve generated from final independent test of ‘all prospective anxious’ cohort against lifetime anxiety free controls.


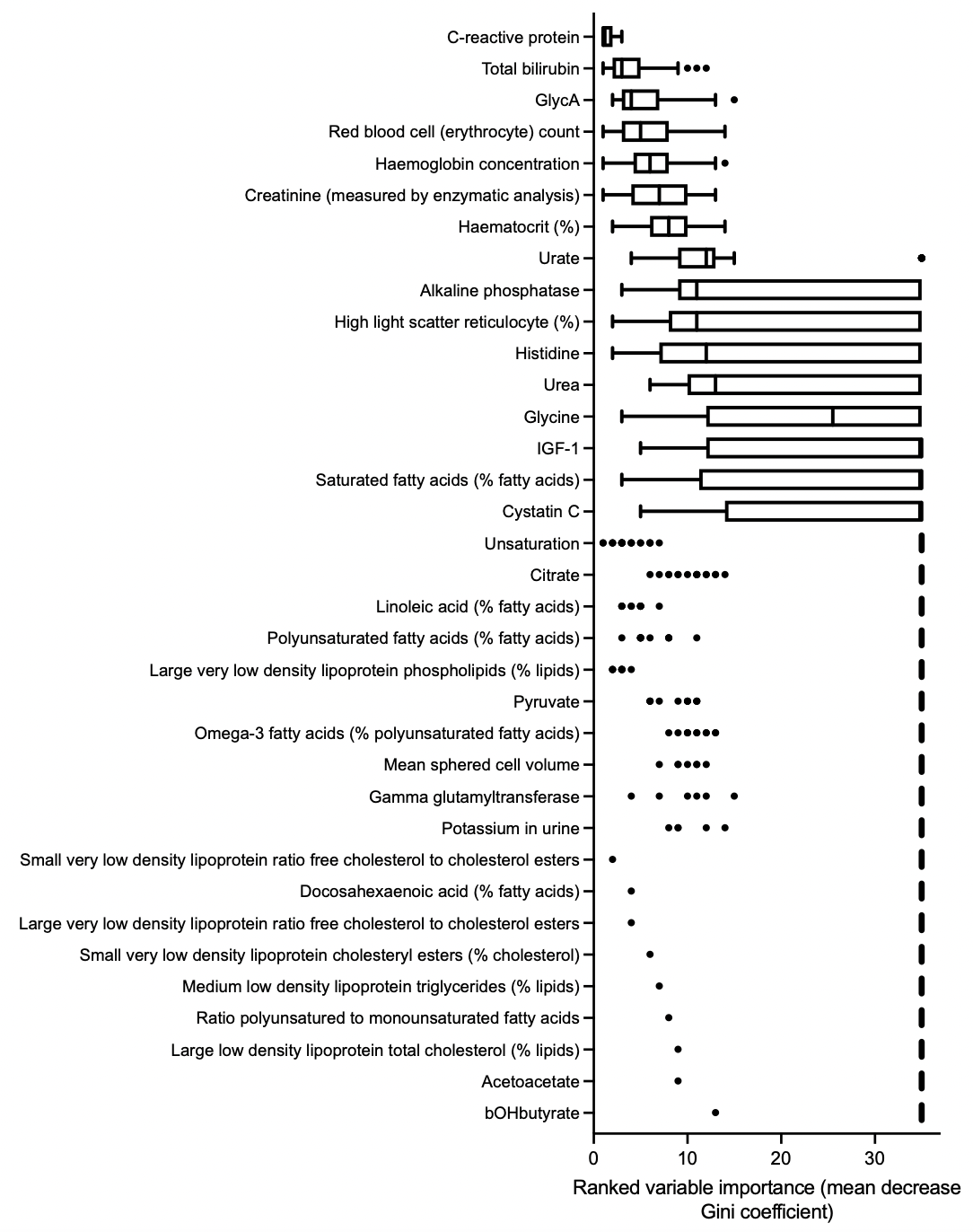


Variable

**Supplementary Figure S7.** Ranked variable importance based on random forest algorithm applied to the ‘1-5-year prospective anxious’ cohort compared to the unmatched control cohort, using solely biological predictors.


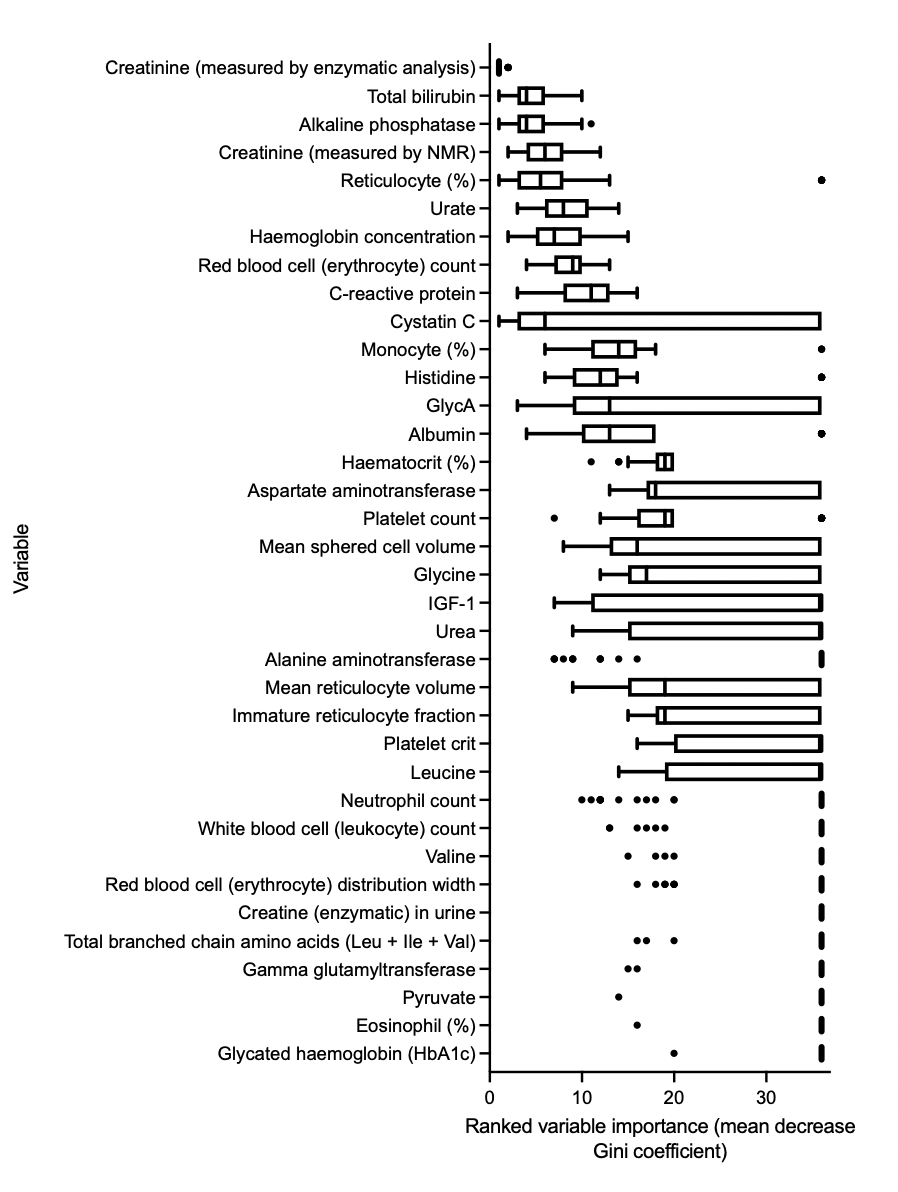


**Supplementary Figure S8.** Ranked variable importance based on random forest algorithm applied to the ‘all year prospective anxious’ cohort compared to the unmatched control cohort, using solely biological predictors.

**Supplementary Table S15.** Biomarkers that differ between the low and high extremes of neuroticism.

|  | **Mean in low extreme (n= 23,898)** | **Mean in high extreme (n= 1,813)** | **Typical reference range** | **ROC auc** | **Cohen’s d [95% confidence interval]** | **Adjusted p-value** |
| --- | --- | --- | --- | --- | --- | --- |
| \| Urate (μmol/L) \| \| --- \| | 320.43 | 299.63 | Males: 200-430  Females: 140-360 (1) | 0.60 | 0.33 [0.28 to 0.39] | 2.92 x 10^-41^ |
| \| Haematocrit (%) \| \| --- \| | 41.68 | 40.68 | 35.4 - 47.2 (2) | 0.59 | 0.30 [0.25 to 0.34] | 1.32 x 10^-29^ |
| \| Haemoglobin concentration (g/dL) \| \| --- \| | 14.43 | 14.06 | 12.1 – 16.3 (3) | 0.58 | 0.29 [0.24 to 0.33] | 8.25 x 10^-28^ |
| \| Creatinine (μmol/L) (measured by enzymatic analysis) \| \| --- \| | 74.5 | 70.1 | Males: 59-104  Females: 45-84  (4) | 0.60 | 0.28 [0.23 to 0.33] | 2.92 x 10^-41^ |
| \| Red blood cell (erythrocyte) count (10^12^ cells/L) \| \| --- \| | 4.57 | 4.47 | 3.9 - 5.5 (5) | 0.58 | 0.25 [0.20 to 0.30] | 4.25 x 10^-23^ |
| \| Glycine (mmol/L) \| \| --- \| | 0.16 | 0.17 | 0.10 – 0.40 (6) | 0.56 | -0.24 [-0.29 to -0.19] | 2.25 x 10^-16^ |
| \| Platelet crit (%) \| \| --- \| | 0.23 | 0.24 | 0.22- 0.24 (7) | 0.56 | -0.22 [-0.27 to -0.17] | 3.17 x 10^-14^ |
| Creatinine (mmol/L) (measured by NMR) | 0.068 | 0.065 | *NA* | 0.57 | 0.21 [0.16 to 0.25] | 1.60 x 10^-22^ |

Note: P-values determined using unpaired two tailed t-tests, with Benjamini-Hochberg correction for multiple comparisons. Cohen’s d analysis performed to determine effect size, positive values indicative of higher levels in low neurotic individuals compared to high neurotic individuals, negative values indicate lower levels in low neurotic individuals compared to highly neurotic.

References:

(1) <https://www.gloshospitals.nhs.uk/our-services/services-we-offer/pathology/tests-and-investigations/uric-acid-urate/>

(2) <https://biobank.ndph.ox.ac.uk/showcase/field.cgi?id=30030>

(3) <https://biobank.ndph.ox.ac.uk/showcase/field.cgi?id=30020>

(4) <https://www.nbt.nhs.uk/severn-pathology/requesting/test-information/creatinine#:~:text=Reference%20range%3A%20Male%20Adult%3A%2059,45%20%2D%2084%20%C2%B5mol%2FL>.

(5) <https://biobank.ndph.ox.ac.uk/showcase/field.cgi?id=30010>

(6) <https://www.southtees.nhs.uk/services/pathology/tests/glycine-csf-and-plasma/>

(7) <https://healthmatters.io/understand-blood-test-results/plateletcrit#:~:text=The%20normal%20range%20for%20PCT,inversely%20related%20to%20platelet%20counts>.

**Supplementary Table S16.** Biomarkers that differ between highly neurotic lifetime anxiety free control individuals and highly neurotic anxious individuals.

|  | **Mean in highly neurotic control (n= 946)** | **Mean in highly neurotic anxious (n= 867)** | **Typical reference range** | **ROC auc** | **Cohen’s d [95% confidence interval]** | **Adjusted p-value**   \|  \| \| --- \| |
| --- | --- | --- | --- | --- | --- | --- | --- |
| Red blood cell (erythrocyte) count (10^12^ cells/L) | 4.53 | 4.41 | 3.9 - 5.5 (1) | 0.60 | 0.33 [0.24 to 0.43] | 4.43x10^-10^ |
| Haematocrit (%) | 41.18 | 40.13 | 35.4 - 47.2 (2) | 0.60 | 0.33 [0.24 to 0.42] | \| 4.43x10^-10^ \| \| --- \| |
| Haemoglobin concentration (g/dL) | 14.23 | 13.87 | 12.1 - 16.3 (3) | 0.59 | 0.29 [0.20 to 0.38] | \| 1.03x10^-7^ \| \| --- \| |
| \| Triglycerides in IDL (mmol/L) \| \| --- \| | 0.096 | 0.10 | *NA* | 0.57 | -0.26 [-0.35 to -0.17] | \| 4.13x10^-6^ \| \| --- \| |
| \| High Density Lipoprotein Cholesteryl esters (% cholesterol) \| \| --- \| | 77.90 | 77.58 | *NA* | 0.56 | 0.25 [0.15 to 0.34] | \| 1.46x10^-5^ \| \| --- \| |
| \| High Density Lipoprotein Ratio free cholesterol to cholesterol esters \| \| --- \| | 0.28 | 0.29 | *NA* | 0.56 | -0.25 [-0.34 to -0.15] | \| 1.46x10^-5^ \| \| --- \| |
| \| High Density Lipoprotein Free cholesterol (% cholesterol) \| \| --- \| | 22.10 | 22.42 | *NA* | 0.56 | -0.24 [-0.34 to -0.15] | \| 1.72x10^-5^ \| \| --- \| |
| \| Extra small Very Low Density Lipoprotein (VLDL) Triglycerides (% lipids) \| \| --- \| | 0.067 | 0.072 | *NA* | 0.57 | -0.24 [-0.33 to -0.15] | \| 1.90x10^-5^ \| \| --- \| |
| \| Small High Density Lipoprotein Cholesteryl esters (% lipids) \| \| --- \| | 28.65 | 28.21 | *NA* | 0.56 | 0.24 [0.14 to 0.33] | \| 2.15x10^-5^ \| \| --- \| |
| \| Small High Density Lipoprotein Cholesteryl esters (% cholesterol) \| \| --- \| | 74.35 | 73.93 | *NA* | 0.57 | 0.24 [0.14 to 0.33] | \| 2.15x10^-5^ \| \| --- \| |
| \| Small High Density Lipoprotein Free cholesterol (% cholesterol) \| \| --- \| | 25.65 | 26.07 | *NA* | 0.57 | -0.24 [-0.33 to -0.14] | \| 2.15x10^-5^ \| \| --- \| |
| \| Large Low Density Lipoprotein Triglycerides (mmol/L) \| \| --- \| | 0.093 | 0.010 | *NA* | 0.57 | -0.23 [-0.33 to -0.14] | \| 2.15x10^-5^ \| \| --- \| |
| \| Medium High Density Lipoprotein Ratio free cholesterol to cholesterol esters \| \| --- \| | 0.209 | 0.214 | *NA* | 0.56 | -0.23 [-0.33 to -0.14] | \| 2.15x10^-5^ \| \| --- \| |
| \| Small High Density Lipoprotein Ratio free cholesterol to cholesterol esters \| \| --- \| | 0.346 | 0.354 | *NA* | 0.57 | -0.23 [-0.33 to -0.14] | \| 2.15x10^-5^ \| \| --- \| |
| \| Omega-6 fatty acids (% fatty acids) \| \| --- \| | 38.56 | 37.73 | *NA* | 0.56 | 0.23 [0.14 to 0.32] | \| 2.15x10^-5^ \| \| --- \| |
| \| Linoleic acid (% fatty acids) \| \| --- \| | 29.57 | 28.79 | *NA* | 0.56 | 0.23 [0.14 to 0.32] | \| 2.15x10^-5^ \| \| --- \| |
| \| Medium High Density Lipoprotein Free cholesterol (% cholesterol) \| \| --- \| | 17.32 | 17.58 | *NA* | 0.56 | -0.23 [-0.32 to -0.14] | \| 2.15x10^-5^ \| \| --- \| |
| \| Medium High Density Lipoprotein Cholesteryl esters (% cholesterol) \| \| --- \| | 82.68 | 82.42 | *NA* | 0.56 | 0.23 [0.14 to 0.32] | \| 2.15x10^-5^ \| \| --- \| |
| \| Extra small Very Low Density Lipoprotein Phospholipids (mmol/L) \| \| --- \| | 0.10 | 0.11 | *NA* | 0.56 | -0.23 [-0.32 to -0.14] | \| 2.52x10^-5^ \| \| --- \| |
| \| Extra small Very Low Density Lipoprotein Total lipids (mmol/L) \| \| --- \| | 0.35 | 0.37 | *NA* | 0.56 | -0.22 [-0.32 to -0.13] | \| 3.98x10^-5^ \| \| --- \| |
| \| Low Density Lipoprotein Triglycerides (mmol/L) \| \| --- \| | 0.14 | 0.15 | *NA* | 0.56 | -0.22 [-0.31 to -0.12] | \| 8.07x10^-5^ \| \| --- \| |
| \| GlycA (mmol/L) \| \| --- \| | 0.79 | 0.81 | *NA* | 0.56 | -0.22 [-0.31 to -0.12] | \| 8.07x10^-5^ \| \| --- \| |
| \| Total bilirubin (μmol/L) \| \| --- \| | 9.15 | 8.31 | < 21 (4) | 0.58 | 0.21 [0.12 to 0.31] | \| 8.65x10^-5^ \| \| --- \| |
| \| High Density Lipoprotein Triglycerides (mmol/L) \| \| --- \| | 0.14 | 0.15 | *NA* | 0.56 | -0.21 [-0.31 to -0.12] | \| 0.0001 \| \| --- \| |
| \| Polyunsaturated fatty acids (% fatty acids) \| \| --- \| | 42.8 | 42.0 | *NA* | 0.56 | 0.21 [0.12 to 0.31] | \| 0.0001 \| \| --- \| |
| \| Medium High Density Lipoprotein Triglycerides (mmol/L) \| \| --- \| | 0.052 | 0.056 | *NA* | 0.56 | -0.21 [-0.30 to -0.12] | \| 0.0001 \| \| --- \| |
| \| Extra Large High Density Lipoprotein Triglycerides (mmol/L) \| \| --- \| | 0.0068 | 0.0074 | *NA* | 0.56 | -0.21 [-0.30 to -0.12] | \| 0.0001 \| \| --- \| |
| \| Extra Small Very Low Density Lipoprotein Particle concentration (mmol/L) \| \| --- \| | 5.42 x 10^-5^ | 5.71 x 10^-5^ | *NA* | 0.56 | -0.21 [-0.30 to -0.12] | \| 0.0001 \| \| --- \| |
| \| Large High Density Lipoprotein Triglycerides (mmol/L) \| \| --- \| | 0.029 | 0.031 | *NA* | 0.56 | -0.21 [-0.30 to -0.12] | \| 0.0001 \| \| --- \| |
| \| Saturated fatty acids (mmol/L) \| \| --- \| | 3.98 | 4.17 | *NA* | 0.55 | -0.21 [-0.30 to -0.12] | \| 0.0001 \| \| --- \| |
| \| Small High Density Lipoprotein Total cholesterol (% lipids) \| \| --- \| | 38.51 | 38.13 | *NA* | 0.55 | 0.21 [0.12 to 0.30] | \| 0.0001 \| \| --- \| |
| \| Ratio polyunsaturated to monounsaturated fatty acids \| \| --- \| \|  \| | 1.87 | 1.80 | *NA* | 0.56 | 0.20 [0.11 to 0.30] | \| 0.0002 \| \| --- \| |
| Extra small Very Low Density Lipoprotein Free cholesterol (mmol/L) | 0.057 | 0.060 | *NA* | 0.55 | -0.20 [-0.29 to -0.11] | 0.0002 |

Note: P-values determined using unpaired two tailed t-tests, with Benjamini-Hochberg correction for multiple comparisons. Cohen’s d analysis performed to determine effect size, positive values indicative of higher levels in high neurotic individuals without anxiety disorder compared to high neurotic individuals with anxiety, negative values indicate lower levels in high neurotic individuals without anxiety compared to highly neurotic with anxiety.

References:

1. <https://biobank.ndph.ox.ac.uk/showcase/field.cgi?id=30010>

2. <https://biobank.ndph.ox.ac.uk/showcase/field.cgi?id=30030>

3. <https://biobank.ndph.ox.ac.uk/showcase/field.cgi?id=30020>

4. <https://www.nbt.nhs.uk/severn-pathology/requesting/test-information/bilirubin#:~:text=Reference%20range%3A%20Up%20to%201,%3A%20%3C%2021%20%C2%B5mol%2FL>.

**Supplementary Table S17.** Blood biomarker comparison between individuals who have experienced a high amount of trauma against a low amount of trauma.

|  | **Average low trauma (n= 3,689)** | **Average high trauma (n= 204)** | **Cohen’s d [95% confidence interval]** | **Adjusted p-value** |
| --- | --- | --- | --- | --- |
| Reticulocyte (%) | 1.29 | 1.55 | 0.33 [0.19 to 0.47] | 0.002 |
| High light scatter reticulocyte (%) | 0.37 | 0.43 | 0.32 [0.18 to 0.46] | 0.002 |
| High light scatter reticulocyte count (10^12^ cells/L) | 0.017 | 0.020 | 0.31 [0.17 to 0.45] | 0.002 |
| High density lipoprotein size | 9.68 | 9.61 | -0.30 [-0.45 to -0.16] | 0.002 |
| Extra large high density lipoprotein free cholesterol (mmol/L) | 0.025 | 0.023 | -0.30 [-0.44 to -0.16] | 0.002 |
| Extra large high density lipoprotein total cholesterol (mmol/L) | 0.09 | 0.08 | -0.30 [-0.44 to -0.15] | 0.002 |
| Extra large high density lipoprotein phospholipids (mmol/L) | 0.09 | 0.07 | -0.29 [-0.44 to -0.15] | 0.002 |
| Extra large high density lipoprotein cholesterol esters (mmol/L) | 0.06 | 0.05 | -0.29 [-0.44 to -0.15] | 0.002 |
| Extra large high density lipoprotein total lipids (mmol/L) | 0.18 | 0.16 | -0.29 [-0.43 to -0.15] | 0.002 |
| Extra large high density lipoprotein particle concentration (mmol/L) | 0.00025 | 0.00022 | -0.29 [-0.43 to -0.15] | 0.002 |
| Reticulocyte count (10^12^ cells/L) | 0.06 | 0.07 | 0.29 [0.15 to 0.43] | 0.002 |
| Medium high density lipoprotein free cholesterol (% lipids) | 8.37 | 8.17 | -0.29 [-0.43 to -0.15] | 0.002 |
| Large high density lipoprotein total cholesterol (mmol/L) | 0.32 | 0.27 | -0.29 [-0.43 to -0.15] | 0.002 |
| Large high density lipoprotein cholesterol esters (mmol/L) | 0.25 | 0.21 | -0.29 [-0.43 to -0.15] | 0.002 |
| Ratio polyunsaturated to monounsaturated fatty acids | 1.90 | 1.80 | -0.29 [-0.43 to -0.14] | 0.002 |
| White blood cell (leukocyte) count (10^9^ cells/L) | 6.61 | 7.08 | 0.28 [0.14 to 0.42] | 0.002 |
| Monounsaturated fatty acids (% fatty acids) | 23.06 | 23.78 | 0.28 [0.14 to 0.42] | 0.002 |
| Neutrophil count (10^9^ cells/L) | 4.03 | 4.40 | 0.28 [0.14 to 0.42] | 0.002 |
| High density lipoprotein free cholesterol (% cholesterol) | 9.74 | 9.59 | -0.28 [0.42 to -0.14] | 0.002 |
| Large high density lipoprotein total lipids (mmol/L) | 0.69 | 0.60 | -0.28 [0.42 to -0.14] | 0.002 |
| Large high density lipoprotein free cholesterol (mmol/L) | 0.07 | 0.06 | -0.27 [-0.42 to -0.13] | 0.002 |
| Large high density lipoprotein triglycerides (% lipids) | 5.29 | 6.16 | 0.27 [0.13 to 0.42] | 0.002 |
| Large high density lipoprotein particle concentration (mmol/L) | 0.002 | 0.001 | -0.27 [-0.41 to -0.13] | 0.002 |
| GlycA (mmol/L) | 0.77 | 0.80 | 0.27 [0.13 to 0.41] | 0.002 |
| Medium high density lipoprotein phospholipids (% lipids) | 47.02 | 47.35 | 0.26 [0.12 to 0.40] | 0.004 |
| Polyunsaturated fatty acids (% fatty acids) | 43.01 | 42.08 | -0.26 [-0.40 to -0.12] | 0.004 |
| Immature reticulocyte fraction | 0.28 | 0.30 | 0.26 [0.12 to 0.40] | 0.004 |
| Large high density lipoprotein phospholipids (mmol/L) | 0.34 | 0.30 | -0.26 [-0.40 to -0.12] | 0.005 |
| High density lipoprotein total cholesterol (% lipids) | 43.79 | 43.11 | -0.25 [-0.39 to -0.11] | 0.006 |
| Medium high density lipoprotein total cholesterol (% lipids) | 47.75 | 47.03 | -0.25 [-0.39 to -0.11] | 0.007 |
| Urea (mmol/L) | 5.35 | 5.03 | -0.25 [-0.39 to -0.11] | 0.007 |
| Large high density lipoprotein free cholesterol (% lipids) | 10.18 | 9.91 | -0.25 [-0.39 to -0.10] | 0.008 |
| Unsaturation | 1.37 | 1.35 | -0.24 [-0.39 to -0.10] | 0.008 |
| Platelet count (10^9^ cells/L) | 250.46 | 263.52 | 0.23 [0.09 to 0.38] | 0.012 |
| Large high density lipoprotein total cholesterol (% lipids) | 44.73 | 43.51 | -0.23 [-0.38 to -0.09] | 0.012 |
| High density lipoprotein phospholipids (% lipids) | 51.42 | 51.70 | 0.23 [0.09 to 0.37] | 0.013 |
| Platelet crit (%) | 0.23 | 0.24 | 0.23 [0.09 to 0.37] | 0.013 |
| Small high density lipoprotein free cholesterol (% lipids) | 9.98 | 9.86 | -0.23 [-0.37 to -0.09] | 0.013 |
| Large very low density lipoprotein cholesteryl esters (% lipids) | 54.19 | 53.13 | -0.23 [-0.37 to -0.09] | 0.014 |
| Large very low density lipoprotein free cholesterol (% cholesterol) | 45.81 | 46.87 | 0.23 [0.09 to 0.37] | 0.014 |
| High density lipoprotein total cholesterol (mmol/L) | 1.33 | 1.26 | -0.23 [-0.37 to -0.09] | 0.014 |
| High density lipoprotein cholesterol esters (mmol/L) | 1.04 | 0.98 | -0.23 [-0.37 to -0.08] | 0.015 |
| High density lipoprotein triglycerides (% lipids) | 4.80 | 5.18 | 0.22 [0.08 to 0.37] | 0.016 |
| Ratio triglycerides to phosphoglycerides | 0.56 | 0.60 | 0.22 [0.08 to 0.36] | 0.016 |
| Large low density lipoprotein ratio free cholesterol to cholesterol esters | 0.36 | 0.35 | -0.22 [-0.36 to -0.08] | 0.016 |
| Medium high density lipoprotein triglycerides (% lipids) | 5.23 | 5.63 | 0.22 [0.08 to 0.36] | 0.017 |
| Very low density lipoprotein size | 38.50 | 38.77 | 0.22 [0.08 to 0.36] | 0.017 |
| Small high density lipoprotein triglycerides (mmol/L) | 0.05 | 0.05 | 0.22 [0.08 to 0.36] | 0.017 |
| Low density lipoprotein free cholesterol (% cholesterol) | 0.37 | 0.36 | -0.22 [-0.36 to -0.08] | 0.017 |
| High density lipoprotein free cholesterol (mmol/L) | 0.30 | 0.28 | -0.22 [-0.36 to -0.08] | 0.018 |
| Triglycerides (% lipids) | 14.05 | 15.11 | 0.22 [0.08 to 0.36] | 0.018 |
| Total bilirubin (μmol/L) | 9.40 | 8.45 | -0.22 [-0.36 to -0.08] | 0.018 |
| Large low density lipoprotein cholesteryl esters (% cholesterol) | 73.66 | 73.96 | 0.22 [0.08 to 0.36] | 0.018 |
| Large low density lipoprotein free cholesterol (% cholesterol) | 26.34 | 26.04 | -0.22 [-0.36 to -0.08] | 0.018 |
| Large very low density lipoprotein phospholipids (% lipids) | 18.69 | 19.51 | 0.21 [0.07 to 0.36] | 0.020 |
| Large low density lipoprotein free cholesterol (% lipids) | 18.79 | 18.53 | -0.21 [-0.35 to -0.07] | 0.020 |
| Large very low density lipoprotein ratio free cholesterol to cholesterol esters | 0.86 | 0.89 | 0.21 [0.07 to 0.35] | 0.020 |
| High density lipoprotein cholesteryl esters (% lipids) | 34.03 | 33.52 | -0.21 [-0.35 to -0.07] | 0.020 |
| Large high density lipoprotein cholesteryl esters (% lipids) | 34.55 | 33.60 | -0.21 [-0.35 to -0.07] | 0.020 |

Note: P-values determined using unpaired two tailed t-tests, with Benjamini-Hochberg correction for multiple comparisons. Cohen’s d analysis performed to determine effect size, positive values indicative of lower values in low trauma cohort compared to high trauma cohort, negative values indicative of higher values in low trauma cohort compared to high trauma cohort.

**Supplementary Table S18.** Comparing blood biomarker levels between anxious individuals and lifetime anxiety free controls, who have all experienced a high amount of trauma.

|  | **Average control (n= 99)** | **Average anxious (n= 105)** | **Cohen’s d [95% confidence interval]** | **Adjusted p-value** |
| --- | --- | --- | --- | --- |
| Haemoglobin concentration (g/dL) | 14.4 | 13.6 | -0.62 [-0.90 to -0.34] | 0.01 |
| Haematocrit (%) | 41.6 | 39.6 | -0.59  [-0.87 to -0.30] | 0.01 |
| Red blood cell (erythrocyte) count (10^12^ cells/L) | 4.59 | 4.39 | -0.48 [-0.76 to -0.20] | 0.09 |
| Neutrophil count (10^9^ cells/L) | 4.05 | 4.72 | 0.44 [0.16 to 0.72] | 0.18 |
| Monocyte (%) | 7.07 | 6.25 | -0.39 [-0.67 to -0.11] | 0.36 |
| Basophil count (10^9^ cells/L) | 0.03 | 0.05 | 0.39  [0.11 to 0.67] | 0.36 |
| Neutrophil (%) | 59.7 | 62.8 | 0.37 [0.09 to 0.64] | 0.50 |
| White blood cell (leukocyte) count (10^9^ cells/L) | 6.74 | 7.40 | 0.36 [0.08 to 0.63] | 0.56 |
| Creatinine (μmol/L) (measured by enzymatic analysis) | 72.8 | 68.3 | -0.33 [-0.61 to -0.05] | 0.79 |
| Platelet crit (%) | 0.23 | 0.25 | 0.32 [0.04 to 0.60] | 0.88 |
| Lymphocyte (%) | 29.9 | 27.8 | -0.29 [-0.57 to -0.02] | 1.00 |
| Creatinine (mmol/L) (measured by NMR) | 0.07 | 0.06 | -0.29 [-0.57 to -0.02] | 1.00 |
| Platelet count (10^9^ cells/L) | 254.6 | 271.9 | 0.29 [0.01 to 0.56] | 1.00 |
| High light scatter reticulocyte (%) | 0.40 | 0.47 | 0.26 [-0.02 to 0.53] | 1.00 |
| C-reactive protein (mg/L) | 2.26 | 3.12 | 0.25 [-0.03 to 0.53] | 1.00 |
| Glycated haemoglobin (HbA1c) (mmol/mol) | 35.1 | 36.0 | 0.25 [-0.03 to 0.52] | 1.00 |
| Valine (mmol/L) | 0.21 | 0.20 | -0.23 [-0.51 to 0.04] | 1.00 |
| Mean sphered cell volume (fL) | 83.1 | 82.0 | -0.23 [-0.50 to 0.05] | 1.00 |
| Alanine aminotransferase (U/L) | 24.4 | 21.8 | -0.22 [-0.49 to 0.06] | 1.00 |
| Basophil (%) | 0.54 | 0.65 | 0.22 [-0.06 to 0.49] | 1.00 |
| Glycine (mmol/L) | 0.15 | 0.17 | 0.21 [-0.06 to 0.49] | 1.00 |
| Platelet distribution width (%) | 16.5 | 16.4 | -0.21[-0.48 to 0.07] | 1.00 |

Note: P-values determined using unpaired two tailed t-tests, with Benjamini-Hochberg correction for multiple comparisons. Cohen’s d analysis performed to determine effect size, positive values indicative of lower values in high trauma cohort without anxiety disorder compared to high trauma cohort with anxiety disorder, negative values indicative of higher values in high trauma cohort without anxiety disorder compared to high trauma cohort with anxiety disorder.


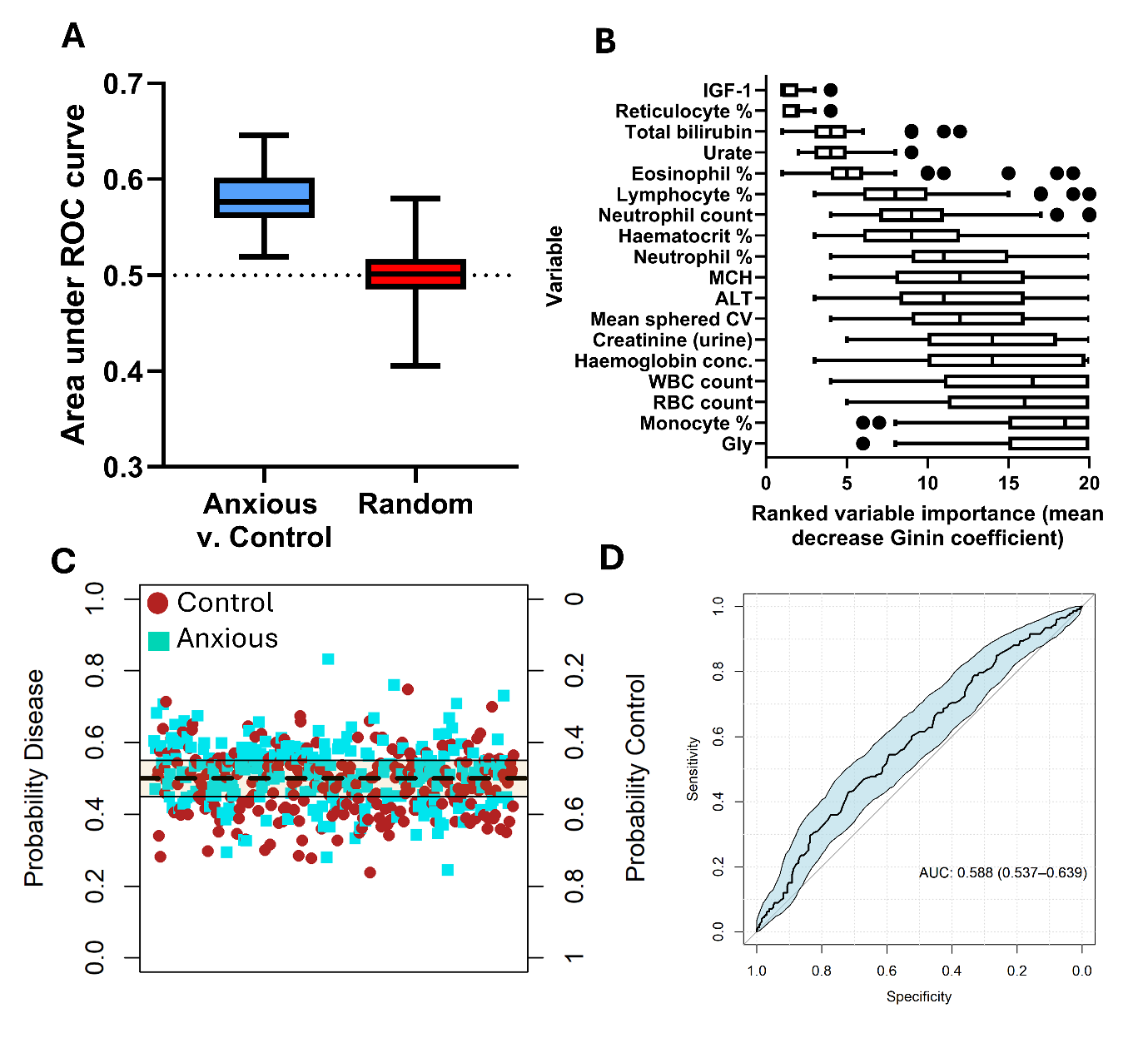


**FEMALE ANALYSIS: ANXIOUS PROSPECTIVE COHORT v. MATCHED RESILIENT CONTROLS**

**Supplementary Figure S9.** A) The random forest 10-fold cross validation distinguished the prospectively diagnosed anxiety cohort against the unmatched control group (mean ROC AUC: 0.58), compared to the null distribution model (ROC AUC: 0.5). B) Ranked variable important across the 100-model ensemble. C) Application of the trained random forest model to the independent final test data resulted in poor prediction discrimination between individuals with a prospectively-diagnosed anxiety disorder (true anxious cases labelled turquoise) against lifetime anxiety-free resilient control cases (true control cases labelled red). D) The ROC curve, summarising prediction quality at the independent testing stage, demonstrates that the model discriminated between anxious cases and controls with a ROC AUC of 0.59 (95% CI 0.54 – 0.64).


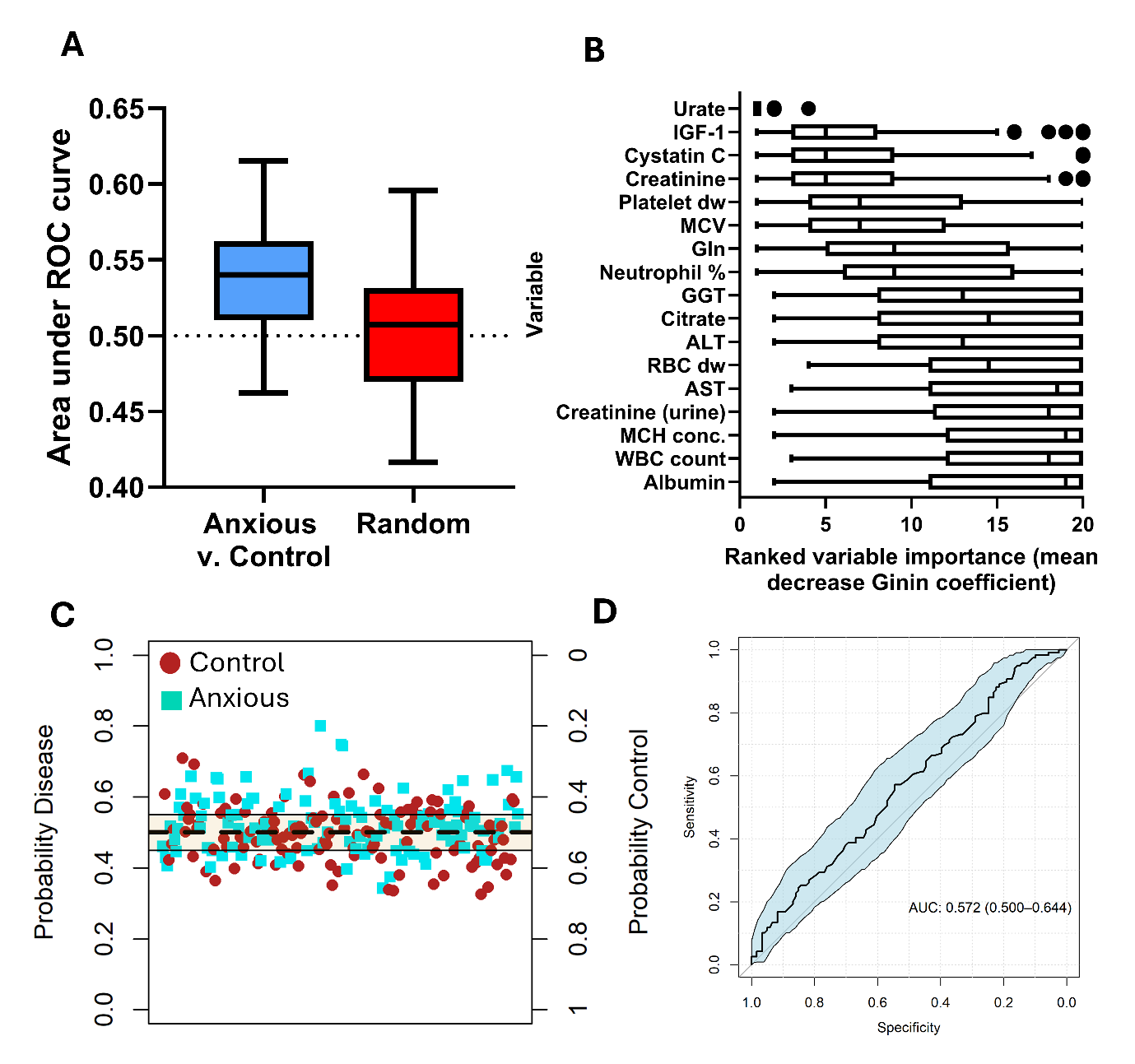


**MALE ANALYSIS: ANXIOUS PROSPECTIVE COHORT v. MATCHED RESILIENT CONTROLS**

**Supplementary Figure S10.** A) The random forest 10-fold cross validation distinguished the prospectively diagnosed anxiety cohort against the unmatched control group (mean ROC AUC: 0.57), compared to the null distribution model (ROC AUC: 0.51). B) Ranked variable important across the 100-model ensemble. C) Application of the trained random forest model to the independent final test data resulted in poor prediction discrimination between individuals with a prospectively-diagnosed anxiety disorder (true anxious cases labelled turquoise) against lifetime anxiety-free resilient control cases (true control cases labelled red). D) The ROC curve, summarising prediction quality at the independent testing stage, demonstrates that the model discriminated between anxious cases and controls with a ROC AUC of 0.57 (95% CI 0.50 – 0.64).


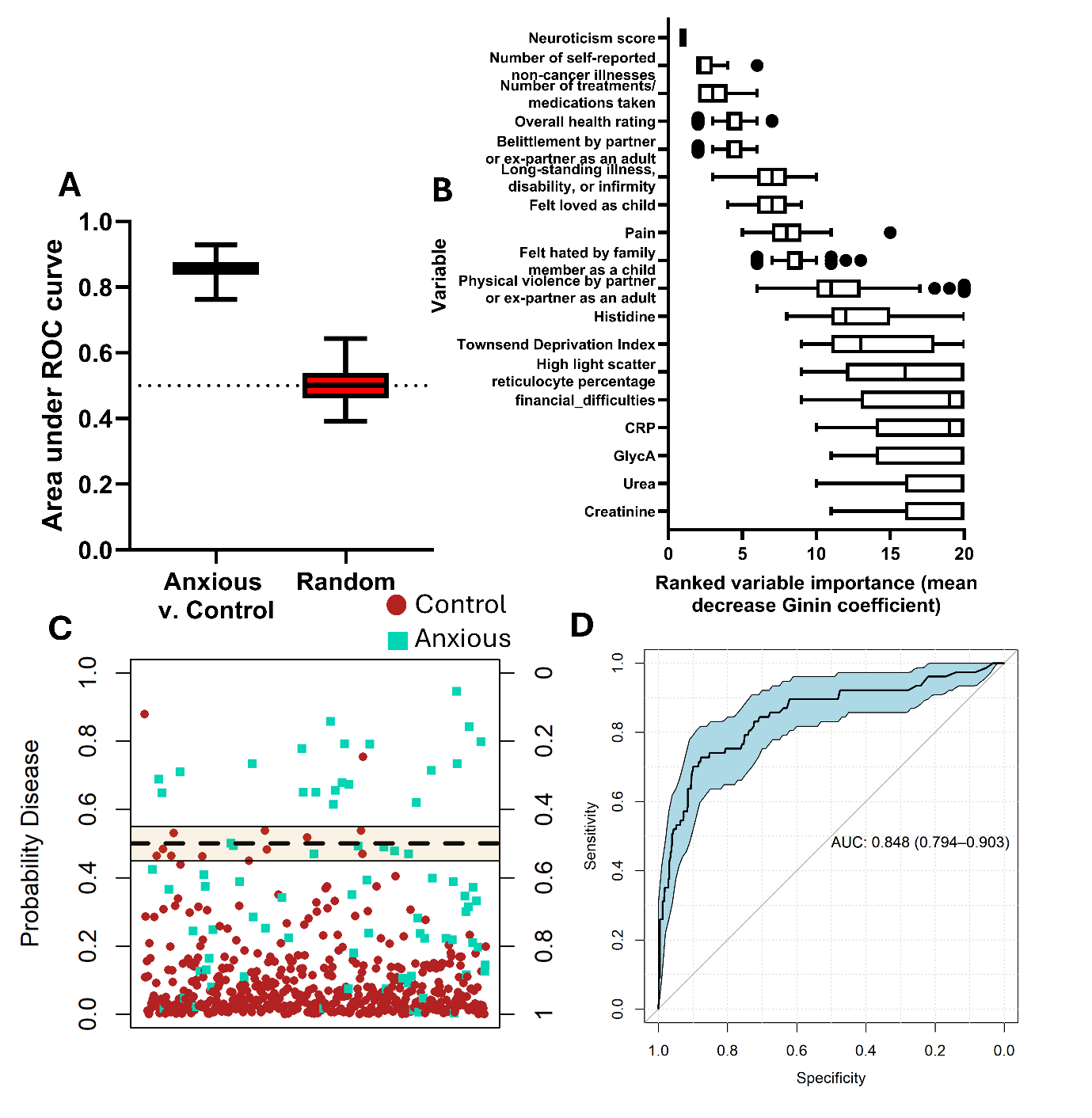


**FEMALE ANALYSIS: ANXIOUS PROSPECTIVE COHORT v. UNMATCHED CONTROLS [INCL. SELF-REPORTED DATA]**

**Supplementary Figure S11.** A) The random forest 10-fold cross validation distinguished the prospectively diagnosed anxiety cohort against the unmatched control group (mean ROC AUC: 0.86), compared to the null distribution model (ROC AUC: 0.50). B) Ranked variable important across the 100-model ensemble. C) Application of the trained random forest model to the independent final test data resulted in accurate prediction discrimination between individuals with a diagnosed anxiety disorder (true anxious cases labelled turquoise) against lifetime anxiety-free control cases (true control cases labelled red). D) The ROC curve, summarising prediction quality at the independent testing stage, demonstrates that the model discriminated between anxious cases and controls with a ROC AUC of 0.85 (95% CI 0.79 – 0.90).

**MALE ANALYSIS: ANXIOUS PROSPECTIVE COHORT v. UNMATCHED CONTROLS [INCL. SELF-REPORTED DATA]**


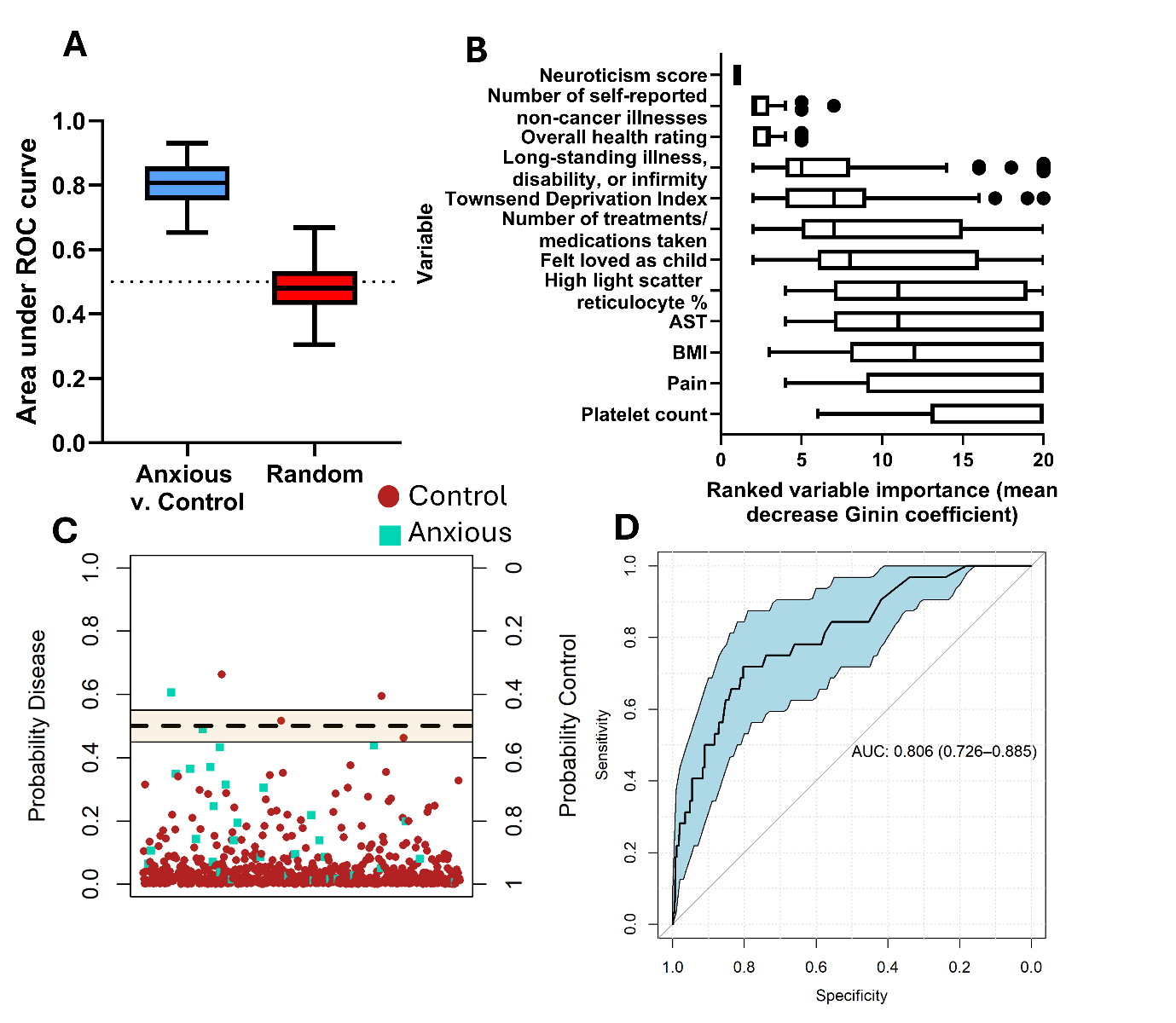


**Supplementary Figure S12.** A) The random forest 10-fold cross validation distinguished the prospectively diagnosed anxiety cohort against the unmatched control group (mean ROC AUC: 0.81), compared to the null distribution model (ROC AUC: 0.49). B) Ranked variable important across the 100-model ensemble. C) Application of the trained random forest model to the independent final test data resulted in accurate prediction discrimination between individuals with a diagnosed anxiety disorder (true anxious cases labelled turquoise) against lifetime anxiety-free control cases (true control cases labelled red). D) The ROC curve, summarising prediction quality at the independent testing stage, demonstrates that the model discriminated between anxious cases and controls with a ROC AUC of 0.81 (95% CI 0.73 – 0.89).
